# The effects of height and BMI on prostate cancer incidence and mortality: a Mendelian randomization study in 20,848 cases and 20,214 controls from the PRACTICAL consortium

**DOI:** 10.1007/s10552-015-0654-9

**Published:** 2015-09-19

**Authors:** Neil M. Davies, Tom R. Gaunt, Sarah J. Lewis, Jeff Holly, Jenny L. Donovan, Freddie C. Hamdy, John P. Kemp, Rosalind Eeles, Doug Easton, Zsofia Kote-Jarai, Ali Amin Al Olama, Sara Benlloch, Kenneth Muir, Graham G. Giles, Fredrik Wiklund, Henrik Gronberg, Christopher A. Haiman, Johanna Schleutker, Børge G. Nordestgaard, Ruth C. Travis, David Neal, Nora Pashayan, Kay-Tee Khaw, Janet L. Stanford, William J. Blot, Stephen Thibodeau, Christiane Maier, Adam S. Kibel, Cezary Cybulski, Lisa Cannon-Albright, Hermann Brenner, Jong Park, Radka Kaneva, Jyotsna Batra, Manuel R. Teixeira, Hardev Pandha, PRACTICAL consortium, Mark Lathrop, George Davey Smith, Richard M. Martin

**Affiliations:** School of Social and Community Medicine, University of Bristol, Bristol, UK; MRC Integrative Epidemiology Unit, University of Bristol, Bristol, UK; School of Clinical Sciences, University of Bristol, Bristol, BS10 5NB UK; Nuffield Department of Surgery, University of Oxford, Oxford, UK; University of Queensland Diamantina Institute, Translational Research Institute, Brisbane, QLD Australia; The Institute of Cancer Research, London, SM2 5NG UK; The Royal Marsden NHS Foundation Trust, London, SW3 6JJ UK; Strangeways Laboratory, Centre for Cancer Genetic Epidemiology, Department of Public Health and Primary Care, University of Cambridge, Worts Causeway, Cambridge, UK; Institute of Population Health, University of Manchester, Manchester, UK; Cancer Epidemiology Centre, The Cancer Council Victoria, 615 St Kilda Road, Melbourne, VIC Australia; Centre for Epidemiology and Biostatistics, Melbourne School of Population and Global Health, The University of Melbourne, Melbourne, VIC Australia; Department of Medical Epidemiology and Biostatistics, Karolinska Institute, Stockholm, Sweden; Department of Preventive Medicine, Keck School of Medicine, University of Southern California/Norris Comprehensive Cancer Center, Los Angeles, CA USA; Department of Medical Biochemistry and Genetics, University of Turku, Turku, Finland; Institute of Biomedical Technology/BioMediTech, University of Tampere and FimLab Laboratories, Tampere, Finland; Department of Clinical Biochemistry, Herlev Hospital, Copenhagen University Hospital, Herlev Ringvej 75, 2730 Herlev, Denmark; Cancer Epidemiology Unit, Nuffield Department of Clinical Medicine, University of Oxford, Oxford, UK; Surgical Oncology (Uro-Oncology: S4), University of Cambridge, Addenbrooke’s Hospital, Hills Road, Box 279, Cambridge, UK; Li Ka Shing Centre, Cancer Research UK Cambridge Research Institute, Cambridge, UK; Strangeways Laboratory, Centre for Cancer Genetic Epidemiology, Department of Oncology, University of Cambridge, Worts Causeway, Cambridge, UK; Cambridge Institute of Public Health, University of Cambridge, Forvie Site, Robinson Way, Cambridge, CB2 0SR UK; Division of Public Health Sciences, Fred Hutchinson Cancer Research Center, Seattle, WA USA; Department of Epidemiology, School of Public Health, University of Washington, Seattle, WA USA; International Epidemiology Institute, 1455 Research Blvd., Suite 550, Rockville, MD 20850 USA; Mayo Clinic, Rochester, MN USA; Department of Urology, University Hospital Ulm, Ulm, Germany; Institute of Human Genetics, University Hospital Ulm, Ulm, Germany; Brigham and Women’s Hospital/Dana-Farber Cancer Institute, 45 Francis Street-ASB II-3, Boston, MA 02115 USA; Washington University, St. Louis, Missouri; International Hereditary Cancer Center, Department of Genetics and Pathology, Pomeranian Medical University, Szczecin, Poland; Division of Genetic Epidemiology, Department of Medicine, University of Utah School of Medicine, Salt Lake City, UT USA; Division of Clinical Epidemiology and Aging Research, German Cancer Research Center (DKFZ), Heidelberg, Germany; Division of Preventive Oncology, German Cancer Research Center (DKFZ), Heidelberg, Germany; German Cancer Consortium (DKTK), German Cancer Research Center (DKFZ), Heidelberg, Germany; Division of Cancer Prevention and Control, H. Lee Moffitt Cancer Center, 12902 Magnolia Dr., Tampa, FL USA; Molecular Medicine Center and Department of Medical Chemistry and Biochemistry, Medical University Sofia, 2 Zdrave St, 1431 Sofia, Bulgaria; Australian Prostate Cancer Research Centre-Qld, Institute of Health and Biomedical Innovation and School of Biomedical Sciences, Queensland University of Technology, Brisbane, QLD Australia; Department of Genetics, Portuguese Oncology Institute, Porto, Portugal; Biomedical Sciences Institute (ICBAS), Porto University, Porto, Portugal; The University of Surrey, Guildford, Surrey GU2 7XH UK; Department of Applied Health Research, University College London, 1-19 Torrington Place, London, WC1E 7HB UK; Commissariat à l’Energie Atomique, Center National de Génotypage, Evry, France; McGill University-Génome Québec Innovation Centre, Montreal, Canada; Bristol Nutrition Biomedical Research Unit, National Institute for Health Research, Bristol, UK

**Keywords:** Height, Body mass index, Prostate cancer, Mendelian randomization, Single nucleotide polymorphisms, Instrumental variables analysis

## Abstract

**Background:**

Epidemiological studies suggest a potential role for obesity and determinants of adult stature in prostate cancer risk and mortality, but the relationships described in the literature are complex. To address uncertainty over the causal nature of previous observational findings, we investigated associations of height- and adiposity-related genetic variants with prostate cancer risk and mortality.

**Methods:**

We conducted a case–control study based on 20,848 prostate cancers and 20,214 controls of European ancestry from 22 studies in the PRACTICAL consortium. We constructed genetic risk scores that summed each man’s number of height and BMI increasing alleles across multiple single nucleotide polymorphisms robustly associated with each phenotype from published genome-wide association studies.

**Results:**

The genetic risk scores explained 6.31 and 1.46 % of the variability in height and BMI, respectively. There was only weak evidence that genetic variants previously associated with increased BMI were associated with a lower prostate cancer risk (odds ratio per standard deviation increase in BMI genetic score 0.98; 95 % CI 0.96, 1.00; *p* = 0.07). Genetic variants associated with increased height were not associated with prostate cancer incidence (OR 0.99; 95 % CI 0.97, 1.01; *p* = 0.23), but were associated with an increase (OR 1.13; 95 % CI 1.08, 1.20) in prostate cancer mortality among low-grade disease (*p* heterogeneity, low vs. high grade <0.001). Genetic variants associated with increased BMI were associated with an increase (OR 1.08; 95 % CI 1.03, 1.14) in all-cause mortality among men with low-grade disease (*p* heterogeneity = 0.03).

**Conclusions:**

We found little evidence of a substantial effect of genetically elevated height or BMI on prostate cancer risk, suggesting that previously reported observational associations may reflect common environmental determinants of height or BMI and prostate cancer risk. Genetically elevated height and BMI were associated with increased mortality (prostate cancer-specific and all-cause, respectively) in men with low-grade disease, a potentially informative but novel finding that requires replication.

**Electronic supplementary material:**

The online version of this article (doi:10.1007/s10552-015-0654-9) contains supplementary material, which is available to authorized users.

## Introduction

Prostate cancer is the most common male cancer in Europe and North America, but the robust identification of potentially modifiable risk factors has proven elusive [1]. Epidemiological studies suggest a potential role for obesity [2–5] and determinants of adult stature [6], but the relationships described in the literature are complex [7–9]. Inverse associations have generally been observed between adiposity and localized prostate cancer, but associations are largely positive with advanced or high-grade [2, 10] and fatal [3] cancer and may vary in direction depending on whether obesity was observed in early or middle to late adulthood [4]. Adult stature is generally positively associated with prostate cancer, although associations may be stronger for fatal [11] or high- compared with low-grade disease [6].

The explanation for these associations is unclear. Observations regarding obesity could be due to confounding by common causes of both obesity and prostate cancer (e.g., calorie and dietary fat intake) [12]; the mitogenic hormones insulin and insulin-like growth factor-I [13, 14]; delayed detection in obese men [8, 9]; or a real biological effect [15]. Observed height associations could reflect early-life environmental (e.g., fetal, dietary, social, hormones, and psychological circumstances) or shared genetic contributions to stature and prostate cancer risk [16–18].

Genetic epidemiological studies are less susceptible to confounding than observational epidemiology. This is because conditional on population structure, genetic variants are more likely to be independent of later environment and lifestyle factors [19]; they are also unlikely to be affected by reverse causation. Thus, the existence of genetic variation in obesity and height can provide robust evidence about how associations of phenotypes, in this case obesity and height, with diseases arise [15]. We previously reported that a single nucleotide polymorphism (SNP) associated with obesity (*FTO* rs9939609-A) was inversely associated with low-grade prostate cancer (odds ratio, OR 0.90 per A allele; 95 % CI 0.81, 0.99; *p* = 0.03), but positively associated with high-grade cancer (OR 1.16; 0.99, 1.37; *p* = 0.07) [15]. These data suggest that the comparable observational associations between adiposity phenotypes and prostate cancer outcomes are not confounded. However, the evidence for these effects was weak, originating from a single study of moderate size (1,550 cases) using only a single variant, and there is no evidence we are aware of linking genetic variation in height with prostate cancer. The results, therefore, require confirmation and extension in larger datasets, using height- and additional adiposity-related genetic variants.

Our aim was to use genetic variation in height and body mass index (BMI) as unconfounded exposures to investigate the causal associations of obesity and stature with prostate cancer risk and outcomes (Mendelian randomization [20]). Instead of the single-variant, single-sample approach used previously, we employ a more powerful two-sample, multiple-variant approach [21, 22] that combines several polymorphisms (based on confirmed genetic variant-intermediate phenotype associations [23, 24]) into genetic risk scores in order to explain more of the variance in BMI and height exposures and thus increase power and avoid weak instrument bias [21].

## Methods

Participants in this study were men of European genotypic ancestry from 22 independent studies contributing to the international PRACTICAL Consortium (PRostate cancer AssoCiation group To Investigate Cancer-Associated aLterations in the genome, http://www.practical.ccge.medschl.cam.ac.uk) [25, 26]. The individual studies are described at http://www.nature.com/ng/journal/v45/n4/extref/ng.2560-S1.pdf, with summary data in Table 1. Of the studies within the PRACTICAL Consortium at the time of data extraction, we excluded the EPIC-Norfolk, CAPS, and SEARCH studies (involving 3,005 cases and 2,825 controls), because they were included in the genome-wide studies that originally detected the height and BMI genetic variants [23, 24]. Cancers were categorized as low grade (Gleason score ≤ 6) or high grade (Gleason score ≥ 7) and localized (T1 or T2 on TNM staging, or if not available, “localized” on SEER staging) or advanced (T3 or T4 on TNM staging, or if not available, “regional” or “distant” on SEER staging). All studies met the appropriate ethical criteria for each country in accordance with the principles embodied in the Declaration of Helsinki.

**Table 1:** Clinical characteristics of the men in each of the studies contributing to the PRACTICAL consortium (*n* = 41,062)

Study	Country	*n*		Mean		%				
		Controls	Cases	Age at diagnosis (years)	PSA at diagnosis (ng/ml)	Screen detected^b^ (%)	Family history prostate cancer	Gleason score 8–10	Advanced stage (T3 or T4)	Distant spread (SEER)
CPCS1	Denmark	2,771	848	69.5	48.0	0.0	8.2	35.0	–	–
CPCS2	Denmark	1,009	265	64.9	36.0	0.0	14.7	10.6	–	–
EPIC	Europe^a^	1,079	722	64.9	19.7	0.0	–	3.6	3.8	0.9
ESTHER	Germany	318	313	65.5	58.7	61.9	8.9	9.1	26.4	3.4
FHCRC	USA	730	761	59.7	16.1	–	21.7	10.4	–	2.6
IPO-Porto	Portugal	66	183	59.3	8.3	82.8	20.0	15.8	64.5	0.0
MAYO	USA	488	767	65.2	15.5	73.7	29.1	33.0	44.4	0.5
MCCS	Australia	1,169	1,698	58.5	136.6	–	23.4	11.0	14.0	0.8
MEC	USA	829	819	69.5	–	–	13.0	36.0	–	2.8
MOFFITT	USA	100	412	64.9	7.3	0.0	22.9	11.2	3.5	0.5
PCMUS	Bulgaria	140	151	69.3	32.5	21.2	5.3	29.8	42.4	18.5
PPF-UNIS	UK	176	244	68.9	32.0	–	25.3	10.9	25.7	9.0
Poland	Poland	359	438	67.7	40.2	0.0	10.6	14.0	36.8	2.8
ProMPT	UK	1	166	66.3	33.0	0.0	34.6	18.9	32.7	7.8
ProtecT	UK	1,474	1,542	62.8	9.6	100.0	7.9	5.7	11.3	0.4
QLD/ProsCan	Australia	87	186	61.3	6.7	–	36.2	4.0	0.0	0.0
STHMI	Sweden	2,224	2,002	66.2	–	–	20.2	10.2	14.2	1.6
TAMPERE	Finland	2,413	2,753	68.2	69.1	46.8	–	15.4	21.0	7.3
UKGPCS	UK	4,182	4,549	63.8	83.9	28.9	23.4	17.2	32.9	10.7
ULM	Germany	354	601	63.8	19.1	–	44.9	15.5	39.9	1.1
UTAH	USA	245	440	62.6	–	–	51.4	16.1	–	4.7
WUGS	USA	0	988	60.8	6.2	–	42.3	7.9	24.2	0.1

Studies: Copenhagen Prostate Cancer Study 1 (CPCS1); Copenhagen Prostate Cancer Study 2 (CPCS2); European Prospective Investigation Into Cancer and Nutrition (EPIC); Epidemiological investigations of the chances of preventing, recognizing early and optimally treating chronic diseases in an elderly population (ESTHER); Fred Hutchinson Cancer Research Center (FHCRC); Portuguese Oncology Institute, Porto (IPO-Porto); Mayo Clinic (MAYO); Melbourne Collaborative Cohort Study (MCCS); Multiethnic Cohort Study (MEC); The Moffitt Group (MOFFITT); Prostate Cancer study Medical University Sofia (PCMUS); Prostate Project Foundation-Postgraduate Medical School, Surrey (PPF-UNIS); The Poland Group (Poland); Prostate cancer: Mechanisms of progression and Treatment (ProMPT); Prostate testing for cancer and Treatment (ProtecT); Retrospective Queensland Study (QLD) and the Prostate Cancer Supportive Care and Patient Outcomes Project (ProsCan); Stockholm 1 (STHMI); Finnish Genetic Predisposition to Prostate Cancer Study (TAMPERE); U.K. Genetic Prostate Cancer Study and The Prostate Cancer Research Foundation Study (UKGPCS); Familial Prostate Cancer Study Ulm (ULM); UTAH Study (UTAH); Washington University Genetics Study (WUGS)

^a^Germany, Greece, Italy, Netherlands, Spain, Sweden, Oxford

^b^Studies with 0 % screen detected are entirely based on clinically detected cases, and studies with no information about method of detection have a missing value; 12,231 individuals have information of method of detection

### Genotyping

Genotyping was carried out using an Illumina Custom Infinium genotyping array (iCOGS), designed for the Collaborative Oncological Gene-environment Study (COGS), and consisted of 211,155 SNPs (details at http://ec.europa.eu/research/health/medical-research/cancer/fp7-projects/cogs_en.html) [25, 26]. This array was devised to evaluate genetic variants for associations with breast, ovarian, and prostate cancer; 68,638 were specifically chosen for their potential relevance to prostate cancer. The remaining 125,877 SNPs measured by the array were chosen for relevance to other cancers and common SNPs which had been previously associated with any trait. Participants with low call rates (<95 %) and high or low heterozygosity (*p* < 1 × 10^−5^) were excluded; 201,598 SNPs passed quality control for the European ancestry samples. We used these genotypic data to impute 2.6 million SNPs based on the HapMap 2 CEU reference panel and using IMPUTE2 software [27]. We excluded poorly imputed SNPs (*R*^2^ < 0.3).

### Constructing genetic risk scores for BMI and height

We constructed genetic risk scores [21] for height and BMI using 179 and 32 variants, respectively, previously reported in genome-wide association studies (GWAS) to be associated with height [23] and BMI [24]. We used allele dosages from the imputation to construct the genetic risk score. The dosages code each SNP continuously from 0 to 2, and the dosages across all SNPs are summed to estimate the number of height or BMI increasing risk alleles per man. Each genetic variant was given a weight equal to the effect of the variant on height or BMI reported by the previous GWASs [23, 24]. The genetic risk score is therefore a weighted sum of the estimated number of risk alleles across several genotypes, which can improve the precision of the results compared to an unweighted score [21]. Supplementary Tables 1 and 2 provide details of the variants used and weights assigned.

### Statistical analysis

We estimated associations of the genetic risk scores with measured height and BMI using linear regression based on 1,270 men without prostate cancer [i.e., prostate-specific antigen (PSA) level <3.0 ng/ml or men with a raised PSA but who were biopsy negative] from the ProtecT population-based study [15, 28], one of the PRACTICAL studies with the relevant phenotypic data in a well-defined control group. We computed *F* statistics and *R*^2^ values (the proportion of variation in height and BMI explained by the genetic risk score) from the linear regression to evaluate the strength of the genetic risk score instruments in a population of men at increased risk of cancer. We had 82 and 78 % power to detect an odds ratio of 1.12 and 1.25 for the effects of height and BMI on prostate cancer risk, assuming a sample size of 41,062 and that the genetic risk scores explained 6.31 and 1.46 % of the variation in height and BMI, respectively [29].

We investigated associations of the phenotypes (height and BMI) and the genetic risk scores (for height and BMI) with measured covariables in the ProtecT cases to assess whether the scores were likely to be independent of potential environmental confounding factors and to assess the potential for pleiotropy (genetic confounding). We included the following potential confounders: diabetes; occupation (managerial vs. nonmanagerial); exercise (strenuous; moderate or strenuous, vs. light); alcohol intake (three or more drinks a week vs. two or less); smoking (passive, current, or ex-smoker vs. never); diagnostic PSA level; and age at recruitment. We investigated whether the scores predicted circulating insulin-like growth factor (IGF-I) levels (a potential mechanism linking size with prostate cancer [13, 14]) and benign prostatic hyperplasia (a potential cause of detection bias [30]).

We assessed the relationship of the height and BMI genetic risk scores with prostate cancer risk, stage, and grade across all 22 eligible studies contributing to PRACTICAL using logistic regression to compute ORs, with robust standard errors to account for within-study clustering. The genetic risk score was standardized to mean zero and standard deviation one, and the ORs were parameterized as the change in outcome per standard deviation increase in genetic risk score. In a secondary analysis, we also computed ORs comparing the highest versus the lowest quintile of each genetic risk score to illustrate the differences in outcomes between the extremes of the BMI or height allele score distributions. This reduced form, the association of the instrument (the genetic risk score) with the outcome, is a valid test of the direction of the effect of a phenotype on an outcome [31, 32]. We investigated between-study heterogeneity by estimating the logistic regressions individually for each study and using the Stata *metan* command to estimate the *I*^2^ statistic assuming a fixed-effect model. As we found little evidence of heterogeneity, we report the ORs from the logistic regression analyses conducted across the 22 included studies.

We calculated ORs for all prostate cancers and then separately for localized versus advanced and low-grade (Gleason score ≤ 6) versus high-grade (Gleason score ≥ 7) cancers. Among men with prostate cancer (case-only analysis), we estimated associations of the standardized height and BMI weighted genetic risk scores with all-cause and prostate cancer-specific mortality using Cox proportional hazards regression, with age at diagnosis as the start date and age at death or final follow-up time-point as the exit date, with standard errors clustered by study (there was no evidence that the proportional hazards assumption was violated). We tested for heterogeneity in association of the genetic risk scores with localized versus advanced and low- versus high-grade prostate cancer risk using a multivariate logistic regression. We tested for heterogeneity in the association of the genetic risk scores and survival of patients with localized versus advanced and low versus high grade using the test proposed by Altman and Bland [33].

### Sensitivity analyses

We assessed the potential for pleiotropy, since it is possible that variants identified in the genome-wide scans are not specific for height or BMI and have effects on the prostate cancer outcomes independent of their effects on the exposures (height or BMI) [34]. If the no-effect modification assumption holds, similar instrumental variable estimates acquired using independent instruments would provide suggestive evidence against an influence of pleiotropic effects, as it is unlikely that they have shared pleiotropy [21, 35]. Therefore, as a sensitivity analysis we tested for evidence of heterogeneity across different SNPs for each of our baseline results which differed from the null. We generated two independent genetic instruments for BMI using (1) rs1558902 in *FTO*, the individual SNP with the largest effect size in the meta-analysis of GWASs for BMI [24] and (2) a weighted allelic score constructed from the remaining BMI-associated SNPs. We randomly split the height allele score into two independent weighted scores containing 89 and 90 SNPs (for details of the SNPs in each score see Supplementary Table 3). The height SNPs were in linkage equilibrium, and hence, these scores were statistically independent. We estimated the association of each instrument with prostate cancer and tested for heterogeneity [33].

The top eight principal components that reflect the population’s genetic structure were estimated and included as covariates in adjusted regression models to account for confounding by population stratification. We also report the associations of the genetic risk scores with survival additionally adjusted for PSA level, grade, and stage. We ran all statistical analyses in Stata version 13.1 (StataCorp LP, 2014, College Station, TX).

## Results

Our sample consisted of 20,848 cases and 20,214 controls of European genetic descent, with genotypic data from the iCOGs array that had passed quality control and was not included in the GIANT consortium used to generate the genetic risk scores (EPIC-Norfolk, CAPS, and SEARCH studies) (Table 1). The percentage of high-grade cancers reported varied between studies (3.6–35.0 %), as did the proportion of advanced stage cancers (3.5–64.5 %). The case-only survival analysis was based on 15,491 men, because 5,357 of the 20,848 men with prostate cancer did not have age at entry or exit in the dataset.

### Associations of genetic risk scores with measured height and BMI in ProtecT

Associations of the weighted genetic risk scores with height and BMI in the ProtecT sub-sample are shown in Table 2. The results with the unweighted score were similar, but less precise (results not shown). The genetic risk scores explained 6.31 and 1.46 % of the variability in height and BMI, respectively, consistent with previous studies [23, 24], which suggest that the genetic risk scores are strong instruments for the phenotypes.

**Table 2:** Association of weighted height and BMI genetic risk scores with measured height and weight in 907 controls in ProtecT [28]

	*n*	Mean difference	95 % CI		*r* ^2^ (%)	*F*-statistic
			Lower limit	Upper limit		
Height	907	0.26	0.20	0.33	6.31	67.6
BMI	901	0.12	0.06	0.19	1.46	13.6

To allow direct comparison of effect sizes, BMI and height phenotypic measurements and the genetic risk scores were normalized to mean zero and standard deviation one

### Associations of genetic risk scores with potential confounders in ProtecT

Taller men were more likely to have managerial jobs, have lower PSA levels, and have joined the ProtecT study at a younger age (Table 3), but there was little evidence that the height genetic risk score was associated with any of the confounders except benign hypertrophy of the prostate (all *p* values >0.05). Heavier men were more likely to have diabetes; be inactive; drink fewer than 3 drinks a week; be a nonsmoker; and have lower IGF-I levels (Table 3), but we found little evidence that the BMI genetic risk score was associated with any of the potential confounders (all *p* values >0.05).

**Table 3:** Odds ratio or change in continuous variable covariates per standard deviation change in either height and BMI (phenotypes) or genetic risk scores for height and BMI (instruments) in the ProtecT study cases [28]

	*n*	Observed phenotype^a^				Genetic risk scores^a^			
		Effect estimate	Confidence interval^b^		*p* value	Effect estimate	Confidence interval^b^		*p* value
			Lower	Upper			Lower	Upper	
Standardized height		Odds ratio^c^				Odds ratio^c^			
Binary variables									
Diabetes	726	0.91	0.64	1.30	0.62	0.94	0.70	1.25	0.66
Managerial occupation	818	1.21	1.06	1.40	0.006	0.91	0.79	1.04	0.17
Strenuous exercise	621	1.13	0.96	1.33	0.13	1.03	0.87	1.21	0.75
Moderate or strenuous exercise	621	1.15	0.96	1.37	0.12	1.01	0.85	1.20	0.90
≥3 drinks in the last week	820	1.13	0.98	1.30	0.09	1.05	0.91	1.22	0.47
Passive smoker	752	1.03	0.89	1.19	0.72	1.00	0.86	1.16	0.99
Ever smoker	780	1.10	0.95	1.27	0.21	1.08	0.93	1.25	0.33
Current smoker	552	1.09	0.89	1.35	0.40	1.21	0.97	1.51	0.08
Benign hypertrophy of the prostate	704	0.77	0.58	1.02	0.07	1.38	1.01	1.88	0.05

		Regression coefficient^c^				Regression coefficient^c^			
Continuous variables									
PSA (ng/ml)	828	−1.15	−2.01	−0.29	0.009	−0.31	−1.32	0.70	0.55
IGF-I (ng/ml)	718	1.80	−2.23	5.83	0.38	−2.53	−6.31	1.25	0.19
Age (years)	1,109	−0.53	−0.83	−0.24	<0.001	0.07	−0.23	0.38	0.64

Standardized BMI		Odds ratio^c^				Odds ratio^c^			
Binary variables									
Diabetes	724	1.90	1.45	2.48	<0.001	1.16	0.86	1.57	0.33
Managerial occupation	813	0.96	0.83	1.10	0.54	0.91	0.79	1.05	0.20
Strenuous exercise	617	0.91	0.77	1.08	0.28	1.05	0.89	1.23	0.57
Moderate or strenuous exercise	617	0.80	0.66	0.96	0.02	1.01	0.85	1.20	0.94
≥3 drinks in the last week	814	0.87	0.75	1.01	0.07	0.90	0.78	1.04	0.17
Passive smoker	748	1.12	0.97	1.30	0.13	0.97	0.84	1.12	0.65
Ever smoker	776	1.09	0.93	1.27	0.29	1.09	0.94	1.27	0.25
Current smoker	548	0.71	0.54	0.94	0.02	1.18	0.95	1.48	0.13
Benign hypertrophy of the prostate	700	0.92	0.69	1.23	0.56	0.94	0.71	1.25	0.66

		Regression coefficient^c^				Regression coefficient^c^			
Continuous variables									
PSA (ng/ml)	822	−0.25	−1.55	1.05	0.70	−0.21	−0.96	0.54	0.58
IGF-I (ng/ml)	714	−5.38	−9.12	−1.64	0.005	1.77	−1.97	5.51	0.35
Age (years)	1,101	−0.28	−0.60	0.04	0.08	−0.04	−0.33	0.25	0.79

^a^Observed phenotypes and genetic risk scores normalized to mean zero and standard deviation one

^b^Robust standard errors

^c^Odds ratio or change in continuous variable per standard deviation change in height and BMI (phenotype or genetic risk score)

### Association of the genetic risk scores and prostate cancer risk and mortality

Associations of the genetic risk scores for height and BMI with prostate cancer risk are shown in Table 4, with the study-specific estimates in Supplementary Figures 1–10. There was little consistent evidence that the genetic risk score for height was associated with prostate cancer, although there was weak evidence of an inverse association with advanced prostate cancer [OR, per standard deviation increase in height genetic score 0.96; 95 % CI 0.93, 0.99, *p* = 0.01; *p* heterogeneity, advanced vs. localized 0.05]. There was weak evidence that the genetic risk score for BMI was associated with a reduced prostate cancer risk (OR per standard deviation increase in BMI genetic score 0.98; 95 % CI 0.96, 1.00; *p* = 0.07), but little evidence of variation by stage or grade (*p* heterogeneity 0.64 and 0.13, respectively).

**Table 4:** Odds ratio of prostate cancer per one standard deviation change in height or BMI genetic score

	*n*	Unadjusted				Adjusted^a^				
		Odds ratio^*c*^	Confidence interval^b^		*p* value	Odds ratio^*c*^	Confidence interval^b^		*p* value	*p* heterogeneity^*d*^
			Lower	Upper			Lower	Upper		
*Height*										
Controls	20,214	1.00	–	–	–	1.00	–	–	–	–
All prostate cancers	20,848	0.96	0.91	1.01	0.12	0.99	0.97	1.01	0.23	
Localized prostate cancer	12,975	0.96	0.88	1.03	0.27	1.00	0.98	1.02	0.72	0.05
Advanced prostate cancer	4,325	0.90	0.83	0.98	0.02	0.96	0.93	0.99	0.01	
Low-grade prostate cancer	8,784	0.96	0.90	1.02	0.20	0.99	0.96	1.01	0.30	0.55
High-grade prostate cancer	8,230	0.97	0.92	1.02	0.26	1.00	0.98	1.02	0.85	
*BMI*										
Controls	20,214	1.00	–	–	–	1.00	–	–	–	–
All prostate cancers	20,848	0.98	0.96	1.01	0.15	0.98	0.96	1.00	0.07	
Localized prostate cancer	12,975	0.98	0.96	1.00	0.10	0.98	0.96	1.00	0.05	0.64
Advanced prostate cancer	4,325	1.01	0.97	1.05	0.69	1.01	0.97	1.05	0.62	
Low-grade prostate cancer	8,784	0.98	0.94	1.02	0.25	0.97	0.94	1.00	0.09	0.13
High-grade prostate cancer	8,230	1.00	0.97	1.02	0.69	1.00	0.98	1.01	0.65	

^a^Adjusted for the eight principal components of population stratification

^b^Based in robust standard errors to account for within-study clustering

^c^Change in odds ratio per standard deviation change in height and BMI genetic risk score (standardized to mean zero standard deviation one)

^d^Localized versus advanced, or high- versus low-grade using multivariate logistic regression

The height genetic risk score was associated with an increase in prostate cancer-specific mortality among men with low-grade disease (OR per standard deviation increase in the height score 1.13; 95 % CI 1.08, 1.20, *p* heterogeneity, low vs. high grade <0.001), but there was little evidence of associations with all-cause mortality (Table 5). The BMI genetic risk score was associated with higher all-cause mortality among low-grade disease (OR per standard deviation increase in the BMI score 1.08; 95 % CI 1.03, 1.14, *p* heterogeneity low vs. high grade = 0.03), but there was little evidence of associations with prostate cancer-specific mortality.

**Table 5:** Hazard ratio of all-cause and prostate cancer-specific mortality among men with prostate cancer per one standard change in height or BMI genetic score

	Number of participants	Number of failures	Years at risk (1000s)	Unadjusted				Adjusted^a^				
				Hazard ratio^*c*^	Confidence interval^*b*^		*p* value	Hazard ratio^*c*^	Confidence interval^*b*^		*p* value	*p* heterogeneity^*d*^
					Lower	Upper			Lower	Upper		
*All-cause mortality*												
Height												
All cases	14,649	3,591	105	1.02	0.97	1.08	0.47	1.00	0.96	1.04	0.88	
Localized	8,553	1,447	65	1.01	0.93	1.09	0.81	1.00	0.93	1.07	0.97	0.20
Advanced	3,435	1,332	25	1.08	0.98	1.18	0.11	1.07	0.99	1.14	0.07	
Low grade	5,684	905	43	1.04	0.97	1.11	0.32	1.02	0.95	1.09	0.57	0.80
High grade	5,892	1,365	36	1.02	0.97	1.08	0.36	1.01	0.96	1.06	0.71	
BMI												
All cases	14,649	3,591	105	1.02	0.99	1.05	0.18	1.02	0.99	1.05	0.23	
Localized	8,553	1,447	65	1.04	0.99	1.10	0.09	1.04	0.99	1.10	0.09	0.28
Advanced	3,435	1,332	25	1.01	0.98	1.04	0.50	1.01	0.98	1.05	0.59	
Low grade	5,684	905	43	1.09	1.04	1.15	0.001	1.08	1.03	1.14	0.002	0.03
High grade	5,892	1,365	36	1.00	0.96	1.05	0.89	1.00	0.95	1.05	0.98	
*Prostate cancer-specific mortality*												
Height												
All cases	14,649	1,483	105	1.02	0.98	1.06	0.44	1.00	0.97	1.04	0.87	
Localized	8,553	363	65	0.98	0.91	1.07	0.72	0.99	0.91	1.08	0.79	0.29
Advanced	3,435	745	25	1.05	1.00	1.10	0.06	1.04	1.00	1.09	0.07	
Low grade	5,684	188	43	1.13	1.06	1.21	<0.001	1.13	1.08	1.20	<0.001	<0.001
High grade	5,892	678	36	0.97	0.93	1.02	0.20	0.97	0.93	1.01	0.19	
BMI												
All cases	14,649	1,483	105	0.99	0.96	1.03	0.76	1.00	0.96	1.04	0.94	
Localized	8,553	363	65	0.95	0.88	1.03	0.22	0.95	0.87	1.05	0.31	0.09
Advanced	3,435	745	25	1.04	0.98	1.10	0.18	1.05	0.99	1.10	0.11	
Low grade	5,684	188	43	0.95	0.89	1.01	0.08	0.95	0.88	1.01	0.12	0.03
High grade	5,892	678	36	1.05	0.99	1.11	0.12	1.05	0.98	1.13	0.14	

^a^Adjusted for the first eight principal components of population stratification

^b^Based in robust standard errors to account for within-study clustering

^c^Change in hazard ratio per standard deviation change in height and BMI genetic risk score (standardized to mean zero standard deviation one)

^d^Localized versus advanced, or high- versus low-grade using Bland–Altman tests

### Sensitivity analysis

#### Prostate cancer risk

**Fig. 1:**
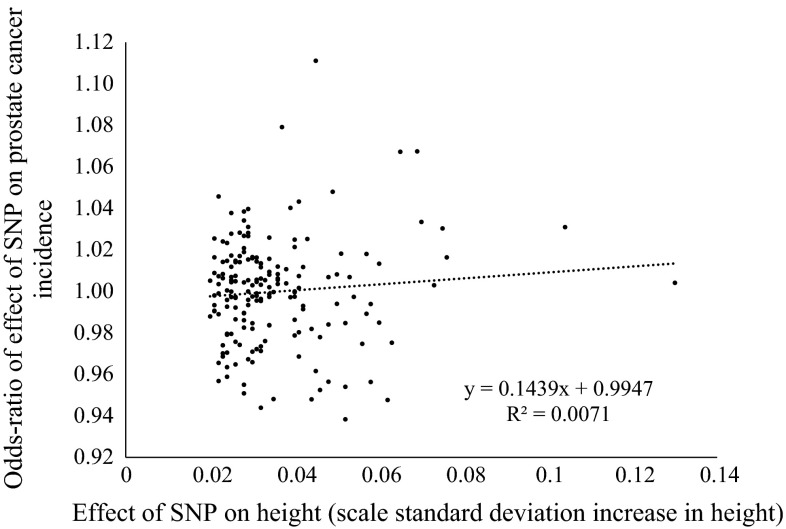
Scatter plot of effects of SNPs on prostate cancer risk by their effects on height

**Fig. 2:**
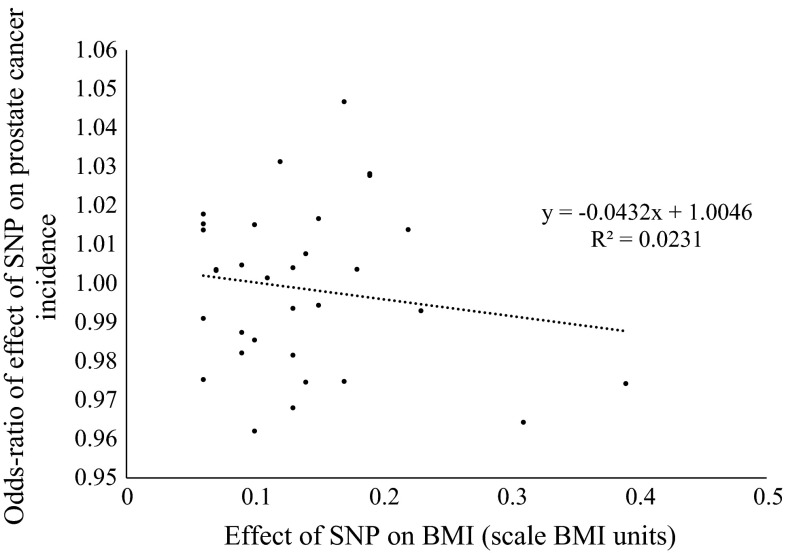
Scatter plot of effects of SNPs on prostate cancer risk by their effects on BMI

There was little evidence that men with height variants with larger effects on the height phenotype were more or less likely to be diagnosed with prostate cancer (*r*^2^ = 0.0071) (Fig. 1; see Supplementary Table 4 for associations of each of the height variants with prostate cancer risk). There was some evidence that BMI variants with the largest effect on BMI were most strongly inversely associated with prostate cancer (*r*^2^ = 0.0231) (Fig. 2; Supplementary Table 5 for associations of each of the BMI variants with prostate cancer risk). We found little evidence of heterogeneity in the effect of BMI proxied by independent instruments based on independent genetic scores made up of different sets of SNPs. Individuals with more BMI increasing *FTO* alleles were less likely to be diagnosed with prostate cancer (OR per BMI increasing allele rs1558902-A 0.97; 95 % CI 0.94, 1.01, *p* = 0.10). In line with this, the allele score based on the remaining 31 BMI SNPs was also inversely associated with prostate cancer (OR per standard deviation increase in BMI genetic score excluding FTO 0.99; 95 % CI 0.97, 1.01, *p* = 0.33; *p* value for heterogeneity between the two independent instruments = 0.38).

#### All-cause mortality

There was little evidence that the two sets of independent height or BMI allele scores were associated with an increased risk of all-cause mortality in men diagnosed with prostate cancer (see Supplementary Table 10 for associations of all 179 height SNPs and all-cause mortality and Supplementary Table 7 for associations of each of the 32 BMI SNPs with all-cause mortality and prostate cancer-specific mortality).

#### Prostate cancer-specific mortality

Both sets of independent height allele scores were associated with an increased risk of prostate cancer-specific mortality in men diagnosed with low-grade prostate cancer (hazard ratio per one standard deviation increase in the first height allele score 1.10; 95 % CI 1.03, 1.19, *p* = 0.008; and in the second height allele score 1.09; 95 % CI 1.05, 1.13, *p* < 0.001; *p* value for heterogeneity = 0.86; see Supplementary Table 8 for the association of prostate cancer-specific mortality and each of the 179 height SNPs). None of the BMI independent instruments or individual SNPs were associated with prostate cancer-specific mortality (Supplementary Table 9). Further adjusting the associations of the genetic risk scores and survival for PSA level, grade, and stage made no substantial differences to the results (Supplementary Table 10).

## Discussion

We found weak evidence that genetically elevated BMI was associated with a reduced risk of prostate cancer, but that genetically elevated height was not associated with prostate cancer risk. The height and BMI allele scores were positively associated with prostate cancer-specific and all-cause mortality, respectively, but only among men with low-grade disease (*p* heterogeneity, low- vs. high-grade prostate cancer <0.05).

Although evidence for these associations was relatively weak, the inverse relationship of BMI with prostate cancer risk is in line with both observational data [8] and our previous genetic study [15]. The latter report showed an inverse relationship of a single obesity-related SNP (*FTO* rs9939609) with overall- and low-grade prostate cancer in ProtecT, a much smaller population-based sample of 1,550 screen-detected prostate cancers and 1,815 controls [15]. We found inverse associations of a related SNP in *FTO* (rs1558902, which is in linkage disequilibrium with rs9939609 at *r*^2^ = 0.90) with overall and low-grade prostate cancer risk (individuals with more BMI increasing alleles had a reduced risk of all prostate cancer and low-grade prostate cancer, respectively (OR 0.97; 95 % CI 0.94, 1.00; *p* = 0.10, and OR 0.95; 95 % CI 0.90, 1.00; *p* = 0.06). A previous study that created genetic risk scores for BMI using 24 of the 32 SNPs from the BMI GWAS [36] observed ORs for the BMI risk scores of 1.00 (95 % CI 0.97, 1.04; *p* = 0.94) for the unweighted score and 1.07 (95 % CI 0.91, 1.25; *p* = 0.41) for the weighted score, but the effect estimates were imprecise as the study only included 871 cases and 906 controls.

To determine whether our findings reflect clinically important differences in disease risk, we rescaled the results to report the effects of one standard deviation changes in height and one kg/m^2^ increases in BMI using the coefficients for the effects of the SNPs on height and BMI reported by Lango Allen et al. [23] and Speliotes et al. [24], respectively. Our results imply that a one standard deviation increase in height was associated with a 49 % (95 % CI 26 %, 76 %, *p* < 0.001) increase in prostate cancer mortality among those with low-grade disease, assuming that the height allele score explains 10 % of the variation in height [23]. A 1 kg/m^2^ increase in BMI was associated with a 4 % (95 % CI 0 %, 8 %, *p* = 0.07) reduced risk of any prostate cancer diagnosis (assuming that the BMI allele score explains 1.45 % of the variation in BMI [24] and a standard deviation of BMI of 3.5 kg/m^2^). The risk of all-cause mortality was increased by 21 % (95 % CI 7 %, 37 %) per kg/m^2^ increase in BMI among men with low-grade disease.

Our finding that genetic variation in height was not associated with an increase in prostate cancer risk is in contrast to the majority of the observational literature [6]. Indeed, we found weak evidence of an inverse association of genetic variation in height with advanced prostate cancer. However, there is some evidence of reporting bias in the previous literature; 12 of 30 prospective studies that reported effects of height on prostate cancer only in the body of the manuscript, and which were not highlighted in the title or abstract, were null (pooled OR 1.01; 0.95–1.07; *I*^2^ 0 %) [6]. The absence of a positive association of genetic variation in height with prostate cancer risk in the current study may reflect that there is no real effect of height on prostate cancer risk or that it is the environmental (especially early-life factors [37, 38]) and not genetic component of height variation that explains its positive link in some studies with incident prostate cancer [16–18, 39]. Alternatively, if height is associated with very early case-fatality in men with prostate cancer, then this will remove cases from the pool available for case–control studies and could theoretically lead to selection bias causing null findings. However, prostate cancer is not generally so rapidly fatal as to preclude significant numbers of men from being included in case–control studies.

The height and BMI allele scores were positively associated, respectively, with prostate cancer-specific and all-cause mortality, but only strongly among men with low-grade disease. The positive association of BMI and height with mortality among men with prostate cancer is in line with earlier studies [9, 11, 40], although previous findings for height have been inconsistent [41, 42], and one study observed that taller men with prostate cancer had improved survival [43]. Our data suggest that only height is associated with prostate cancer-specific, rather than all-cause, mortality, and that BMI causes a broader range of deaths among men with prostate cancer.

The difference in the magnitude of effect estimates by grade does not appear to have been reported in the past and could simply be a chance finding. However, the p values for heterogeneity for the association of the BMI allele score with all-cause mortality and the height allele score with prostate cancer-specific mortality by grade were 0.03 and <0.001, respectively. The findings could, therefore, reflect differing determinants of progression depending on grade. This highlights the potential for modifying BMI in people with low-grade disease; however, it is likely that the genetic contribution to height explains the association of the height genetic score with prostate cancer progression. Such genetic influences could include effects on insulin-like growth factors (IGFs), which have been associated with progression of prostate cancer [44].

The strengths of the study include (1) the robust instruments developed from previous GWAS that explained a reasonable proportion of the variance in the phenotypes of interest, (2) the large sample size, and (3) the potential confounders which were associated with measured height and BMI within the ProtecT study were not associated with the genetic risk scores for height and BMI. The final point suggests that association of genetic risk scores with prostate cancer outcomes is unlikely to be explained by confounders. Evidence from genetic variation is less likely to suffer from biases that affect conventional observational studies. The generalizability of our findings is supported by broadly consistent results across the 22 studies. There are limitations to our Mendelian randomization approach, and our results could be explained by bias or confounding. For example, we used combinations of genome-wide genetic variants to proxy BMI and height, but these variants may not be specific for BMI or height and may influence prostate cancer through biological pathways other than through the phenotypes that they are acting as proxies for (genetic confounding or pleiotropy). This is plausible since even single SNPs can exert pleiotropic effects across a range of different variables [45]; for example, many BMI-associated SNPs are present at quite low levels of significance in a GWAS of c-reactive protein (CRP) [34]. However, we found similar results when we used two independent instruments for each phenotype, suggesting these results may not be due pleiotropy of a single SNP. We assumed a similar qualitative effect of the SNPs in our sample as the GIANT consortium, which is highly plausible but may not be true. We found little evidence that the genetic risk scores were associated with baseline covariates in the ProtecT study, and this is consistent with findings from the broader literature [46–49].

A reduced risk of prostate cancer associated with BMI is biologically plausible, with proposed mechanisms including the increase in estrogens (aromatase inhibitors) secondary to adiposity. However, we cannot rule out detection bias [50] arising from delayed diagnosis and more advanced stage at diagnosis in obese men; this may arise due to lower accuracy of digital rectal examination in obese men or lower PSA values caused by obesity-related hemodilution [8, 9].

In conclusion, our genetic data provide some evidence (albeit weak) that an elevated BMI may protect against prostate cancer risk or reduce the likelihood of it being detected (in particular, low-grade cancer), but may increase the likelihood of death in men with low-grade prostate cancer. These observations support epidemiological findings that obesity protects against a diagnosis of localized prostate cancer but increases prostate cancer mortality [8]. Previously observed positive associations of height with prostate cancer risk may reflect the environmental determinants of height. In contrast, observed positive associations of height with prostate cancer mortality may reflect the genetic determinants of height or of height determining phenotypes (e.g., IGF [39]). The findings for mortality that were only observed among men with low-grade disease are novel, and potentially clinically important, but do require replication.

### Electronic supplementary material

Supplementary material 1 (DOCX 247 kb)

## The PRACTICAL CONSORTIUM (in addition to those named in the author list)

Information on the consortium can be found at http://practical.ccge.medschl.cam.ac.uk/.

Additional members from the consortium are: Margaret Cook^1^, Angela Morgan^2^, Artitaya Lophatananon^3,4^, Cyril Fisher^2^, Daniel Leongamornlert^2^, Edward J. Saunders^2^, Emma J. Sawyer^2^, Koveela Govindasami^2^, Malgorzata Tymrakiewicz^2^, Michelle Guy^2^, Naomi Livni^2^, Rosemary Wilkinson^2^, Sara Jugurnauth-Little^2^, Steve Hazel^2^, Tokhir Dadaev^2^, Melissa C. Southey^5^, Liesel M. Fitzgerald^6^, John Pedersen^7^, John Hopper^8^, Ami Karlsson^9^, Carin Cavalli-Bjoerkman^9^, Jan-Erik Johansson^9^, Jan Adolfson^9^, Markus Aly^9,10^, Michael Broms^9^, Paer Stattin^9^, Brian E. Henderson^11^, Fredrick Schumacher^11^, Anssi Auvinen^12^, Kimmo Taari^13^, Liisa Maeaettaenen^14^, Paula Kujala^15^, Teemu Murtola^16,17^, Teuvo LJ Tammela^17^, Tiina Wahlfors^18^, Andreas Roder^19^, Peter Iversen^19^, Peter Klarskov^20^, Sune F. Nielsen^21,22^, Tim J. Key^23^, Hans Wallinder^24^, Sven Gustafsson^24^, Jenny L. Donovan^25^, Freddie Hamdy^26^, Angela Cox^27^, Anne George^28^, Athene Lane^28^, Gemma Marsden^26^, Michael Davis^25^, Paul Brown^25^, Paul Pharoah^29^, Lisa B. Signorello^31,30^, Wei Zheng^32^, Shannon K. McDonnell^33^, Daniel J. Schaid^33^, Liang Wang^33^, Lori Tillmans^33^, Shaun Riska^33^, Thomas Schnoeller^34^, Kathleen Herkommer^35^, Manuel Luedeke^34^, Walther Vogel^36^, Dominika Wokozorczyk^37^, Jan Lubiski^37^, Wojciech Kluzniak^37^, Katja Butterbach^38^, Christa Stegmaier^39^, Bernd Holleczek^39^, Babu Zachariah^40^, Hui-Yi Lim^41^, Hyun Park^40^, James Haley^40^, Julio Pow-Sang^40^, Maria Rincon^40^, Selina Radlein^40^, Thomas Sellers^40^, Chavdar Slavov^42^, Aleksandrina Vlahova^43^, Atanaska Mitkova^44^, Darina Kachakova^44^, Elenko Popov^42^, Svetlana Christova^43^, Tihomir Dikov^43^, Vanio Mitev^44^, Allison Eckert^45^, Amanda Spurdle^46^, Angus Collins^45^, Glenn Wood^45^, Greg Malone^45^, Judith A. Clements^45^, Kris Kerr^45^, Megan Turner^45^, Pamela Saunders^45^, Peter Heathcote^45^, Srilakshmi Srinivasan^45^, Leire Moya^45^, Trina Yeadon^45^, Australian Prostate Cancer BioResource^45^, Joana Santos^47^, Carmen Jerónimo^47^, Paula Paulo^47^, Pedro Pinto^47^, Rui Henrique^47^, Sofia Maia^47^, Agnieszka Michael^48^, Andrzej Kierzek^48^, Huihai Wu^48^

^1^Centre for Cancer Genetic Epidemiology, Department of Public Health and Primary Care, University of Cambridge, Strangeways Laboratory, Worts Causeway, Cambridge CB1 8RN, UK, ^2^The Institute of Cancer Research, Sutton, UK, ^3^Institute of Population Health, University of Manchester, Manchester, UK, ^4^Warwick Medical School, University of Warwick, Coventry, UK, ^5^Genetic Epidemiology Laboratory, Department of Pathology, The University of Melbourne, Grattan Street, Parkville, Victoria 3010, Australia, ^6^Cancer Epidemiology Centre, The Cancer Council Victoria, 615 St Kilda Road, Melbourne, Victoria, Australia, ^7^ Tissupath Pty Ltd., Melbourne,Victoria 3122, Australia, ^8^Centre for Epidemiology and Biostatistics, Melbourne School of Population and Global Health, The University of Melbourne, Victoria, Australia, ^9^Department of Medical Epidemiology and Biostatistics, Karolinska Institute, Stockholm, Sweden, ^10^Department of Clinical Sciences at Danderyds Hospital, Stockholm, Sweden, ^11^Department of Preventive Medicine, Keck School of Medicine, University of Southern California/Norris Comprehensive Cancer Center, Los Angeles, California, USA, ^12^Department of Epidemiology, School of Health Sciences, University of Tampere, Tampere, Finland, ^13^Department of Urology, Helsinki University Central Hospital and University of Helsinki, Helsinki, Finland, ^14^Finnish Cancer Registry, Helsinki, Finland, ^15^Fimlab Laboratories, Tampere University Hospital, Tampere, Finland, ^16^School of Medicine, University of Tampere, Tampere, Finland, ^17^Department of Urology, Tampere University Hospital and Medical School, University of Tampere, Finland, ^18^BioMediTech, University of Tampere and FimLab Laboratories, Tampere, Finland, ^19^Copenhagen Prostate Cancer Center, Department of Urology, Rigshospitalet, Copenhagen University Hospital, Tagensvej 20, 7521, DK-2200 Copenhagen, Denmark, ^20^Department of Urology, Herlev Hospital, Copenhagen University Hospital, Herlev Ringvej 75, DK-230 Herlev, Denmark, ^21^Department of Clinical Biochemistry, Herlev Hospital, Copenhagen University Hospital, Herlev Ringvej 75, DK-230 Herlev, Denmark, ^22^Faculty of Health and Medical Sciences, University of Copenhagen, ^23^Cancer Epidemiology Unit, Nuffield Department of Clinical Medicine, University of Oxford, Oxford, UK, ^24^Department of Epidemiology and Biostatistics, School of Public Health, Imperial College, London, UK, ^25^School of Social and Community Medicine, University of Bristol, Canynge Hall, 39 Whatley Road, Bristol, BS8 2PS, UK, ^26^Nuffield Department of Surgical Sciences, University of Oxford, Oxford, UK, Faculty of Medical Science, University of Oxford, John Radcliffe Hospital, Oxford, UK, ^27^CR-UK/YCR Sheffield Cancer Research Centre, University of Sheffield, Sheffield, UK, ^28^University of Cambridge, Department of Oncology, Box 279, Addenbrooke’s Hospital, Hills Road Cambridge CB2 0QQ, UK, ^29^Centre for Cancer Genetic Epidemiology, Department of Oncology, University of Cambridge, Strangeways Laboratory, Worts Causeway, Cambridge, UK, ^30^International Epidemiology Institute, 1555 Research Blvd., Suite 550, Rockville, MD 20850, USA, ^31^Department of Epidemiology, Harvard School of Public Health, 677 Huntington Avenue, Boston, MA 02115, USA, ^32^Division of Epidemiology, Department of Medicine, Vanderbilt University Medical Center, 2525 West End Avenue, Suite 800, Nashville, TN 37232 USA, ^33^Mayo Clinic, Rochester, Minnesota, USA, ^34^Department of Urology, University Hospital Ulm, Germany, ^35^Department of Urology, Klinikum rechts der Isar der Technischen Universitaet Muenchen, Munich, Germany, ^36^Institute of Human Genetics, University Hospital Ulm, Germany, ^37^International Hereditary Cancer Center, Department of Genetics and Pathology, Pomeranian Medical University, Szczecin, Poland, ^38^Division of Clinical Epidemiology and Aging Research, German Cancer Research Center (DKFZ), 69120 Heidelberg, Germany, ^39^Saarland Cancer Registry, 66119 Saarbruecken, Germany, ^40^Department of Cancer Epidemiology, Moffitt Cancer Center, 12902 Magnolia Drive, Tampa, FL 33612, USA, ^41^Biostatistics Program, Moffitt Cancer Center, 12902 Magnolia Drive, Tampa, FL 33612, USA, ^42^Department of Urology and Alexandrovska University Hospital, Medical University, Sofia, Bulgaria, ^43^Department of General and Clinical Pathology, Medical University, Sofia, Bulgaria, ^44^Department of Medical Chemistry and Biochemistry, Molecular Medicine Center, Medical University, Sofia, 2 Zdrave Str., 1431 Sofia, Bulgaria, ^45^Australian Prostate Cancer Research Centre-Qld, Institute of Health and Biomedical Innovation and School of Biomedical Science, Queensland University of Technology, Brisbane, Australia, ^46^Molecular Cancer Epidemiology Laboratory, Queensland Institute of Medical Research, Brisbane, Australia, ^47^Department of Genetics, Portuguese Oncology Institute, Porto, Portugal, ^48^The University of Surrey, Guildford, Surrey, GU2 7XH, UK.
