# Supplementary material for: The effects of height and BMI on prostate cancer incidence and mortality: a Mendelian randomization study in 20,848 cases and 20,214 controls from the PRACTICAL consortium
Source: Cancer Causes Control. 2015 Sep 19;26(11):1603–16. doi: 10.1007/s10552-015-0654-9 (PMC4596899; doi:10.1007/s10552-015-0654-9)
Supplement: Supplementary file 1 — Supplementary material 1 (DOCX 247 kb) [file 10552_2015_654_MOESM1_ESM.docx]

**The effects of height and BMI on prostate cancer incidence and mortality: a Mendelian randomization study in 20,848 cases and 20,214 controls from the PRACTICAL consortium**

**Supplementary Materials**

**Index**

**Supplementary Table 1: Height alleles, frequency and weight assigned in iCOGS (n= 41,062).**

**Supplementary Table 2: BMI alleles, frequency and weight assigned in iCOGS (n= 41,062).**

**Supplementary Table 3: Height SNPs included in the independent split allele scores**

**Supplementary Table 4: Association of individual height SNPs with prostate cancer outcomes. Odds-ratios indicate the effect of one extra height increasing allele.**

**Supplementary Table 5: Association of 32 individual BMI SNPs with prostate cancer risk. Odds-ratios indicate the effect of one extra BMI increasing allele.**

**Supplementary Table 6: Association of 179 individual height SNPs with all-cause mortality after prostate cancer diagnosis. Hazard ratios indicate the effect of one extra height increasing allele.**

**Supplementary Table 7: Association of 32 individual BMI SNPs with all-cause mortality after prostate cancer diagnosis. Hazard ratios indicate the effect of one extra height increasing allele.**

**Supplementary Table 8: Association of 179 individual height SNPs with prostate cancer specific mortality after prostate cancer diagnosis. Hazard ratios indicate the effect of one extra height increasing.**

**Supplementary Table 9: Association of 32 individual BMI SNPs with prostate cancer specific mortality after prostate cancer diagnosis. Hazard ratios indicate the effect of one extra height increasing allele.**

**Supplementary Table 10: Hazard ratio of all-cause and prostate cancer specific mortality of prostate cancer patients per one standard change in height or BMI genetic score. Additionally adjusted for PSA level, stage and grade.**

**Supplementary Figure 1: Association of a 1 standard deviation change in the weighted height genetic risk score with prostate cancer by study.**

**Supplementary Figure 2: Association of 1 standard deviation change in the weighted height genetic risk score with localised prostate cancer by study.**

**Supplementary Figure 3: Association of a 1 standard deviation change in the weighted height genetic risk score with advanced prostate cancer by study.**

**Supplementary Figure 4: Association of a 1 standard deviation change in the weighted height genetic risk score with low grade prostate cancer by study.**

**Supplementary Figure 5: Association of a 1 standard deviation change in the weighted height genetic risk score with high grade prostate cancer by study.**

**Supplementary Figure 6: Association of a 1 standard deviation change in the weighted BMI genetic risk score with prostate cancer by study.**

**Supplementary Figure 7: Association of a 1 standard deviation change in the weighted BMI genetic risk score with localised prostate cancer by study.**

**Supplementary Figure 8: Association of a 1 standard deviation change in the weighted BMI genetic risk score with advanced prostate cancer by study.**

**Supplementary Figure 9: Association of a 1 standard deviation change in the weighted BMI genetic risk score with low grade prostate cancer by study.**

**Supplementary Figure 10: Association of a 1 standard deviation change in the weighted BMI genetic risk score with high grade prostate cancer by study.**

**Supplementary Table 1: Height alleles, frequency and weight assigned in iCOGS (n= 41,062).**

|  |  |  |  |  | **Frequency of** |  |
| --- | --- | --- | --- | --- | --- | --- |
| **SNP** | **Chr** | **Position (bp)** | **Effect allele** | **Other allele** | **effect allele** | **Weight** |
| rs425277 | 1 | 2059032 | T | C | 0.288 | 0.022 |
| rs2284746 | 1 | 17179262 | G | C | 0.496 | 0.04 |
| rs1738475 | 1 | 23409478 | C | G | 0.603 | 0.025 |
| rs4601530 | 1 | 24916698 | C | T | 0.715 | 0.028 |
| rs7532866 | 1 | 26614131 | A | G | 0.660 | 0.021 |
| rs2154319 | 1 | 41518357 | C | T | 0.212 | 0.03 |
| rs17391694 | 1 | 78396214 | T | C | 0.115 | 0.042 |
| rs6699417 | 1 | 88896031 | T | C | 0.600 | 0.021 |
| rs10874746 | 1 | 93096559 | C | T | 0.648 | 0.024 |
| rs9428104 | 1 | 118657110 | G | A | 0.754 | 0.041 |
| rs11205277 | 1 | 148159496 | G | A | 0.424 | 0.046 |
| rs17346452 | 1 | 170319910 | C | T | 0.271 | 0.04 |
| rs1325598 | 1 | 175058872 | G | A | 0.548 | 0.022 |
| rs1046934 | 1 | 182290152 | C | A | 0.351 | 0.044 |
| rs10863936 | 1 | 210304421 | G | A | 0.468 | 0.021 |
| rs6684205 | 1 | 216676325 | G | A | 0.289 | 0.028 |
| rs11118346 | 1 | 217810342 | C | T | 0.544 | 0.025 |
| rs10799445 | 1 | 225978506 | A | C | 0.766 | 0.032 |
| rs4665736 | 2 | 25041103 | T | C | 0.537 | 0.029 |
| rs6714546 | 2 | 33214929 | G | A | 0.719 | 0.026 |
| rs17511102 | 2 | 37814117 | T | A | 0.069 | 0.06 |
| rs2341459 | 2 | 44621706 | T | C | 0.269 | 0.025 |
| rs12474201 | 2 | 46774789 | A | G | 0.357 | 0.028 |
| rs3791675 | 2 | 55964813 | C | T | 0.764 | 0.053 |
| rs11684404 | 2 | 88705737 | C | T | 0.347 | 0.028 |
| rs7567288 | 2 | 134151294 | C | T | 0.211 | 0.032 |
| rs7567851 | 2 | 178392966 | C | G | 0.078 | 0.037 |
| rs1351164 | 2 | 217980143 | T | C | 0.796 | 0.034 |
| rs12470505 | 2 | 219616613 | T | G | 0.904 | 0.041 |
| rs2629046 | 2 | 224755988 | T | C | 0.552 | 0.024 |
| rs2580816 | 2 | 232506210 | C | T | 0.784 | 0.045 |
| rs12694997 | 2 | 241911659 | G | A | 0.776 | 0.024 |
| rs2597513 | 3 | 13530836 | C | T | 0.106 | 0.036 |
| rs13088462 | 3 | 51046753 | C | T | 0.056 | 0.052 |
| rs2336725 | 3 | 53093779 | C | T | 0.440 | 0.027 |
| rs9835332 | 3 | 56642722 | G | C | 0.530 | 0.026 |
| rs17806888 | 3 | 67499012 | T | C | 0.884 | 0.036 |
| rs9863706 | 3 | 72520103 | C | T | 0.783 | 0.031 |
| rs6439167 | 3 | 130533446 | C | T | 0.789 | 0.034 |
| rs9844666 | 3 | 137456906 | G | A | 0.759 | 0.024 |
| rs724016 | 3 | 142588260 | G | A | 0.454 | 0.07 |
| rs572169 | 3 | 173648421 | T | C | 0.315 | 0.033 |
| rs720390 | 3 | 187031377 | A | G | 0.347 | 0.029 |
| rs2247341 | 4 | 1671115 | A | G | 0.357 | 0.025 |
| rs6449353 | 4 | 17642586 | T | C | 0.853 | 0.075 |
| rs17081935 | 4 | 57518233 | T | C | 0.198 | 0.03 |
| rs7697556 | 4 | 73734177 | T | C | 0.461 | 0.028 |
| rs788867 | 4 | 82369030 | G | T | 0.295 | 0.043 |
| rs10010325 | 4 | 106325802 | A | C | 0.491 | 0.024 |
| rs7689420 | 4 | 145787802 | C | T | 0.834 | 0.073 |
| rs955748 | 4 | 184452669 | G | A | 0.760 | 0.023 |
| rs1173727 | 5 | 32866278 | T | C | 0.410 | 0.034 |
| rs11958779 | 5 | 55037656 | G | A | 0.311 | 0.027 |
| rs10037512 | 5 | 88390431 | T | C | 0.538 | 0.032 |
| rs13177718 | 5 | 108141243 | C | T | 0.929 | 0.04 |
| rs1582931 | 5 | 122685098 | G | A | 0.513 | 0.023 |
| rs274546 | 5 | 131727766 | G | A | 0.606 | 0.029 |
| rs526896 | 5 | 134384604 | T | G | 0.723 | 0.03 |
| rs4282339 | 5 | 168188818 | G | A | 0.792 | 0.036 |
| rs12153391 | 5 | 171136043 | C | A | 0.751 | 0.03 |
| rs889014 | 5 | 172916720 | C | T | 0.646 | 0.03 |
| rs422421 | 5 | 176449932 | C | T | 0.790 | 0.031 |
| rs6879260 | 5 | 179663620 | C | T | 0.632 | 0.022 |
| rs3812163 | 6 | 7670759 | T | A | 0.458 | 0.036 |
| rs1047014 | 6 | 19949472 | C | T | 0.210 | 0.032 |
| rs806794 | 6 | 26308656 | A | G | 0.697 | 0.052 |
| rs3129109 | 6 | 29192211 | C | T | 0.606 | 0.032 |
| rs114684762 | 6 | 31380529 | A | G | 0.464 | 0.04 |
| rs115961701 | 6 | 32663999 | G | C | 0.492 | 0.029 |
| rs2780226 | 6 | 34307070 | C | T | 0.083 | 0.076 |
| rs6457821 | 6 | 35510783 | C | A | 0.984 | 0.104 |
| rs9472414 | 6 | 45054484 | T | A | 0.775 | 0.026 |
| rs9360921 | 6 | 76322362 | G | T | 0.107 | 0.042 |
| rs310405 | 6 | 81857081 | A | G | 0.545 | 0.026 |
| rs7759938 | 6 | 105485647 | T | C | 0.673 | 0.02 |
| rs1046943 | 6 | 109890634 | G | A | 0.418 | 0.024 |
| rs961764 | 6 | 117628849 | C | G | 0.416 | 0.034 |
| rs1490384 | 6 | 126892853 | C | T | 0.500 | 0.04 |
| rs6569648 | 6 | 130390812 | C | T | 0.229 | 0.048 |
| rs7763064 | 6 | 142838982 | G | A | 0.720 | 0.034 |
| rs543650 | 6 | 152152636 | G | T | 0.593 | 0.048 |
| rs9456307 | 6 | 158849430 | T | A | 0.957 | 0.045 |
| rs798489 | 7 | 2768329 | C | T | 0.739 | 0.048 |
| rs4470914 | 7 | 19583047 | T | C | 0.172 | 0.029 |
| rs12534093 | 7 | 23469499 | T | A | 0.775 | 0.034 |
| rs1708299 | 7 | 28156471 | A | G | 0.303 | 0.04 |
| rs6959212 | 7 | 38094851 | C | T | 0.671 | 0.024 |
| rs42235 | 7 | 92086012 | T | C | 0.306 | 0.057 |
| rs822552 | 7 | 148281567 | G | C | 0.261 | 0.025 |
| rs77505230 | 7 | 150517022 | G | C | 0.317 | 0.031 |
| rs1013209 | 8 | 24172249 | C | T | 0.736 | 0.025 |
| rs7460090 | 8 | 57356717 | T | C | 0.885 | 0.058 |
| rs6473015 | 8 | 78341040 | C | A | 0.279 | 0.029 |
| rs6470764 | 8 | 130794847 | C | T | 0.792 | 0.05 |
| rs12680655 | 8 | 135706519 | C | G | 0.600 | 0.028 |
| rs7864648 | 9 | 16358732 | T | G | 0.321 | 0.022 |
| rs11144688 | 9 | 77732106 | G | A | 0.904 | 0.049 |
| rs7853377 | 9 | 85742025 | G | A | 0.223 | 0.024 |
| rs8181166 | 9 | 88306448 | C | G | 0.526 | 0.026 |
| rs2778031 | 9 | 90025546 | T | C | 0.254 | 0.031 |
| rs9969804 | 9 | 94468941 | A | C | 0.410 | 0.03 |
| rs1257763 | 9 | 95933766 | A | G | 0.036 | 0.069 |
| rs473902 | 9 | 97296056 | T | G | 0.931 | 0.065 |
| rs7027110 | 9 | 108638867 | A | G | 0.224 | 0.031 |
| rs1468758 | 9 | 112846903 | C | T | 0.733 | 0.026 |
| rs751543 | 9 | 118162163 | T | C | 0.676 | 0.026 |
| rs7466269 | 9 | 132453905 | A | G | 0.619 | 0.032 |
| rs7849585 | 9 | 138251691 | T | G | 0.316 | 0.029 |
| rs7909670 | 10 | 12958770 | C | T | 0.550 | 0.021 |
| rs2145998 | 10 | 80791702 | T | A | 0.523 | 0.026 |
| rs11599750 | 10 | 101795432 | C | T | 0.620 | 0.028 |
| rs2237886 | 11 | 2767307 | T | C | 0.100 | 0.046 |
| rs7926971 | 11 | 12654616 | G | A | 0.422 | 0.023 |
| rs1330 | 11 | 17272605 | T | C | 0.350 | 0.022 |
| rs79890777 | 11 | 48098280 | G | A | 0.278 | 0.027 |
| rs1814175 | 11 | 49515748 | T | C | 0.428 | 0.022 |
| rs3782089 | 11 | 65093395 | C | T | 0.930 | 0.058 |
| rs7112925 | 11 | 66582736 | C | T | 0.660 | 0.023 |
| rs634552 | 11 | 74959700 | T | G | 0.187 | 0.039 |
| rs494459 | 11 | 118079885 | T | C | 0.405 | 0.02 |
| rs654723 | 11 | 128091365 | A | C | 0.616 | 0.025 |
| rs2856321 | 12 | 11747040 | G | A | 0.365 | 0.029 |
| rs10770705 | 12 | 20748734 | A | C | 0.333 | 0.033 |
| rs2638953 | 12 | 28425682 | C | G | 0.685 | 0.032 |
| rs2066807 | 12 | 55026949 | G | C | 0.066 | 0.054 |
| rs1351394 | 12 | 64638093 | T | C | 0.499 | 0.06 |
| rs10748128 | 12 | 68113925 | T | G | 0.358 | 0.038 |
| rs11107116 | 12 | 92502635 | T | G | 0.221 | 0.052 |
| rs7971536 | 12 | 100897919 | T | A | 0.518 | 0.028 |
| rs11830103 | 12 | 122389499 | G | A | 0.216 | 0.035 |
| rs7332115 | 13 | 32045548 | G | T | 0.380 | 0.023 |
| rs3118905 | 13 | 50003335 | G | A | 0.713 | 0.056 |
| rs7319045 | 13 | 90822575 | A | G | 0.398 | 0.025 |
| rs1950500 | 14 | 23900690 | T | C | 0.309 | 0.034 |
| rs2093210 | 14 | 60027032 | C | T | 0.384 | 0.032 |
| rs1570106 | 14 | 67882868 | C | T | 0.803 | 0.026 |
| rs862034 | 14 | 74060499 | G | A | 0.641 | 0.028 |
| rs7155279 | 14 | 91555634 | G | T | 0.639 | 0.024 |
| rs16964211 | 15 | 49317787 | G | A | 0.946 | 0.05 |
| rs7178424 | 15 | 60167551 | C | T | 0.528 | 0.021 |
| rs10152591 | 15 | 67835211 | A | C | 0.906 | 0.041 |
| rs12902421 | 15 | 69948457 | C | T | 0.009 | 0.062 |
| rs5742915 | 15 | 72123686 | C | T | 0.451 | 0.031 |
| rs11259936 | 15 | 82371586 | C | A | 0.515 | 0.044 |
| rs16942341 | 15 | 87189909 | C | T | 0.972 | 0.13 |
| rs2871865 | 15 | 97012419 | C | G | 0.896 | 0.057 |
| rs4965598 | 15 | 98577137 | C | T | 0.304 | 0.028 |
| rs11648796 | 16 | 732191 | G | A | 0.316 | 0.034 |
| rs26868 | 16 | 2189377 | A | T | 0.431 | 0.034 |
| rs1659127 | 16 | 14295806 | A | G | 0.329 | 0.027 |
| rs8052560 | 16 | 87304743 | A | C | 0.769 | 0.029 |
| rs4640244 | 17 | 21224816 | A | G | 0.680 | 0.024 |
| rs3110496 | 17 | 24941897 | G | A | 0.680 | 0.022 |
| rs3764419 | 17 | 26188149 | C | A | 0.618 | 0.035 |
| rs17780086 | 17 | 27367395 | A | G | 0.117 | 0.028 |
| rs1043515 | 17 | 34175722 | G | A | 0.517 | 0.023 |
| rs4986172 | 17 | 40571807 | C | T | 0.621 | 0.032 |
| rs2072153 | 17 | 44745013 | C | G | 0.311 | 0.021 |
| rs4605213 | 17 | 46599746 | C | G | 0.349 | 0.021 |
| rs227724 | 17 | 52133816 | T | A | 0.342 | 0.03 |
| rs2079795 | 17 | 56851431 | T | C | 0.324 | 0.04 |
| rs2665838 | 17 | 59320197 | G | C | 0.257 | 0.042 |
| rs11867479 | 17 | 65601802 | T | C | 0.337 | 0.025 |
| rs4800452 | 18 | 18981609 | T | C | 0.784 | 0.051 |
| rs9967417 | 18 | 45213498 | G | C | 0.391 | 0.038 |
| rs17782313 | 18 | 56002077 | C | T | 0.230 | 0.028 |
| rs12982744 | 19 | 2128193 | G | C | 0.386 | 0.03 |
| rs7507204 | 19 | 3379834 | C | G | 0.239 | 0.036 |
| rs891088 | 19 | 7135762 | G | A | 0.269 | 0.029 |
| rs4072910 | 19 | 8550031 | G | C | 0.477 | 0.031 |
| rs2279008 | 19 | 17144303 | T | C | 0.736 | 0.025 |
| rs17318596 | 19 | 46628935 | A | G | 0.382 | 0.032 |
| rs1741344 | 20 | 4049800 | C | T | 0.334 | 0.023 |
| rs2145272 | 20 | 6574218 | G | A | 0.343 | 0.039 |
| rs7274811 | 20 | 31796842 | G | T | 0.761 | 0.041 |
| rs143384 | 20 | 33489170 | G | A | 0.415 | 0.063 |
| rs237743 | 20 | 47336426 | A | G | 0.243 | 0.041 |
| rs2834442 | 21 | 34612656 | A | T | 0.660 | 0.026 |
| rs4821083 | 22 | 33056341 | A | G | 0.852 | 0.031 |

Notes: Position from 1000 genomes build 37.

**Supplementary Table 2: BMI alleles, frequency and weight assigned in iCOGS (n= 41,062).**

| **SNP** | **Chr** | **Position (bp)** | **Effect allele** | **Other allele** | **Frequency of effect allele in iCOGs** | **Weight** |
| --- | --- | --- | --- | --- | --- | --- |
| rs2815752 | 1 | 72812440 | A | G | 0.614 | 0.13 |
| rs1514175 | 1 | 74991644 | A | G | 0.429 | 0.07 |
| rs1555543 | 1 | 96944797 | C | A | 0.604 | 0.06 |
| rs543874 | 1 | 177889480 | G | A | 0.197 | 0.22 |
| rs2867125 | 2 | 622827 | C | T | 0.826 | 0.31 |
| rs713586 | 2 | 25158008 | C | T | 0.460 | 0.14 |
| rs887912 | 2 | 59302877 | T | C | 0.274 | 0.1 |
| rs2890652 | 2 | 142959931 | C | T | 0.185 | 0.09 |
| rs13078807 | 3 | 85884150 | G | A | 0.186 | 0.1 |
| rs9816226 | 3 | 185834499 | T | A | 0.824 | 0.14 |
| rs10938397 | 4 | 45182527 | G | A | 0.431 | 0.18 |
| rs13107325 | 4 | 103188709 | T | C | 0.075 | 0.19 |
| rs2112347 | 5 | 75015242 | T | G | 0.627 | 0.1 |
| rs4836133 | 5 | 124332103 | A | C | 0.534 | 0.07 |
| rs206936 | 6 | 34302869 | G | A | 0.206 | 0.06 |
| rs987237 | 6 | 50803050 | G | A | 0.186 | 0.13 |
| rs10968576 | 9 | 28414339 | G | A | 0.319 | 0.11 |
| rs4929949 | 11 | 8604593 | C | T | 0.511 | 0.06 |
| rs10767664 | 11 | 27725986 | A | T | 0.792 | 0.19 |
| rs3817334 | 11 | 47650993 | T | C | 0.424 | 0.06 |
| rs7138803 | 12 | 50247468 | A | G | 0.385 | 0.12 |
| rs4771122 | 13 | 28020180 | G | A | 0.254 | 0.09 |
| rs11847697 | 14 | 30515112 | T | C | 0.054 | 0.17 |
| rs10150332 | 14 | 79936964 | C | T | 0.219 | 0.13 |
| rs2241423 | 15 | 68086838 | G | A | 0.783 | 0.13 |
| rs12444979 | 16 | 19933600 | C | T | 0.863 | 0.17 |
| rs7359397 | 16 | 28885659 | T | C | 0.394 | 0.15 |
| rs1558902 | 16 | 53803574 | A | T | 0.408 | 0.39 |
| rs571312 | 18 | 57839769 | A | C | 0.230 | 0.23 |
| rs29941 | 19 | 34309532 | G | A | 0.671 | 0.06 |
| rs2287019 | 19 | 46202172 | C | T | 0.781 | 0.15 |
| rs3810291 | 19 | 47569003 | A | G | 0.666 | 0.09 |

Notes: Position from 1000 genomes build 37.

**Supplementary Table 3: Height SNPs included in the independent split allele scores**

| **Independent allele score 1** | |  | **Independent allele score 2** | |
| --- | --- | --- | --- | --- |
| rs10010325 | rs425277 |  | rs7332115 | rs12982744 |
| rs1043515 | rs4282339 |  | rs2336725 | rs572169 |
| rs1046943 | rs4470914 |  | rs3782089 | rs11118346 |
| rs1047014 | rs4601530 |  | rs11648796 | rs6457620 |
| rs10748128 | rs4665736 |  | rs12680655 | rs11958779 |
| rs10799445 | rs473902 |  | rs12694997 | rs10037512 |
| rs10838801 | rs4800452 |  | rs1490384 | rs1570106 |
| rs11144688 | rs4965598 |  | rs1708299 | rs6569648 |
| rs11205277 | rs526896 |  | rs1046934 | rs494459 |
| rs11259936 | rs5742915 |  | rs227724 | rs6449353 |
| rs11599750 | rs634552 |  | rs16964211 | rs26868 |
| rs11830103 | rs6439167 |  | rs654723 | rs798489 |
| rs11867479 | rs6470764 |  | rs1468758 | rs12534093 |
| rs12153391 | rs6473015 |  | rs9863706 | rs3812163 |
| rs12470505 | rs6699417 |  | rs7274811 | rs2154319 |
| rs13088462 | rs6879260 |  | rs4821083 | rs3791675 |
| rs13177718 | rs7027110 |  | rs2145998 | rs2341459 |
| rs1325598 | rs7112925 |  | rs4640244 | rs11107116 |
| rs1351164 | rs7155279 |  | rs1257763 | rs2237886 |
| rs1351394 | rs7178424 |  | rs7849585 | rs6959212 |
| rs143384 | rs720390 |  | rs1173727 | rs2856321 |
| rs1659127 | rs724016 |  | rs1013209 | rs788867 |
| rs16942341 | rs7466269 |  | rs751543 | rs2580816 |
| rs17318596 | rs7507204 |  | rs7460090 | rs3129109 |
| rs17346452 | rs7532866 |  | rs9472414 | rs12474201 |
| rs1738475 | rs7567288 |  | rs1814175 | rs891088 |
| rs17780086 | rs7567851 |  | rs1330 | rs10863936 |
| rs17806888 | rs7689420 |  | rs3110496 | rs2066807 |
| rs2072153 | rs7759938 |  | rs10874746 | rs1582931 |
| rs2079795 | rs7853377 |  | rs2778031 | rs12902421 |
| rs2093210 | rs7864648 |  | rs6714546 | rs17511102 |
| rs2110001 | rs7909670 |  | rs9428104 | rs7763064 |
| rs2247341 | rs7926971 |  | rs9835332 | rs6684205 |
| rs2256183 | rs7971536 |  | rs4986172 | rs2145272 |
| rs2284746 | rs806794 |  | rs8052560 | rs10770705 |
| rs237743 | rs8181166 |  | rs422421 | rs2834442 |
| rs2597513 | rs822552 |  | rs9360921 | rs4605213 |
| rs2629046 | rs862034 |  | rs310405 | rs6457821 |
| rs2638953 | rs889014 |  | rs1950500 | rs1741344 |
| rs2665838 | rs9456307 |  | rs11684404 | rs543650 |
| rs274546 | rs955748 |  | rs17391694 | rs2871865 |
| rs2780226 | rs961764 |  | rs10152591 | rs17782313 |
| rs3118905 | rs9967417 |  | rs17081935 | rs7697556 |
| rs4072910 | rs9969804 |  | rs9844666 | rs7319045 |
| rs42235 |  |  | rs3764419 | rs2279008 |

**Supplementary Table 4: Association of individual height SNPs with prostate cancer outcomes. Odds-ratios indicate the effect of one extra height increasing allele.**

|  |  |  | Alleles | | Any prostate cancer (N=41,062) | | | |  | Localised prostate cancer (N=33,189) | | | |  | Advanced prostate cancer (N=24,539) | | | |  | Low grade prostate cancer (N=28,998) | | | |  | High grade prostate cancer (N=28,444) | | | |
| --- | --- | --- | --- | --- | --- | --- | --- | --- | --- | --- | --- | --- | --- | --- | --- | --- | --- | --- | --- | --- | --- | --- | --- | --- | --- | --- | --- | --- |
|  |  |  | Height |  | Odds | Confidence interval | | p- |  | Odds | Confidence interval | | p- |  | Odds | Confidence interval | | p- |  | Odds | Confidence interval | | p- |  | Odds | Confidence interval | | p- |
| Rs ID | Chr. | Position | increasing | Other | ratio | Lower | Upper | value |  | ratio | Lower | Upper | value |  | ratio | Lower | Upper | value |  | ratio | Lower | Upper | value |  | ratio | Lower | Upper | value |
| rs425277 | 1 | 2069172 | T | C | 1.04 | 1.02 | 1.07 | 0.002 |  | 1.07 | 1.03 | 1.12 | 0.001 |  | 1.00 | 0.97 | 1.03 | 0.94 |  | 1.07 | 1.03 | 1.10 | <0.001 |  | 1.04 | 1.00 | 1.07 | 0.04 |
| rs2284746 | 1 | 17306675 | G | C | 1.02 | 1.00 | 1.05 | 0.07 |  | 1.02 | 1.00 | 1.05 | 0.05 |  | 1.05 | 0.99 | 1.11 | 0.11 |  | 1.02 | 0.99 | 1.06 | 0.19 |  | 1.03 | 0.99 | 1.08 | 0.10 |
| rs1738475 | 1 | 23536891 | C | G | 1.02 | 1.01 | 1.04 | 0.01 |  | 1.02 | 1.00 | 1.05 | 0.06 |  | 1.02 | 1.00 | 1.05 | 0.07 |  | 1.02 | 1.00 | 1.04 | 0.04 |  | 1.03 | 1.01 | 1.06 | 0.02 |
| rs4601530 | 1 | 25044111 | C | T | 0.97 | 0.93 | 1.00 | 0.07 |  | 0.96 | 0.91 | 1.01 | 0.10 |  | 0.98 | 0.93 | 1.03 | 0.43 |  | 0.95 | 0.92 | 0.99 | 0.007 |  | 0.97 | 0.93 | 1.01 | 0.19 |
| rs7532866 | 1 | 26741544 | A | G | 1.02 | 1.00 | 1.04 | 0.03 |  | 1.01 | 0.99 | 1.04 | 0.27 |  | 1.04 | 1.01 | 1.07 | 0.02 |  | 1.02 | 0.99 | 1.05 | 0.23 |  | 1.02 | 0.99 | 1.06 | 0.13 |
| rs2154319 | 1 | 41745770 | C | T | 0.97 | 0.96 | 0.99 | 0.009 |  | 0.98 | 0.95 | 1.00 | 0.07 |  | 0.95 | 0.92 | 1.00 | 0.03 |  | 0.97 | 0.93 | 1.00 | 0.07 |  | 0.97 | 0.94 | 1.00 | 0.07 |
| rs17391694 | 1 | 78623626 | T | C | 1.01 | 0.98 | 1.05 | 0.50 |  | 1.01 | 0.97 | 1.05 | 0.58 |  | 0.99 | 0.91 | 1.08 | 0.82 |  | 1.02 | 0.97 | 1.06 | 0.51 |  | 0.98 | 0.94 | 1.03 | 0.51 |
| rs6699417 | 1 | 89123443 | T | C | 0.99 | 0.96 | 1.02 | 0.52 |  | 1.00 | 0.98 | 1.02 | 0.88 |  | 0.96 | 0.92 | 1.01 | 0.12 |  | 1.00 | 0.97 | 1.04 | 0.94 |  | 0.99 | 0.95 | 1.03 | 0.59 |
| rs10874746 | 1 | 93323971 | C | T | 0.96 | 0.94 | 0.99 | 0.009 |  | 0.98 | 0.95 | 1.01 | 0.14 |  | 0.96 | 0.92 | 0.99 | 0.02 |  | 0.97 | 0.94 | 1.01 | 0.13 |  | 0.96 | 0.93 | 0.99 | 0.02 |
| rs9428104 | 1 | 118855587 | G | A | 1.00 | 0.97 | 1.04 | 0.93 |  | 1.01 | 0.96 | 1.06 | 0.68 |  | 1.00 | 0.96 | 1.04 | 1.00 |  | 0.98 | 0.93 | 1.02 | 0.29 |  | 1.02 | 0.97 | 1.06 | 0.48 |
| rs11205277 | 1 | 149892872 | G | A | 0.98 | 0.95 | 1.00 | 0.08 |  | 0.98 | 0.95 | 1.01 | 0.29 |  | 0.98 | 0.93 | 1.03 | 0.44 |  | 0.97 | 0.94 | 1.00 | 0.03 |  | 1.00 | 0.96 | 1.03 | 0.80 |
| rs17346452 | 1 | 172053287 | C | T | 1.00 | 0.97 | 1.03 | 0.89 |  | 1.00 | 0.96 | 1.04 | 0.81 |  | 0.99 | 0.96 | 1.02 | 0.51 |  | 0.99 | 0.95 | 1.03 | 0.62 |  | 0.99 | 0.96 | 1.03 | 0.69 |
| rs1325598 | 1 | 176792249 | G | A | 1.00 | 0.95 | 1.06 | 0.90 |  | 1.02 | 0.97 | 1.07 | 0.46 |  | 1.00 | 0.95 | 1.07 | 0.88 |  | 1.01 | 0.95 | 1.08 | 0.65 |  | 1.00 | 0.96 | 1.04 | 0.95 |
| rs1046934 | 1 | 184023529 | C | A | 0.95 | 0.92 | 0.97 | <0.001 |  | 0.94 | 0.92 | 0.96 | <0.001 |  | 0.97 | 0.93 | 1.02 | 0.21 |  | 0.94 | 0.92 | 0.95 | <0.001 |  | 0.95 | 0.92 | 0.99 | 0.02 |
| rs10863936 | 1 | 212237798 | G | A | 0.99 | 0.97 | 1.02 | 0.64 |  | 0.99 | 0.97 | 1.01 | 0.36 |  | 1.00 | 0.96 | 1.05 | 0.93 |  | 1.01 | 0.99 | 1.04 | 0.27 |  | 0.99 | 0.96 | 1.02 | 0.40 |
| rs6684205 | 1 | 218609702 | G | A | 1.02 | 0.99 | 1.05 | 0.18 |  | 1.01 | 0.99 | 1.04 | 0.29 |  | 1.02 | 0.96 | 1.09 | 0.45 |  | 1.01 | 0.98 | 1.04 | 0.50 |  | 1.02 | 0.98 | 1.07 | 0.26 |
| rs11118346 | 1 | 219743719 | C | T | 1.00 | 0.97 | 1.04 | 0.84 |  | 1.02 | 0.98 | 1.06 | 0.31 |  | 0.98 | 0.93 | 1.03 | 0.40 |  | 1.04 | 0.99 | 1.09 | 0.10 |  | 0.97 | 0.93 | 1.00 | 0.09 |
| rs10799445 | 1 | 227911883 | A | C | 1.00 | 0.96 | 1.04 | 0.87 |  | 0.99 | 0.95 | 1.04 | 0.80 |  | 1.03 | 0.98 | 1.09 | 0.26 |  | 0.98 | 0.92 | 1.04 | 0.57 |  | 1.02 | 0.99 | 1.06 | 0.19 |
| rs4665736 | 2 | 25187599 | T | C | 0.99 | 0.97 | 1.02 | 0.64 |  | 1.00 | 0.97 | 1.03 | 0.99 |  | 0.99 | 0.95 | 1.03 | 0.55 |  | 0.98 | 0.94 | 1.02 | 0.29 |  | 1.02 | 0.99 | 1.05 | 0.16 |
| rs6714546 | 2 | 33361425 | G | A | 1.00 | 0.97 | 1.02 | 0.86 |  | 0.99 | 0.96 | 1.01 | 0.29 |  | 1.03 | 0.98 | 1.07 | 0.24 |  | 1.00 | 0.97 | 1.04 | 0.99 |  | 1.00 | 0.98 | 1.03 | 0.83 |
| rs17511102 | 2 | 37960613 | T | A | 0.99 | 0.94 | 1.05 | 0.80 |  | 0.99 | 0.93 | 1.04 | 0.68 |  | 0.97 | 0.89 | 1.06 | 0.49 |  | 0.97 | 0.91 | 1.04 | 0.43 |  | 0.99 | 0.93 | 1.06 | 0.83 |
| rs2341459 | 2 | 44768202 | T | C | 0.98 | 0.95 | 1.01 | 0.18 |  | 0.98 | 0.95 | 1.02 | 0.34 |  | 0.95 | 0.91 | 0.99 | 0.009 |  | 0.97 | 0.94 | 1.01 | 0.12 |  | 0.98 | 0.95 | 1.02 | 0.42 |
| rs12474201 | 2 | 46921285 | A | G | 1.00 | 0.97 | 1.04 | 0.78 |  | 1.01 | 0.98 | 1.04 | 0.45 |  | 1.03 | 0.98 | 1.09 | 0.25 |  | 0.98 | 0.93 | 1.03 | 0.36 |  | 1.06 | 1.03 | 1.09 | <0.001 |
| rs3791675 | 2 | 56111309 | C | T | 1.01 | 0.97 | 1.04 | 0.73 |  | 1.01 | 0.96 | 1.05 | 0.75 |  | 0.97 | 0.91 | 1.03 | 0.31 |  | 1.02 | 0.99 | 1.05 | 0.30 |  | 1.00 | 0.95 | 1.05 | 0.98 |
| rs11684404 | 2 | 88924622 | C | T | 1.02 | 0.99 | 1.04 | 0.15 |  | 1.00 | 0.98 | 1.03 | 0.76 |  | 1.02 | 0.96 | 1.08 | 0.59 |  | 1.03 | 0.99 | 1.07 | 0.11 |  | 1.01 | 0.98 | 1.04 | 0.45 |
| rs7567288 | 2 | 134434824 | C | T | 1.00 | 0.96 | 1.03 | 0.80 |  | 1.00 | 0.96 | 1.04 | 0.82 |  | 0.96 | 0.92 | 1.01 | 0.14 |  | 0.99 | 0.96 | 1.03 | 0.77 |  | 0.98 | 0.93 | 1.03 | 0.43 |
| rs7567851 | 2 | 178684720 | C | G | 1.08 | 1.04 | 1.12 | <0.001 |  | 1.06 | 1.01 | 1.11 | 0.02 |  | 1.17 | 1.10 | 1.24 | <0.001 |  | 1.08 | 1.04 | 1.11 | <0.001 |  | 1.13 | 1.09 | 1.18 | <0.001 |
| rs1351164 | 2 | 218271898 | T | C | 1.00 | 0.97 | 1.03 | 0.85 |  | 1.00 | 0.97 | 1.03 | 0.98 |  | 1.01 | 0.96 | 1.08 | 0.64 |  | 1.02 | 0.98 | 1.05 | 0.32 |  | 0.99 | 0.97 | 1.02 | 0.57 |
| rs12470505 | 2 | 219908369 | T | G | 1.04 | 0.99 | 1.09 | 0.10 |  | 1.06 | 1.00 | 1.12 | 0.06 |  | 0.99 | 0.91 | 1.07 | 0.82 |  | 1.05 | 0.98 | 1.12 | 0.17 |  | 1.03 | 0.97 | 1.09 | 0.36 |
| rs2629046 | 2 | 225047744 | T | C | 1.01 | 1.00 | 1.03 | 0.14 |  | 1.00 | 0.97 | 1.03 | 0.97 |  | 1.05 | 1.01 | 1.09 | 0.006 |  | 1.01 | 0.98 | 1.04 | 0.52 |  | 1.03 | 0.99 | 1.06 | 0.11 |
| rs2580816 | 2 | 232797966 | C | T | 0.96 | 0.94 | 0.99 | 0.009 |  | 0.96 | 0.92 | 1.01 | 0.10 |  | 0.98 | 0.93 | 1.03 | 0.44 |  | 0.92 | 0.88 | 0.96 | <0.001 |  | 1.01 | 0.98 | 1.05 | 0.48 |
| rs12694997 | 2 | 242262986 | G | A | 1.02 | 0.98 | 1.06 | 0.38 |  | 1.02 | 0.97 | 1.07 | 0.47 |  | 1.02 | 0.97 | 1.07 | 0.47 |  | 1.02 | 0.97 | 1.07 | 0.44 |  | 1.00 | 0.96 | 1.04 | 0.88 |
| rs2597513 | 3 | 13555836 | C | T | 1.00 | 0.97 | 1.03 | 0.95 |  | 1.02 | 0.99 | 1.05 | 0.19 |  | 0.97 | 0.88 | 1.07 | 0.53 |  | 1.02 | 0.98 | 1.05 | 0.30 |  | 1.00 | 0.96 | 1.04 | 0.92 |
| rs13088462 | 3 | 51071713 | C | T | 0.95 | 0.89 | 1.03 | 0.21 |  | 0.99 | 0.91 | 1.06 | 0.71 |  | 0.83 | 0.73 | 0.94 | 0.004 |  | 0.97 | 0.89 | 1.06 | 0.53 |  | 0.93 | 0.86 | 1.01 | 0.08 |
| rs2336725 | 3 | 53118739 | C | T | 1.02 | 0.98 | 1.05 | 0.34 |  | 1.02 | 0.98 | 1.06 | 0.36 |  | 1.01 | 0.96 | 1.06 | 0.62 |  | 1.02 | 0.98 | 1.07 | 0.31 |  | 1.01 | 0.97 | 1.05 | 0.67 |
| rs9835332 | 3 | 56667682 | G | C | 0.96 | 0.94 | 0.99 | 0.002 |  | 0.96 | 0.94 | 0.99 | 0.002 |  | 0.99 | 0.94 | 1.03 | 0.50 |  | 0.97 | 0.94 | 1.00 | 0.03 |  | 0.97 | 0.94 | 1.00 | 0.08 |
| rs17806888 | 3 | 67416322 | T | C | 1.01 | 0.97 | 1.05 | 0.64 |  | 1.00 | 0.94 | 1.05 | 0.90 |  | 1.00 | 0.92 | 1.09 | 0.94 |  | 0.99 | 0.93 | 1.05 | 0.82 |  | 1.03 | 0.98 | 1.08 | 0.23 |
| rs9863706 | 3 | 72437413 | C | T | 0.97 | 0.93 | 1.00 | 0.08 |  | 0.96 | 0.93 | 0.99 | 0.02 |  | 0.95 | 0.89 | 1.02 | 0.17 |  | 0.97 | 0.93 | 1.02 | 0.23 |  | 0.96 | 0.92 | 1.00 | 0.06 |
| rs6439167 | 3 | 129050756 | C | T | 1.02 | 0.98 | 1.07 | 0.29 |  | 1.03 | 0.98 | 1.08 | 0.31 |  | 0.95 | 0.88 | 1.04 | 0.26 |  | 1.02 | 0.97 | 1.08 | 0.40 |  | 1.00 | 0.95 | 1.05 | 0.89 |
| rs9844666 | 3 | 135974216 | G | A | 0.99 | 0.96 | 1.02 | 0.62 |  | 1.00 | 0.98 | 1.03 | 0.81 |  | 1.00 | 0.95 | 1.06 | 0.89 |  | 1.00 | 0.97 | 1.04 | 0.82 |  | 0.98 | 0.94 | 1.03 | 0.47 |
| rs724016 | 3 | 141105570 | G | A | 1.03 | 0.99 | 1.07 | 0.09 |  | 1.05 | 0.99 | 1.10 | 0.08 |  | 1.01 | 0.97 | 1.05 | 0.69 |  | 1.04 | 0.99 | 1.10 | 0.14 |  | 1.04 | 1.00 | 1.09 | 0.05 |
| rs572169 | 3 | 172165727 | T | C | 1.01 | 0.99 | 1.03 | 0.48 |  | 1.00 | 0.98 | 1.03 | 0.83 |  | 1.04 | 1.01 | 1.07 | 0.01 |  | 1.00 | 0.96 | 1.04 | 0.86 |  | 1.01 | 0.97 | 1.05 | 0.59 |
| rs720390 | 3 | 185548683 | A | G | 0.99 | 0.97 | 1.02 | 0.60 |  | 0.99 | 0.97 | 1.02 | 0.66 |  | 0.99 | 0.94 | 1.03 | 0.57 |  | 0.98 | 0.95 | 1.01 | 0.13 |  | 0.99 | 0.96 | 1.02 | 0.34 |
| rs2247341 | 4 | 1701317 | A | G | 1.00 | 0.98 | 1.02 | 0.86 |  | 1.00 | 0.98 | 1.02 | 0.96 |  | 1.01 | 0.95 | 1.07 | 0.86 |  | 1.00 | 0.97 | 1.02 | 0.77 |  | 1.00 | 0.98 | 1.04 | 0.75 |
| rs6449353 | 4 | 18033488 | T | C | 1.03 | 1.00 | 1.06 | 0.07 |  | 1.06 | 1.02 | 1.09 | 0.001 |  | 1.00 | 0.93 | 1.07 | 0.95 |  | 1.01 | 0.96 | 1.06 | 0.63 |  | 1.05 | 1.01 | 1.09 | 0.02 |
| rs17081935 | 4 | 57823476 | T | C | 0.97 | 0.93 | 1.01 | 0.14 |  | 0.96 | 0.93 | 0.99 | 0.03 |  | 0.99 | 0.91 | 1.06 | 0.71 |  | 0.96 | 0.92 | 1.00 | 0.04 |  | 0.98 | 0.93 | 1.03 | 0.38 |
| rs7697556 | 4 | 73515313 | T | C | 1.03 | 1.01 | 1.06 | 0.01 |  | 1.04 | 1.01 | 1.08 | 0.007 |  | 1.02 | 0.98 | 1.06 | 0.37 |  | 1.02 | 1.00 | 1.05 | 0.09 |  | 1.04 | 1.00 | 1.09 | 0.07 |
| rs788867 | 4 | 82150006 | G | T | 1.02 | 0.99 | 1.05 | 0.14 |  | 1.03 | 0.99 | 1.06 | 0.16 |  | 1.02 | 0.98 | 1.07 | 0.32 |  | 1.01 | 0.97 | 1.04 | 0.68 |  | 1.05 | 1.01 | 1.09 | 0.02 |
| rs10010325 | 4 | 106106353 | A | C | 1.00 | 0.97 | 1.03 | 0.98 |  | 1.01 | 0.97 | 1.05 | 0.62 |  | 0.95 | 0.90 | 1.01 | 0.10 |  | 1.02 | 0.98 | 1.06 | 0.41 |  | 0.98 | 0.94 | 1.03 | 0.40 |
| rs7689420 | 4 | 145568352 | C | T | 1.00 | 0.97 | 1.03 | 0.91 |  | 0.99 | 0.95 | 1.03 | 0.61 |  | 0.98 | 0.93 | 1.04 | 0.57 |  | 1.01 | 0.97 | 1.04 | 0.73 |  | 1.00 | 0.95 | 1.05 | 0.89 |
| rs955748 | 4 | 184215675 | G | A | 1.01 | 0.98 | 1.05 | 0.45 |  | 1.01 | 0.98 | 1.05 | 0.49 |  | 1.04 | 0.97 | 1.12 | 0.25 |  | 1.03 | 1.00 | 1.07 | 0.04 |  | 1.01 | 0.97 | 1.06 | 0.59 |
| rs1173727 | 5 | 32830521 | T | C | 1.01 | 0.99 | 1.04 | 0.41 |  | 1.01 | 0.97 | 1.05 | 0.67 |  | 1.02 | 0.99 | 1.05 | 0.23 |  | 1.02 | 0.99 | 1.05 | 0.22 |  | 1.01 | 0.98 | 1.05 | 0.41 |
| rs11958779 | 5 | 55001899 | G | A | 1.03 | 1.00 | 1.06 | 0.06 |  | 1.03 | 1.00 | 1.05 | 0.07 |  | 1.03 | 0.98 | 1.08 | 0.31 |  | 1.02 | 0.97 | 1.07 | 0.48 |  | 1.03 | 1.00 | 1.06 | 0.03 |
| rs10037512 | 5 | 88354675 | T | C | 1.01 | 0.98 | 1.04 | 0.55 |  | 1.01 | 0.98 | 1.04 | 0.55 |  | 1.04 | 1.00 | 1.09 | 0.07 |  | 1.00 | 0.97 | 1.03 | 0.79 |  | 1.03 | 0.98 | 1.07 | 0.21 |
| rs13177718 | 5 | 108113344 | C | T | 1.02 | 0.99 | 1.06 | 0.22 |  | 1.03 | 0.99 | 1.08 | 0.11 |  | 1.02 | 0.92 | 1.12 | 0.75 |  | 1.02 | 0.96 | 1.07 | 0.57 |  | 1.02 | 0.97 | 1.08 | 0.39 |
| rs1582931 | 5 | 122657199 | G | A | 1.03 | 1.00 | 1.05 | 0.02 |  | 1.03 | 0.99 | 1.06 | 0.13 |  | 1.04 | 0.98 | 1.10 | 0.24 |  | 1.03 | 1.01 | 1.05 | 0.01 |  | 1.01 | 0.97 | 1.05 | 0.54 |
| rs274546 | 5 | 131699867 | G | A | 1.01 | 0.98 | 1.03 | 0.64 |  | 1.00 | 0.97 | 1.03 | 0.93 |  | 1.02 | 0.98 | 1.07 | 0.32 |  | 1.01 | 0.98 | 1.03 | 0.64 |  | 1.01 | 0.97 | 1.04 | 0.75 |
| rs526896 | 5 | 134356705 | T | G | 0.99 | 0.96 | 1.01 | 0.25 |  | 1.00 | 0.97 | 1.03 | 0.77 |  | 1.01 | 0.96 | 1.05 | 0.76 |  | 0.99 | 0.96 | 1.03 | 0.73 |  | 0.98 | 0.94 | 1.02 | 0.25 |
| rs4282339 | 5 | 168256240 | G | A | 1.00 | 0.97 | 1.04 | 0.83 |  | 1.00 | 0.97 | 1.04 | 0.81 |  | 1.00 | 0.95 | 1.06 | 0.99 |  | 1.00 | 0.96 | 1.04 | 0.93 |  | 1.02 | 0.98 | 1.06 | 0.32 |
| rs12153391 | 5 | 171203438 | C | A | 1.00 | 0.96 | 1.03 | 0.80 |  | 1.00 | 0.97 | 1.03 | 0.94 |  | 1.00 | 0.90 | 1.10 | 0.93 |  | 1.00 | 0.96 | 1.04 | 0.93 |  | 0.99 | 0.95 | 1.03 | 0.71 |
| rs889014 | 5 | 172984114 | C | T | 1.02 | 0.99 | 1.04 | 0.23 |  | 1.02 | 1.00 | 1.05 | 0.11 |  | 0.97 | 0.93 | 1.02 | 0.27 |  | 1.02 | 1.00 | 1.05 | 0.07 |  | 1.01 | 0.98 | 1.04 | 0.57 |
| rs422421 | 5 | 176517326 | C | T | 1.00 | 0.95 | 1.04 | 0.93 |  | 0.99 | 0.94 | 1.05 | 0.80 |  | 1.02 | 0.97 | 1.07 | 0.38 |  | 0.99 | 0.93 | 1.05 | 0.79 |  | 1.02 | 0.98 | 1.06 | 0.41 |
| rs6879260 | 5 | 179731014 | C | T | 0.96 | 0.94 | 0.99 | 0.002 |  | 0.96 | 0.93 | 1.00 | 0.04 |  | 0.93 | 0.89 | 0.97 | 0.001 |  | 0.97 | 0.95 | 1.01 | 0.11 |  | 0.95 | 0.92 | 0.99 | 0.005 |
| rs3812163 | 6 | 7725760 | T | A | 1.01 | 0.98 | 1.04 | 0.64 |  | 1.01 | 0.98 | 1.05 | 0.43 |  | 1.03 | 0.98 | 1.08 | 0.31 |  | 1.04 | 1.00 | 1.07 | 0.06 |  | 0.99 | 0.95 | 1.03 | 0.56 |
| rs1047014 | 6 | 19841493 | C | T | 0.97 | 0.93 | 1.01 | 0.13 |  | 0.96 | 0.92 | 1.00 | 0.04 |  | 0.95 | 0.89 | 1.03 | 0.21 |  | 0.97 | 0.91 | 1.04 | 0.41 |  | 0.97 | 0.92 | 1.01 | 0.11 |
| rs806794 | 6 | 26200677 | A | G | 0.98 | 0.96 | 1.01 | 0.25 |  | 0.98 | 0.95 | 1.02 | 0.37 |  | 0.94 | 0.90 | 0.99 | 0.01 |  | 0.98 | 0.95 | 1.01 | 0.18 |  | 0.98 | 0.95 | 1.01 | 0.18 |
| rs3129109 | 6 | 29084232 | C | T | 0.97 | 0.94 | 1.00 | 0.04 |  | 0.95 | 0.92 | 0.98 | 0.003 |  | 1.02 | 0.96 | 1.08 | 0.50 |  | 0.94 | 0.90 | 0.97 | <0.001 |  | 0.99 | 0.96 | 1.03 | 0.73 |
| rs114684762 | 6 | 31380529 | A | G | 1.00 | 0.97 | 1.03 | 0.99 |  | 1.00 | 0.97 | 1.03 | 0.84 |  | 0.99 | 0.95 | 1.03 | 0.60 |  | 1.00 | 0.96 | 1.03 | 0.77 |  | 1.01 | 0.98 | 1.04 | 0.54 |
| rs115961701 | 6 | 32663999 | G | C | 0.97 | 0.95 | 0.99 | 0.001 |  | 0.96 | 0.93 | 0.99 | 0.01 |  | 0.96 | 0.90 | 1.01 | 0.14 |  | 0.96 | 0.92 | 0.99 | 0.02 |  | 0.97 | 0.94 | 1.01 | 0.13 |
| rs2780226 | 6 | 34199092 | C | T | 1.01 | 0.95 | 1.08 | 0.67 |  | 1.03 | 0.97 | 1.09 | 0.31 |  | 1.00 | 0.89 | 1.12 | 0.98 |  | 1.02 | 0.97 | 1.07 | 0.49 |  | 1.03 | 0.96 | 1.11 | 0.37 |
| rs6457821 | 6 | 35402805 | C | A | 1.04 | 0.95 | 1.13 | 0.41 |  | 1.01 | 0.90 | 1.12 | 0.91 |  | 1.08 | 0.94 | 1.25 | 0.30 |  | 1.00 | 0.86 | 1.17 | 0.97 |  | 1.08 | 0.93 | 1.26 | 0.31 |
| rs9472414 | 6 | 44946506 | T | A | 0.99 | 0.95 | 1.02 | 0.43 |  | 0.98 | 0.95 | 1.02 | 0.43 |  | 0.96 | 0.91 | 1.02 | 0.19 |  | 1.00 | 0.95 | 1.06 | 0.88 |  | 0.98 | 0.94 | 1.02 | 0.38 |
| rs9360921 | 6 | 76265642 | G | T | 1.02 | 0.97 | 1.07 | 0.48 |  | 1.02 | 0.95 | 1.09 | 0.58 |  | 1.00 | 0.90 | 1.11 | 0.99 |  | 1.01 | 0.96 | 1.06 | 0.69 |  | 1.03 | 0.96 | 1.10 | 0.43 |
| rs310405 | 6 | 81800362 | A | G | 1.01 | 0.99 | 1.03 | 0.44 |  | 1.01 | 0.98 | 1.04 | 0.49 |  | 0.99 | 0.97 | 1.02 | 0.73 |  | 1.01 | 0.97 | 1.07 | 0.57 |  | 0.99 | 0.96 | 1.02 | 0.62 |
| rs7759938 | 6 | 105378954 | T | C | 1.01 | 0.98 | 1.03 | 0.71 |  | 1.02 | 0.99 | 1.05 | 0.15 |  | 0.97 | 0.93 | 1.01 | 0.14 |  | 1.01 | 0.99 | 1.04 | 0.36 |  | 0.99 | 0.95 | 1.04 | 0.73 |
| rs1046943 | 6 | 109783941 | G | A | 0.98 | 0.96 | 1.00 | 0.09 |  | 0.97 | 0.94 | 1.00 | 0.02 |  | 0.99 | 0.94 | 1.04 | 0.64 |  | 0.96 | 0.93 | 0.99 | 0.02 |  | 0.99 | 0.95 | 1.02 | 0.44 |
| rs961764 | 6 | 117522156 | C | G | 1.00 | 0.97 | 1.04 | 0.90 |  | 1.00 | 0.96 | 1.04 | 0.94 |  | 1.02 | 0.98 | 1.05 | 0.30 |  | 1.03 | 0.99 | 1.06 | 0.13 |  | 0.99 | 0.95 | 1.03 | 0.70 |
| rs1490384 | 6 | 126851160 | C | T | 0.98 | 0.95 | 1.00 | 0.07 |  | 0.97 | 0.95 | 1.00 | 0.04 |  | 0.99 | 0.96 | 1.03 | 0.78 |  | 0.98 | 0.94 | 1.01 | 0.18 |  | 0.99 | 0.95 | 1.02 | 0.48 |
| rs6569648 | 6 | 130349119 | C | T | 0.96 | 0.92 | 0.99 | 0.02 |  | 0.96 | 0.92 | 0.99 | 0.02 |  | 0.95 | 0.91 | 1.00 | 0.04 |  | 0.97 | 0.93 | 1.00 | 0.05 |  | 0.93 | 0.89 | 0.98 | 0.008 |
| rs7763064 | 6 | 142797289 | G | A | 1.01 | 0.99 | 1.04 | 0.25 |  | 1.01 | 0.98 | 1.03 | 0.53 |  | 1.03 | 0.98 | 1.08 | 0.18 |  | 1.02 | 0.99 | 1.06 | 0.16 |  | 1.02 | 0.98 | 1.05 | 0.42 |
| rs543650 | 6 | 152110943 | G | T | 1.01 | 0.98 | 1.03 | 0.57 |  | 1.00 | 0.98 | 1.03 | 0.84 |  | 1.01 | 0.98 | 1.05 | 0.46 |  | 1.00 | 0.96 | 1.03 | 0.82 |  | 1.00 | 0.96 | 1.04 | 0.94 |
| rs9456307 | 6 | 158929442 | T | A | 1.06 | 0.99 | 1.13 | 0.10 |  | 1.03 | 0.98 | 1.10 | 0.26 |  | 1.07 | 0.91 | 1.26 | 0.43 |  | 1.10 | 1.00 | 1.21 | 0.04 |  | 1.03 | 0.93 | 1.15 | 0.53 |
| rs798489 | 7 | 2801803 | C | T | 0.99 | 0.95 | 1.03 | 0.65 |  | 0.99 | 0.94 | 1.04 | 0.64 |  | 0.98 | 0.93 | 1.04 | 0.53 |  | 0.99 | 0.94 | 1.04 | 0.70 |  | 1.00 | 0.95 | 1.04 | 0.85 |
| rs4470914 | 7 | 19616522 | T | C | 1.04 | 1.00 | 1.07 | 0.03 |  | 1.03 | 0.99 | 1.06 | 0.12 |  | 1.05 | 0.99 | 1.12 | 0.13 |  | 1.02 | 0.98 | 1.06 | 0.33 |  | 1.05 | 1.00 | 1.11 | 0.05 |
| rs12534093 | 7 | 23502974 | T | A | 1.00 | 0.98 | 1.02 | 0.90 |  | 0.99 | 0.95 | 1.03 | 0.56 |  | 1.02 | 0.97 | 1.07 | 0.52 |  | 1.01 | 0.98 | 1.04 | 0.57 |  | 0.99 | 0.95 | 1.03 | 0.53 |
| rs1708299 | 7 | 28189946 | A | G | 0.99 | 0.95 | 1.02 | 0.42 |  | 0.98 | 0.95 | 1.02 | 0.32 |  | 0.95 | 0.89 | 1.02 | 0.18 |  | 0.99 | 0.96 | 1.02 | 0.54 |  | 1.00 | 0.95 | 1.04 | 0.87 |
| rs6959212 | 7 | 38128326 | C | T | 1.00 | 0.97 | 1.03 | 0.94 |  | 1.00 | 0.96 | 1.05 | 0.86 |  | 1.00 | 0.97 | 1.03 | 0.95 |  | 0.98 | 0.94 | 1.02 | 0.29 |  | 1.03 | 1.00 | 1.06 | 0.10 |
| rs42235 | 7 | 92248076 | T | C | 1.02 | 0.99 | 1.05 | 0.25 |  | 1.03 | 0.99 | 1.06 | 0.18 |  | 1.01 | 0.98 | 1.04 | 0.47 |  | 1.03 | 0.99 | 1.07 | 0.14 |  | 1.03 | 0.99 | 1.07 | 0.21 |
| rs822552 | 7 | 148650634 | G | C | 1.03 | 0.99 | 1.06 | 0.12 |  | 1.03 | 0.99 | 1.07 | 0.15 |  | 1.01 | 0.98 | 1.03 | 0.64 |  | 1.05 | 1.01 | 1.09 | 0.01 |  | 1.00 | 0.95 | 1.05 | 0.91 |
| rs77505230 | 7 | 150517022 | G | C | 1.01 | 0.98 | 1.05 | 0.33 |  | 1.01 | 0.98 | 1.05 | 0.38 |  | 1.01 | 0.97 | 1.05 | 0.71 |  | 1.00 | 0.95 | 1.05 | 0.88 |  | 1.04 | 1.00 | 1.08 | 0.06 |
| rs1013209 | 8 | 24116304 | C | T | 1.01 | 0.97 | 1.04 | 0.74 |  | 1.01 | 0.97 | 1.05 | 0.64 |  | 0.96 | 0.90 | 1.01 | 0.12 |  | 1.01 | 0.97 | 1.05 | 0.65 |  | 0.99 | 0.94 | 1.04 | 0.64 |
| rs7460090 | 8 | 57194163 | T | C | 1.00 | 0.95 | 1.05 | 0.93 |  | 0.99 | 0.94 | 1.04 | 0.62 |  | 1.00 | 0.91 | 1.10 | 0.98 |  | 1.04 | 0.98 | 1.10 | 0.20 |  | 0.97 | 0.90 | 1.04 | 0.41 |
| rs6473015 | 8 | 78178485 | C | A | 1.03 | 1.00 | 1.05 | 0.04 |  | 1.03 | 1.00 | 1.05 | 0.03 |  | 1.03 | 0.98 | 1.08 | 0.20 |  | 1.01 | 0.99 | 1.04 | 0.37 |  | 1.05 | 1.01 | 1.10 | 0.02 |
| rs6470764 | 8 | 130725665 | C | T | 0.99 | 0.96 | 1.03 | 0.69 |  | 1.00 | 0.96 | 1.03 | 0.90 |  | 0.99 | 0.95 | 1.03 | 0.69 |  | 0.98 | 0.93 | 1.03 | 0.36 |  | 1.01 | 0.97 | 1.06 | 0.61 |
| rs12680655 | 8 | 135637337 | C | G | 0.99 | 0.95 | 1.03 | 0.55 |  | 0.99 | 0.95 | 1.03 | 0.56 |  | 0.98 | 0.95 | 1.02 | 0.45 |  | 0.99 | 0.95 | 1.04 | 0.83 |  | 0.96 | 0.93 | 1.00 | 0.05 |
| rs7864648 | 9 | 16368732 | T | G | 0.99 | 0.95 | 1.03 | 0.54 |  | 0.99 | 0.95 | 1.04 | 0.74 |  | 0.99 | 0.95 | 1.02 | 0.44 |  | 0.99 | 0.95 | 1.04 | 0.75 |  | 0.97 | 0.94 | 1.01 | 0.13 |
| rs11144688 | 9 | 78542286 | G | A | 1.03 | 0.97 | 1.10 | 0.38 |  | 1.03 | 0.97 | 1.10 | 0.36 |  | 1.01 | 0.92 | 1.12 | 0.78 |  | 1.01 | 0.94 | 1.09 | 0.74 |  | 1.01 | 0.92 | 1.12 | 0.82 |
| rs7853377 | 9 | 86552205 | G | A | 0.96 | 0.92 | 1.00 | 0.05 |  | 0.97 | 0.93 | 1.00 | 0.07 |  | 0.94 | 0.87 | 1.01 | 0.09 |  | 0.96 | 0.92 | 0.99 | 0.02 |  | 0.94 | 0.88 | 0.99 | 0.03 |
| rs8181166 | 9 | 89116628 | C | G | 0.99 | 0.97 | 1.02 | 0.54 |  | 1.00 | 0.98 | 1.03 | 0.76 |  | 0.96 | 0.93 | 1.00 | 0.07 |  | 0.98 | 0.96 | 1.01 | 0.26 |  | 1.01 | 0.99 | 1.04 | 0.35 |
| rs2778031 | 9 | 90835726 | T | C | 1.01 | 0.97 | 1.04 | 0.69 |  | 1.00 | 0.97 | 1.04 | 0.91 |  | 1.05 | 0.98 | 1.12 | 0.14 |  | 1.01 | 0.96 | 1.06 | 0.72 |  | 1.01 | 0.97 | 1.04 | 0.76 |
| rs9969804 | 9 | 95429120 | A | C | 0.98 | 0.94 | 1.01 | 0.19 |  | 0.97 | 0.93 | 1.01 | 0.09 |  | 0.99 | 0.93 | 1.05 | 0.70 |  | 0.96 | 0.92 | 1.01 | 0.12 |  | 0.99 | 0.94 | 1.04 | 0.73 |
| rs1257763 | 9 | 96893945 | A | G | 1.08 | 1.01 | 1.16 | 0.03 |  | 1.10 | 1.02 | 1.19 | 0.01 |  | 1.00 | 0.87 | 1.14 | 0.98 |  | 1.09 | 1.02 | 1.17 | 0.02 |  | 1.04 | 0.92 | 1.16 | 0.55 |
| rs473902 | 9 | 98256235 | T | G | 1.05 | 0.99 | 1.10 | 0.08 |  | 1.01 | 0.96 | 1.07 | 0.62 |  | 1.11 | 1.00 | 1.22 | 0.04 |  | 1.00 | 0.95 | 1.05 | 0.90 |  | 1.07 | 1.00 | 1.15 | 0.07 |
| rs7027110 | 9 | 109599046 | A | G | 1.02 | 0.98 | 1.06 | 0.41 |  | 1.02 | 0.98 | 1.07 | 0.30 |  | 1.00 | 0.96 | 1.05 | 0.91 |  | 1.04 | 1.00 | 1.08 | 0.07 |  | 1.00 | 0.96 | 1.04 | 0.98 |
| rs1468758 | 9 | 113807082 | C | T | 0.99 | 0.97 | 1.02 | 0.65 |  | 0.98 | 0.95 | 1.01 | 0.17 |  | 1.00 | 0.94 | 1.06 | 0.98 |  | 1.00 | 0.96 | 1.04 | 0.82 |  | 0.98 | 0.94 | 1.02 | 0.35 |
| rs751543 | 9 | 119122342 | T | C | 1.01 | 0.97 | 1.05 | 0.62 |  | 1.01 | 0.98 | 1.05 | 0.45 |  | 1.02 | 0.96 | 1.09 | 0.54 |  | 1.01 | 0.97 | 1.06 | 0.59 |  | 1.01 | 0.97 | 1.05 | 0.54 |
| rs7466269 | 9 | 133464084 | A | G | 1.00 | 0.98 | 1.02 | 0.98 |  | 1.00 | 0.97 | 1.03 | 0.97 |  | 1.03 | 0.98 | 1.08 | 0.32 |  | 1.02 | 0.99 | 1.05 | 0.28 |  | 1.00 | 0.96 | 1.03 | 0.82 |
| rs7849585 | 9 | 139111870 | T | G | 1.00 | 0.98 | 1.02 | 0.96 |  | 0.99 | 0.97 | 1.02 | 0.47 |  | 1.02 | 0.98 | 1.05 | 0.40 |  | 1.01 | 0.98 | 1.05 | 0.46 |  | 0.98 | 0.95 | 1.00 | 0.10 |
| rs7909670 | 10 | 12918764 | C | T | 0.99 | 0.97 | 1.01 | 0.38 |  | 0.99 | 0.96 | 1.02 | 0.38 |  | 0.97 | 0.93 | 1.01 | 0.14 |  | 0.97 | 0.94 | 1.00 | 0.04 |  | 0.99 | 0.96 | 1.02 | 0.60 |
| rs2145998 | 10 | 81121696 | T | A | 1.00 | 0.98 | 1.02 | 0.70 |  | 1.01 | 0.98 | 1.04 | 0.49 |  | 0.97 | 0.93 | 1.01 | 0.09 |  | 1.01 | 0.98 | 1.04 | 0.55 |  | 0.99 | 0.96 | 1.01 | 0.37 |
| rs11599750 | 10 | 101805442 | C | T | 1.03 | 1.00 | 1.05 | 0.03 |  | 1.03 | 1.00 | 1.06 | 0.06 |  | 1.00 | 0.95 | 1.04 | 0.91 |  | 1.00 | 0.96 | 1.03 | 0.92 |  | 1.06 | 1.02 | 1.10 | 0.008 |
| rs2237886 | 11 | 2810731 | T | C | 0.96 | 0.91 | 1.01 | 0.14 |  | 0.96 | 0.90 | 1.02 | 0.17 |  | 0.98 | 0.92 | 1.03 | 0.42 |  | 0.92 | 0.86 | 0.99 | 0.02 |  | 0.98 | 0.93 | 1.03 | 0.34 |
| rs7926971 | 11 | 12698040 | G | A | 1.01 | 0.98 | 1.04 | 0.59 |  | 1.02 | 0.98 | 1.06 | 0.27 |  | 1.02 | 0.99 | 1.05 | 0.18 |  | 1.00 | 0.96 | 1.04 | 0.90 |  | 1.02 | 0.99 | 1.06 | 0.22 |
| rs1330 | 11 | 17316029 | T | C | 1.00 | 0.97 | 1.03 | 0.82 |  | 1.02 | 0.99 | 1.05 | 0.32 |  | 0.96 | 0.92 | 1.00 | 0.07 |  | 1.02 | 0.98 | 1.05 | 0.33 |  | 0.98 | 0.95 | 1.02 | 0.35 |
| rs79890777 | 11 | 48098280 | G | A | 0.98 | 0.96 | 1.00 | 0.02 |  | 0.98 | 0.96 | 1.01 | 0.16 |  | 0.97 | 0.93 | 1.01 | 0.14 |  | 0.96 | 0.94 | 0.99 | 0.003 |  | 1.00 | 0.97 | 1.03 | 0.94 |
| rs1814175 | 11 | 49559172 | T | C | 0.97 | 0.95 | 0.99 | <0.001 |  | 0.97 | 0.95 | 1.00 | 0.03 |  | 0.95 | 0.92 | 0.99 | 0.02 |  | 0.95 | 0.93 | 0.99 | 0.004 |  | 0.98 | 0.95 | 1.01 | 0.22 |
| rs3782089 | 11 | 65336819 | C | T | 0.96 | 0.89 | 1.03 | 0.24 |  | 1.00 | 0.92 | 1.08 | 0.92 |  | 0.94 | 0.85 | 1.04 | 0.23 |  | 0.98 | 0.88 | 1.09 | 0.70 |  | 0.97 | 0.90 | 1.05 | 0.48 |
| rs7112925 | 11 | 66826160 | C | T | 0.97 | 0.94 | 1.00 | 0.04 |  | 0.96 | 0.93 | 1.00 | 0.04 |  | 0.96 | 0.93 | 0.99 | 0.02 |  | 0.96 | 0.93 | 0.99 | 0.02 |  | 0.99 | 0.95 | 1.02 | 0.43 |
| rs634552 | 11 | 75282052 | T | G | 0.99 | 0.95 | 1.03 | 0.64 |  | 0.97 | 0.93 | 1.01 | 0.14 |  | 1.03 | 0.93 | 1.14 | 0.55 |  | 0.99 | 0.95 | 1.04 | 0.81 |  | 0.97 | 0.92 | 1.03 | 0.33 |
| rs494459 | 11 | 118574675 | T | C | 0.99 | 0.95 | 1.03 | 0.53 |  | 0.98 | 0.94 | 1.02 | 0.30 |  | 1.01 | 0.96 | 1.06 | 0.82 |  | 1.00 | 0.96 | 1.04 | 0.88 |  | 0.98 | 0.93 | 1.03 | 0.34 |
| rs654723 | 11 | 128586155 | A | C | 1.00 | 0.97 | 1.04 | 0.83 |  | 1.00 | 0.97 | 1.04 | 0.89 |  | 0.98 | 0.92 | 1.03 | 0.43 |  | 0.99 | 0.96 | 1.03 | 0.78 |  | 1.01 | 0.96 | 1.05 | 0.74 |
| rs2856321 | 12 | 11855773 | G | A | 1.03 | 1.00 | 1.05 | 0.04 |  | 1.03 | 1.00 | 1.06 | 0.02 |  | 1.03 | 0.99 | 1.07 | 0.17 |  | 1.04 | 1.00 | 1.07 | 0.02 |  | 1.02 | 0.98 | 1.07 | 0.29 |
| rs10770705 | 12 | 20857467 | A | C | 0.99 | 0.94 | 1.03 | 0.54 |  | 0.97 | 0.92 | 1.03 | 0.31 |  | 1.02 | 0.93 | 1.12 | 0.63 |  | 0.98 | 0.92 | 1.04 | 0.53 |  | 1.00 | 0.95 | 1.06 | 0.95 |
| rs2638953 | 12 | 28534415 | C | G | 1.01 | 0.99 | 1.03 | 0.21 |  | 1.02 | 1.00 | 1.04 | 0.03 |  | 0.96 | 0.91 | 1.00 | 0.06 |  | 1.01 | 0.98 | 1.05 | 0.43 |  | 1.01 | 0.97 | 1.05 | 0.69 |
| rs2066807 | 12 | 56740682 | G | C | 1.00 | 0.94 | 1.05 | 0.91 |  | 1.01 | 0.95 | 1.08 | 0.65 |  | 0.94 | 0.83 | 1.07 | 0.35 |  | 1.02 | 0.96 | 1.09 | 0.50 |  | 1.00 | 0.93 | 1.08 | 0.90 |
| rs1351394 | 12 | 66351826 | T | C | 1.01 | 0.98 | 1.04 | 0.39 |  | 1.02 | 0.99 | 1.06 | 0.16 |  | 1.00 | 0.95 | 1.06 | 0.94 |  | 1.00 | 0.97 | 1.04 | 0.79 |  | 1.03 | 0.99 | 1.08 | 0.15 |
| rs10748128 | 12 | 69827658 | T | G | 1.02 | 0.98 | 1.07 | 0.35 |  | 1.03 | 0.98 | 1.08 | 0.31 |  | 1.04 | 0.98 | 1.11 | 0.23 |  | 1.03 | 0.98 | 1.08 | 0.23 |  | 1.03 | 0.98 | 1.08 | 0.28 |
| rs11107116 | 12 | 93978504 | T | G | 0.94 | 0.91 | 0.96 | <0.001 |  | 0.93 | 0.91 | 0.96 | <0.001 |  | 0.96 | 0.90 | 1.02 | 0.15 |  | 0.93 | 0.89 | 0.97 | <0.001 |  | 0.96 | 0.94 | 0.99 | 0.01 |
| rs7971536 | 12 | 102373788 | T | A | 1.00 | 0.96 | 1.04 | 0.98 |  | 1.00 | 0.95 | 1.04 | 0.84 |  | 0.99 | 0.93 | 1.04 | 0.62 |  | 1.01 | 0.97 | 1.06 | 0.56 |  | 0.98 | 0.93 | 1.04 | 0.55 |
| rs11830103 | 12 | 123823546 | G | A | 0.95 | 0.92 | 0.99 | 0.008 |  | 0.95 | 0.91 | 0.99 | 0.03 |  | 0.93 | 0.88 | 0.99 | 0.02 |  | 0.98 | 0.94 | 1.02 | 0.36 |  | 0.93 | 0.88 | 0.98 | 0.004 |
| rs7332115 | 13 | 33147548 | G | T | 0.97 | 0.95 | 0.99 | 0.009 |  | 0.95 | 0.94 | 0.97 | <0.001 |  | 1.00 | 0.96 | 1.04 | 1.00 |  | 0.98 | 0.95 | 1.01 | 0.22 |  | 0.96 | 0.93 | 1.00 | 0.03 |
| rs3118905 | 13 | 51105334 | G | A | 0.98 | 0.95 | 1.01 | 0.14 |  | 0.97 | 0.93 | 1.01 | 0.15 |  | 0.99 | 0.93 | 1.05 | 0.69 |  | 0.96 | 0.92 | 1.01 | 0.15 |  | 0.98 | 0.95 | 1.01 | 0.20 |
| rs7319045 | 13 | 92024574 | A | G | 1.02 | 0.99 | 1.04 | 0.20 |  | 1.01 | 0.98 | 1.04 | 0.44 |  | 1.03 | 0.98 | 1.08 | 0.26 |  | 1.01 | 0.98 | 1.05 | 0.47 |  | 1.03 | 1.00 | 1.06 | 0.08 |
| rs1950500 | 14 | 24830850 | T | C | 0.99 | 0.96 | 1.01 | 0.28 |  | 0.99 | 0.95 | 1.03 | 0.55 |  | 0.97 | 0.92 | 1.02 | 0.18 |  | 1.02 | 0.99 | 1.06 | 0.26 |  | 0.96 | 0.92 | 0.99 | 0.02 |
| rs2093210 | 14 | 60957279 | C | T | 1.00 | 0.97 | 1.02 | 0.75 |  | 1.00 | 0.96 | 1.04 | 0.86 |  | 0.97 | 0.93 | 1.01 | 0.12 |  | 0.97 | 0.94 | 1.01 | 0.14 |  | 1.02 | 0.99 | 1.06 | 0.19 |
| rs1570106 | 14 | 68813115 | C | T | 0.98 | 0.94 | 1.01 | 0.20 |  | 0.98 | 0.94 | 1.03 | 0.47 |  | 0.98 | 0.92 | 1.04 | 0.49 |  | 0.97 | 0.94 | 1.01 | 0.18 |  | 0.98 | 0.93 | 1.04 | 0.55 |
| rs862034 | 14 | 74990746 | G | A | 0.98 | 0.96 | 1.01 | 0.20 |  | 0.98 | 0.95 | 1.01 | 0.21 |  | 0.97 | 0.91 | 1.05 | 0.49 |  | 1.01 | 0.97 | 1.05 | 0.66 |  | 0.95 | 0.92 | 0.99 | 0.02 |
| rs7155279 | 14 | 92485881 | G | T | 0.97 | 0.94 | 1.00 | 0.05 |  | 0.96 | 0.93 | 1.00 | 0.07 |  | 0.96 | 0.93 | 1.00 | 0.04 |  | 0.95 | 0.91 | 1.00 | 0.03 |  | 0.99 | 0.96 | 1.03 | 0.62 |
| rs16964211 | 15 | 51530495 | G | A | 1.01 | 0.96 | 1.06 | 0.75 |  | 1.00 | 0.93 | 1.07 | 0.93 |  | 1.00 | 0.93 | 1.08 | 0.94 |  | 1.04 | 0.94 | 1.15 | 0.43 |  | 1.01 | 0.95 | 1.08 | 0.71 |
| rs7178424 | 15 | 62380259 | C | T | 1.00 | 0.97 | 1.03 | 0.90 |  | 0.99 | 0.95 | 1.03 | 0.57 |  | 1.00 | 0.96 | 1.05 | 0.84 |  | 1.01 | 0.96 | 1.06 | 0.63 |  | 0.97 | 0.94 | 1.00 | 0.05 |
| rs10152591 | 15 | 70048157 | A | C | 0.97 | 0.93 | 1.01 | 0.16 |  | 0.97 | 0.93 | 1.02 | 0.23 |  | 0.92 | 0.85 | 1.00 | 0.04 |  | 0.96 | 0.93 | 1.00 | 0.08 |  | 0.96 | 0.92 | 1.01 | 0.12 |
| rs12902421 | 15 | 72161403 | C | T | 0.92 | 0.80 | 1.06 | 0.26 |  | 0.89 | 0.73 | 1.08 | 0.25 |  | 1.08 | 0.83 | 1.41 | 0.57 |  | 0.89 | 0.73 | 1.10 | 0.29 |  | 0.99 | 0.87 | 1.13 | 0.94 |
| rs5742915 | 15 | 74336633 | C | T | 1.00 | 0.98 | 1.02 | 0.94 |  | 0.99 | 0.96 | 1.02 | 0.40 |  | 1.03 | 0.99 | 1.07 | 0.17 |  | 1.00 | 0.96 | 1.03 | 0.82 |  | 0.99 | 0.96 | 1.02 | 0.63 |
| rs11259936 | 15 | 84580582 | C | A | 0.98 | 0.96 | 1.01 | 0.15 |  | 0.99 | 0.97 | 1.02 | 0.68 |  | 0.96 | 0.93 | 1.00 | 0.06 |  | 0.98 | 0.95 | 1.01 | 0.20 |  | 0.98 | 0.95 | 1.02 | 0.40 |
| rs16942341 | 15 | 89388905 | C | T | 1.00 | 0.92 | 1.10 | 0.92 |  | 0.97 | 0.88 | 1.07 | 0.49 |  | 1.02 | 0.82 | 1.27 | 0.87 |  | 0.96 | 0.84 | 1.09 | 0.51 |  | 1.02 | 0.94 | 1.12 | 0.61 |
| rs2871865 | 15 | 99194896 | C | G | 0.99 | 0.95 | 1.03 | 0.51 |  | 0.99 | 0.93 | 1.05 | 0.78 |  | 0.97 | 0.92 | 1.01 | 0.13 |  | 1.00 | 0.93 | 1.07 | 0.96 |  | 0.97 | 0.93 | 1.01 | 0.16 |
| rs4965598 | 15 | 100759614 | C | T | 0.95 | 0.92 | 0.99 | 0.007 |  | 0.97 | 0.93 | 1.00 | 0.06 |  | 0.94 | 0.88 | 1.00 | 0.05 |  | 0.96 | 0.92 | 1.01 | 0.11 |  | 0.95 | 0.91 | 0.99 | 0.01 |
| rs11648796 | 16 | 792190 | G | A | 0.98 | 0.95 | 1.01 | 0.17 |  | 0.98 | 0.94 | 1.01 | 0.18 |  | 1.00 | 0.93 | 1.06 | 0.93 |  | 0.98 | 0.93 | 1.03 | 0.36 |  | 0.99 | 0.96 | 1.02 | 0.54 |
| rs26868 | 16 | 2249376 | A | T | 1.00 | 0.97 | 1.04 | 0.81 |  | 1.00 | 0.95 | 1.05 | 0.91 |  | 0.99 | 0.95 | 1.03 | 0.54 |  | 1.01 | 0.96 | 1.06 | 0.80 |  | 0.99 | 0.96 | 1.03 | 0.66 |
| rs1659127 | 16 | 14388305 | A | G | 1.02 | 0.99 | 1.05 | 0.22 |  | 1.02 | 0.99 | 1.06 | 0.17 |  | 1.01 | 0.98 | 1.05 | 0.48 |  | 1.02 | 0.98 | 1.06 | 0.37 |  | 1.02 | 0.98 | 1.06 | 0.36 |
| rs8052560 | 16 | 88777242 | A | C | 1.02 | 0.98 | 1.05 | 0.34 |  | 1.03 | 0.99 | 1.08 | 0.11 |  | 0.96 | 0.90 | 1.02 | 0.17 |  | 1.02 | 0.97 | 1.07 | 0.35 |  | 0.98 | 0.95 | 1.02 | 0.37 |
| rs4640244 | 17 | 21284223 | A | G | 0.98 | 0.95 | 1.01 | 0.31 |  | 0.98 | 0.94 | 1.02 | 0.30 |  | 1.01 | 0.96 | 1.06 | 0.65 |  | 0.99 | 0.97 | 1.02 | 0.71 |  | 0.99 | 0.95 | 1.04 | 0.83 |
| rs3110496 | 17 | 27917771 | G | A | 1.00 | 0.98 | 1.02 | 0.93 |  | 1.01 | 0.98 | 1.05 | 0.47 |  | 0.97 | 0.93 | 1.02 | 0.21 |  | 0.99 | 0.94 | 1.03 | 0.54 |  | 1.01 | 0.98 | 1.04 | 0.40 |
| rs3764419 | 17 | 29164023 | C | A | 1.00 | 0.97 | 1.03 | 0.98 |  | 1.01 | 0.97 | 1.04 | 0.76 |  | 0.98 | 0.92 | 1.04 | 0.54 |  | 1.02 | 0.98 | 1.05 | 0.33 |  | 0.98 | 0.95 | 1.01 | 0.22 |
| rs17780086 | 17 | 30343282 | A | G | 1.03 | 0.99 | 1.06 | 0.15 |  | 1.02 | 0.98 | 1.06 | 0.27 |  | 1.04 | 0.94 | 1.15 | 0.42 |  | 1.05 | 1.00 | 1.10 | 0.05 |  | 1.00 | 0.96 | 1.04 | 0.96 |
| rs1043515 | 17 | 36922196 | G | A | 1.01 | 0.98 | 1.03 | 0.61 |  | 1.00 | 0.97 | 1.02 | 0.77 |  | 1.05 | 1.02 | 1.07 | <0.001 |  | 1.03 | 1.00 | 1.06 | 0.06 |  | 1.00 | 0.98 | 1.03 | 0.72 |
| rs4986172 | 17 | 43216281 | C | T | 1.01 | 0.97 | 1.05 | 0.55 |  | 1.02 | 0.98 | 1.06 | 0.37 |  | 1.00 | 0.94 | 1.06 | 0.98 |  | 0.99 | 0.96 | 1.03 | 0.66 |  | 1.03 | 0.97 | 1.09 | 0.35 |
| rs2072153 | 17 | 47390014 | C | G | 1.02 | 0.98 | 1.05 | 0.33 |  | 1.02 | 0.99 | 1.06 | 0.26 |  | 1.02 | 0.98 | 1.06 | 0.40 |  | 1.01 | 0.96 | 1.06 | 0.65 |  | 1.00 | 0.96 | 1.05 | 0.92 |
| rs4605213 | 17 | 49244747 | C | G | 1.01 | 0.97 | 1.05 | 0.58 |  | 1.01 | 0.97 | 1.05 | 0.68 |  | 1.01 | 0.98 | 1.05 | 0.42 |  | 1.04 | 1.00 | 1.08 | 0.04 |  | 0.99 | 0.95 | 1.03 | 0.67 |
| rs227724 | 17 | 54778817 | T | A | 1.02 | 0.97 | 1.07 | 0.41 |  | 1.02 | 0.97 | 1.08 | 0.42 |  | 1.00 | 0.95 | 1.04 | 0.88 |  | 1.01 | 0.95 | 1.07 | 0.77 |  | 1.02 | 0.97 | 1.07 | 0.54 |
| rs2079795 | 17 | 59496649 | T | C | 1.00 | 0.97 | 1.02 | 0.82 |  | 1.00 | 0.97 | 1.03 | 0.91 |  | 1.00 | 0.95 | 1.05 | 0.95 |  | 1.00 | 0.96 | 1.04 | 0.87 |  | 1.01 | 0.98 | 1.05 | 0.52 |
| rs2665838 | 17 | 61966465 | G | C | 0.99 | 0.97 | 1.02 | 0.60 |  | 0.98 | 0.95 | 1.01 | 0.14 |  | 1.06 | 1.00 | 1.12 | 0.06 |  | 1.00 | 0.97 | 1.03 | 1.00 |  | 1.00 | 0.96 | 1.05 | 0.95 |
| rs11867479 | 17 | 68090207 | T | C | 1.01 | 0.98 | 1.03 | 0.65 |  | 1.01 | 0.98 | 1.05 | 0.47 |  | 0.99 | 0.95 | 1.04 | 0.68 |  | 1.00 | 0.97 | 1.04 | 0.94 |  | 1.03 | 0.98 | 1.07 | 0.24 |
| rs4800452 | 18 | 20727611 | T | C | 1.02 | 0.99 | 1.05 | 0.17 |  | 1.03 | 0.99 | 1.06 | 0.12 |  | 1.05 | 0.98 | 1.11 | 0.16 |  | 1.03 | 0.99 | 1.07 | 0.15 |  | 1.04 | 1.00 | 1.08 | 0.07 |
| rs9967417 | 18 | 46959500 | G | C | 1.01 | 0.98 | 1.03 | 0.56 |  | 1.01 | 0.98 | 1.04 | 0.43 |  | 0.98 | 0.91 | 1.05 | 0.56 |  | 1.01 | 0.97 | 1.04 | 0.68 |  | 1.01 | 0.97 | 1.05 | 0.72 |
| rs17782313 | 18 | 57851097 | C | T | 0.99 | 0.95 | 1.03 | 0.64 |  | 0.98 | 0.94 | 1.02 | 0.31 |  | 1.02 | 0.95 | 1.09 | 0.57 |  | 0.98 | 0.94 | 1.03 | 0.50 |  | 0.99 | 0.94 | 1.05 | 0.84 |
| rs12982744 | 19 | 2177193 | G | C | 1.00 | 0.97 | 1.04 | 0.97 |  | 1.00 | 0.97 | 1.03 | 0.86 |  | 1.02 | 0.98 | 1.07 | 0.27 |  | 1.00 | 0.97 | 1.03 | 0.99 |  | 1.01 | 0.96 | 1.05 | 0.80 |
| rs7507204 | 19 | 3428834 | C | G | 1.02 | 0.98 | 1.05 | 0.37 |  | 1.01 | 0.97 | 1.06 | 0.52 |  | 1.00 | 0.93 | 1.07 | 0.97 |  | 1.01 | 0.95 | 1.07 | 0.85 |  | 1.02 | 0.98 | 1.07 | 0.23 |
| rs891088 | 19 | 7184762 | G | A | 1.03 | 1.00 | 1.06 | 0.08 |  | 1.03 | 1.00 | 1.06 | 0.07 |  | 1.01 | 0.94 | 1.08 | 0.84 |  | 1.04 | 0.99 | 1.09 | 0.16 |  | 1.01 | 0.97 | 1.05 | 0.61 |
| rs4072910 | 19 | 8644031 | G | C | 0.99 | 0.95 | 1.03 | 0.68 |  | 1.00 | 0.96 | 1.04 | 0.95 |  | 0.99 | 0.93 | 1.04 | 0.64 |  | 0.99 | 0.94 | 1.05 | 0.83 |  | 1.00 | 0.95 | 1.05 | 1.00 |
| rs2279008 | 19 | 17283303 | T | C | 1.01 | 0.99 | 1.03 | 0.29 |  | 0.99 | 0.96 | 1.02 | 0.71 |  | 1.04 | 0.99 | 1.10 | 0.09 |  | 1.01 | 0.97 | 1.05 | 0.70 |  | 1.02 | 1.00 | 1.05 | 0.06 |
| rs17318596 | 19 | 41937095 | A | G | 0.95 | 0.93 | 0.97 | <0.001 |  | 0.96 | 0.94 | 0.98 | 0.001 |  | 0.93 | 0.89 | 0.97 | <0.001 |  | 0.96 | 0.93 | 1.00 | 0.02 |  | 0.95 | 0.91 | 0.99 | 0.01 |
| rs1741344 | 20 | 4101800 | C | T | 0.97 | 0.95 | 1.00 | 0.04 |  | 0.99 | 0.94 | 1.03 | 0.57 |  | 0.94 | 0.90 | 0.99 | 0.02 |  | 0.96 | 0.93 | 1.00 | 0.05 |  | 0.97 | 0.93 | 1.00 | 0.05 |
| rs2145272 | 20 | 6626218 | G | A | 1.04 | 1.01 | 1.06 | 0.005 |  | 1.05 | 1.01 | 1.08 | 0.005 |  | 1.01 | 0.95 | 1.07 | 0.75 |  | 1.05 | 1.02 | 1.09 | 0.002 |  | 1.03 | 0.99 | 1.08 | 0.14 |
| rs7274811 | 20 | 32333181 | G | T | 1.01 | 0.97 | 1.04 | 0.68 |  | 1.01 | 0.98 | 1.04 | 0.59 |  | 0.99 | 0.93 | 1.04 | 0.58 |  | 1.00 | 0.96 | 1.04 | 0.91 |  | 1.00 | 0.95 | 1.05 | 0.94 |
| rs143384 | 20 | 34025756 | G | A | 0.98 | 0.95 | 1.01 | 0.19 |  | 0.98 | 0.94 | 1.02 | 0.24 |  | 0.97 | 0.92 | 1.02 | 0.20 |  | 1.00 | 0.95 | 1.04 | 0.85 |  | 0.95 | 0.92 | 0.98 | 0.001 |
| rs237743 | 20 | 47903019 | A | G | 0.98 | 0.96 | 1.01 | 0.27 |  | 1.00 | 0.96 | 1.03 | 0.84 |  | 0.93 | 0.90 | 0.97 | 0.002 |  | 0.98 | 0.94 | 1.03 | 0.46 |  | 0.97 | 0.93 | 1.01 | 0.15 |
| rs2834442 | 21 | 35690786 | A | T | 1.01 | 0.97 | 1.05 | 0.52 |  | 1.01 | 0.98 | 1.05 | 0.49 |  | 0.99 | 0.92 | 1.07 | 0.79 |  | 1.02 | 0.98 | 1.06 | 0.31 |  | 1.02 | 0.97 | 1.07 | 0.45 |
| rs4821083 | 22 | 33056341 | G | A | 1.00 | 0.96 | 1.03 | 0.81 |  | 1.03 | 0.99 | 1.07 | 0.18 |  | 0.94 | 0.88 | 1.01 | 0.08 |  | 1.01 | 0.96 | 1.07 | 0.61 |  | 1.01 | 0.98 | 1.05 | 0.56 |

*Odds ratios are adjusted for the first 8 principal components of population stratification. All coefficeints reflect effect of one height increasing allele.*

**Supplementary Table 5: Association of 32 individual BMI SNPs with prostate cancer risk. Odds-ratios indicate the effect of one extra BMI increasing allele.**

|  |  |  | Alleles | | Any prostate cancer (N=41,062) | | | |  | Localised prostate cancer (N=33,189) | | | |  | Advanced prostate cancer (N=24,539) | | | |  | Low grade prostate cancer (N=28,998) | | | |  | High grade prostate cancer (N=28,444) | | | |
| --- | --- | --- | --- | --- | --- | --- | --- | --- | --- | --- | --- | --- | --- | --- | --- | --- | --- | --- | --- | --- | --- | --- | --- | --- | --- | --- | --- | --- |
|  |  |  | BMI |  | Odds | Confidence interval | | p- |  | Odds | Confidence interval | | p- |  | Odds | Confidence interval | | p- |  | Odds | Confidence interval | | p- |  | Odds | Confidence interval | | p- |
| Rs ID | Chr. | Position | increasing | Other | ratio | Lower | Upper | value |  | ratio | Lower | Upper | value |  | ratio | Lower | Upper | value |  | ratio | Lower | Upper | value |  | ratio | Lower | Upper | value |
| rs2815752 | 1 | 72812440 | A | G | 0.98 | 0.94 | 1.02 | 0.33 |  | 0.97 | 0.94 | 1.01 | 0.13 |  | 0.97 | 0.90 | 1.04 | 0.43 |  | 0.98 | 0.94 | 1.02 | 0.34 |  | 0.98 | 0.94 | 1.01 | 0.24 |
| rs1514175 | 1 | 74991644 | A | G | 1.01 | 0.98 | 1.03 | 0.69 |  | 1.00 | 0.97 | 1.04 | 0.90 |  | 0.99 | 0.97 | 1.02 | 0.57 |  | 1.01 | 0.98 | 1.04 | 0.63 |  | 0.98 | 0.95 | 1.02 | 0.36 |
| rs1555543 | 1 | 96944797 | C | A | 0.99 | 0.96 | 1.03 | 0.75 |  | 1.01 | 0.96 | 1.05 | 0.78 |  | 0.95 | 0.91 | 0.99 | 0.01 |  | 0.98 | 0.95 | 1.02 | 0.38 |  | 1.00 | 0.95 | 1.04 | 0.83 |
| rs543874 | 1 | 177889480 | G | A | 1.01 | 0.99 | 1.04 | 0.34 |  | 1.03 | 0.99 | 1.06 | 0.11 |  | 1.04 | 0.98 | 1.09 | 0.22 |  | 1.02 | 0.98 | 1.05 | 0.35 |  | 1.01 | 0.97 | 1.05 | 0.61 |
| rs2867125 | 2 | 622827 | C | T | 0.96 | 0.92 | 1.01 | 0.15 |  | 0.95 | 0.89 | 1.01 | 0.08 |  | 1.04 | 0.99 | 1.09 | 0.16 |  | 0.94 | 0.88 | 1.01 | 0.07 |  | 1.02 | 0.98 | 1.06 | 0.29 |
| rs713586 | 2 | 25158008 | C | T | 1.01 | 0.98 | 1.04 | 0.45 |  | 1.01 | 0.98 | 1.04 | 0.57 |  | 1.00 | 0.96 | 1.05 | 0.97 |  | 1.03 | 1.00 | 1.07 | 0.07 |  | 0.98 | 0.95 | 1.01 | 0.21 |
| rs887912 | 2 | 59302877 | T | C | 1.02 | 0.99 | 1.04 | 0.27 |  | 1.02 | 0.98 | 1.05 | 0.41 |  | 1.01 | 0.96 | 1.07 | 0.61 |  | 1.03 | 0.99 | 1.07 | 0.11 |  | 1.01 | 0.97 | 1.04 | 0.63 |
| rs2890652 | 2 | 142959931 | C | T | 1.01 | 0.97 | 1.05 | 0.80 |  | 1.00 | 0.94 | 1.07 | 0.93 |  | 1.04 | 1.00 | 1.09 | 0.06 |  | 1.00 | 0.95 | 1.06 | 1.00 |  | 0.99 | 0.95 | 1.03 | 0.58 |
| rs13078807 | 3 | 85884150 | G | A | 0.98 | 0.95 | 1.01 | 0.18 |  | 0.97 | 0.94 | 1.01 | 0.12 |  | 1.03 | 0.98 | 1.07 | 0.27 |  | 0.97 | 0.93 | 1.01 | 0.13 |  | 1.00 | 0.96 | 1.04 | 0.90 |
| rs9816226 | 3 | 185834499 | T | A | 0.98 | 0.93 | 1.03 | 0.46 |  | 1.00 | 0.95 | 1.06 | 0.90 |  | 0.98 | 0.90 | 1.07 | 0.60 |  | 0.95 | 0.89 | 1.02 | 0.16 |  | 1.01 | 0.96 | 1.07 | 0.68 |
| rs10938397 | 4 | 45182527 | G | A | 1.00 | 0.98 | 1.02 | 0.73 |  | 1.01 | 0.99 | 1.03 | 0.35 |  | 0.99 | 0.95 | 1.02 | 0.46 |  | 1.01 | 0.98 | 1.04 | 0.57 |  | 1.01 | 0.99 | 1.04 | 0.29 |
| rs13107325 | 4 | 103188709 | T | C | 1.02 | 0.96 | 1.08 | 0.55 |  | 1.02 | 0.95 | 1.09 | 0.62 |  | 1.01 | 0.92 | 1.11 | 0.87 |  | 1.03 | 0.94 | 1.14 | 0.52 |  | 1.04 | 0.97 | 1.11 | 0.27 |
| rs2112347 | 5 | 75015242 | T | G | 0.96 | 0.94 | 0.98 | <0.001 |  | 0.96 | 0.94 | 0.99 | 0.008 |  | 0.95 | 0.92 | 0.99 | 0.02 |  | 0.96 | 0.93 | 1.00 | 0.03 |  | 0.96 | 0.93 | 0.99 | 0.006 |
| rs4836133 | 5 | 124332103 | A | C | 1.00 | 0.98 | 1.03 | 0.80 |  | 1.00 | 0.97 | 1.02 | 0.71 |  | 1.04 | 1.01 | 1.07 | 0.01 |  | 1.01 | 0.98 | 1.03 | 0.51 |  | 1.01 | 0.98 | 1.05 | 0.57 |
| rs206936 | 6 | 34302869 | G | A | 1.02 | 0.98 | 1.06 | 0.37 |  | 1.02 | 0.98 | 1.07 | 0.32 |  | 1.04 | 1.00 | 1.10 | 0.08 |  | 1.00 | 0.96 | 1.04 | 0.92 |  | 1.03 | 0.99 | 1.07 | 0.20 |
| rs987237 | 6 | 50803050 | G | A | 0.99 | 0.96 | 1.02 | 0.59 |  | 1.01 | 0.98 | 1.04 | 0.69 |  | 0.99 | 0.95 | 1.02 | 0.38 |  | 0.99 | 0.94 | 1.04 | 0.66 |  | 1.00 | 0.96 | 1.04 | 0.96 |
| rs10968576 | 9 | 28414339 | G | A | 1.00 | 0.96 | 1.05 | 0.98 |  | 1.00 | 0.95 | 1.05 | 0.91 |  | 0.99 | 0.94 | 1.05 | 0.74 |  | 1.00 | 0.96 | 1.05 | 0.97 |  | 0.98 | 0.93 | 1.04 | 0.56 |
| rs4929949 | 11 | 8604593 | C | T | 1.01 | 0.99 | 1.04 | 0.27 |  | 1.01 | 0.98 | 1.04 | 0.44 |  | 0.99 | 0.95 | 1.03 | 0.56 |  | 1.02 | 0.98 | 1.05 | 0.35 |  | 1.00 | 0.97 | 1.03 | 0.98 |
| rs10767664 | 11 | 27725986 | A | T | 1.03 | 1.00 | 1.06 | 0.08 |  | 1.03 | 1.00 | 1.07 | 0.03 |  | 1.05 | 0.98 | 1.13 | 0.16 |  | 1.05 | 1.01 | 1.08 | 0.008 |  | 1.03 | 0.98 | 1.08 | 0.28 |
| rs3817334 | 11 | 47650993 | T | C | 0.98 | 0.95 | 1.01 | 0.12 |  | 0.97 | 0.94 | 1.00 | 0.02 |  | 0.99 | 0.95 | 1.04 | 0.72 |  | 0.96 | 0.93 | 1.00 | 0.04 |  | 0.98 | 0.95 | 1.02 | 0.38 |
| rs7138803 | 12 | 50247468 | A | G | 1.03 | 1.00 | 1.06 | 0.09 |  | 1.03 | 1.00 | 1.05 | 0.02 |  | 1.05 | 0.99 | 1.11 | 0.11 |  | 1.02 | 0.98 | 1.05 | 0.37 |  | 1.05 | 1.01 | 1.08 | 0.01 |
| rs4771122 | 13 | 28020180 | G | A | 0.98 | 0.94 | 1.02 | 0.37 |  | 0.98 | 0.94 | 1.03 | 0.44 |  | 1.00 | 0.95 | 1.06 | 0.90 |  | 0.98 | 0.94 | 1.02 | 0.29 |  | 1.00 | 0.94 | 1.06 | 0.92 |
| rs11847697 | 14 | 30515112 | T | C | 1.03 | 0.98 | 1.08 | 0.22 |  | 1.04 | 0.98 | 1.11 | 0.22 |  | 1.02 | 0.95 | 1.11 | 0.56 |  | 1.01 | 0.94 | 1.08 | 0.80 |  | 1.06 | 0.99 | 1.13 | 0.11 |
| rs10150332 | 14 | 79936964 | C | T | 1.00 | 0.97 | 1.04 | 0.80 |  | 1.00 | 0.97 | 1.04 | 0.99 |  | 1.01 | 0.96 | 1.07 | 0.69 |  | 1.02 | 0.98 | 1.06 | 0.39 |  | 0.99 | 0.96 | 1.03 | 0.75 |
| rs2241423 | 15 | 68086838 | G | A | 0.97 | 0.93 | 1.00 | 0.08 |  | 0.96 | 0.92 | 1.00 | 0.04 |  | 0.94 | 0.89 | 0.99 | 0.03 |  | 0.99 | 0.95 | 1.05 | 0.82 |  | 0.95 | 0.92 | 0.98 | 0.003 |
| rs12444979 | 16 | 19933600 | C | T | 0.97 | 0.94 | 1.01 | 0.18 |  | 0.97 | 0.93 | 1.02 | 0.21 |  | 0.98 | 0.94 | 1.03 | 0.38 |  | 0.95 | 0.91 | 1.00 | 0.06 |  | 1.00 | 0.95 | 1.06 | 0.96 |
| rs7359397 | 16 | 28885659 | T | C | 1.00 | 0.97 | 1.02 | 0.76 |  | 0.99 | 0.96 | 1.03 | 0.74 |  | 1.00 | 0.97 | 1.04 | 0.88 |  | 1.00 | 0.97 | 1.04 | 1.00 |  | 0.99 | 0.95 | 1.03 | 0.52 |
| rs1558902 | 16 | 53803574 | A | T | 0.97 | 0.94 | 1.00 | 0.10 |  | 0.97 | 0.93 | 1.01 | 0.13 |  | 1.00 | 0.95 | 1.05 | 0.85 |  | 0.95 | 0.90 | 1.00 | 0.06 |  | 0.99 | 0.96 | 1.02 | 0.41 |
| rs571312 | 18 | 57839769 | A | C | 0.99 | 0.95 | 1.04 | 0.75 |  | 0.98 | 0.94 | 1.03 | 0.39 |  | 1.02 | 0.96 | 1.09 | 0.55 |  | 0.99 | 0.94 | 1.04 | 0.63 |  | 1.00 | 0.94 | 1.05 | 0.91 |
| rs29941 | 19 | 34309532 | G | A | 1.02 | 0.98 | 1.05 | 0.43 |  | 1.02 | 0.97 | 1.07 | 0.49 |  | 0.98 | 0.93 | 1.03 | 0.45 |  | 1.01 | 0.95 | 1.08 | 0.76 |  | 1.02 | 0.98 | 1.07 | 0.37 |
| rs2287019 | 19 | 46202172 | C | T | 1.01 | 0.98 | 1.04 | 0.40 |  | 1.03 | 1.00 | 1.05 | 0.07 |  | 1.01 | 0.95 | 1.06 | 0.82 |  | 1.04 | 0.99 | 1.09 | 0.15 |  | 1.01 | 0.97 | 1.05 | 0.69 |
| rs3810291 | 19 | 47569003 | A | G | 0.99 | 0.95 | 1.02 | 0.47 |  | 0.98 | 0.95 | 1.02 | 0.33 |  | 0.98 | 0.93 | 1.03 | 0.43 |  | 0.97 | 0.93 | 1.02 | 0.27 |  | 0.99 | 0.96 | 1.02 | 0.53 |

*Odds ratios are adjusted for the first 8 principal components of population stratification.*

**Supplementary Table 6: Association of 179 individual height SNPs with all-cause mortality after prostate cancer diagnosis. Hazard ratios indicate the effect of one extra height increasing allele.**

|  |  |  | Alleles | | Any prostate cancer (N=15,491) | | | | Localised prostate cancer (N=9,100) | | | | Advanced prostate cancer (N=3,685) | | | | Low grade prostate cancer (N=6,026) | | | | High grade prostate cancer (N=6,301) | | | |
| --- | --- | --- | --- | --- | --- | --- | --- | --- | --- | --- | --- | --- | --- | --- | --- | --- | --- | --- | --- | --- | --- | --- | --- | --- |
|  |  |  | Height |  | Hazard | Confidence interval | | p- | Hazard | Confidence interval | | p- | Hazard | Confidence interval | | p- | Hazard | Confidence interval | | p- | Hazard | Confidence interval | | p- |
| Rs ID | Chr. | Position | increasing | Other | ratio | Lower | Upper | value | ratio | Lower | Upper | value | ratio | Lower | Upper | value | ratio | Lower | Upper | value | ratio | Lower | Upper | value |
| rs425277 | 1 | 2069172 | T | C | 0.98 | 0.92 | 1.04 | 0.51 | 0.97 | 0.90 | 1.05 | 0.47 | 0.98 | 0.93 | 1.05 | 0.63 | 0.99 | 0.87 | 1.13 | 0.89 | 0.98 | 0.92 | 1.04 | 0.51 |
| rs2284746 | 1 | 17306675 | G | C | 0.99 | 0.94 | 1.06 | 0.87 | 1.00 | 0.94 | 1.07 | 0.94 | 1.02 | 0.93 | 1.12 | 0.68 | 1.03 | 0.97 | 1.09 | 0.36 | 1.04 | 0.95 | 1.13 | 0.39 |
| rs1738475 | 1 | 23536891 | C | G | 0.99 | 0.93 | 1.05 | 0.65 | 0.97 | 0.90 | 1.05 | 0.47 | 1.01 | 0.96 | 1.07 | 0.71 | 0.97 | 0.90 | 1.05 | 0.44 | 1.01 | 0.93 | 1.10 | 0.87 |
| rs4601530 | 1 | 25044111 | C | T | 1.02 | 0.96 | 1.08 | 0.52 | 1.05 | 0.94 | 1.18 | 0.38 | 0.98 | 0.89 | 1.08 | 0.69 | 1.12 | 0.96 | 1.30 | 0.16 | 0.97 | 0.87 | 1.08 | 0.57 |
| rs7532866 | 1 | 26741544 | A | G | 0.98 | 0.94 | 1.02 | 0.34 | 0.97 | 0.92 | 1.02 | 0.20 | 1.00 | 0.94 | 1.06 | 0.92 | 0.98 | 0.92 | 1.05 | 0.57 | 0.99 | 0.92 | 1.06 | 0.71 |
| rs2154319 | 1 | 41745770 | C | T | 0.97 | 0.93 | 1.01 | 0.10 | 0.94 | 0.88 | 1.00 | 0.06 | 0.99 | 0.92 | 1.06 | 0.69 | 0.99 | 0.93 | 1.05 | 0.68 | 0.93 | 0.86 | 1.01 | 0.07 |
| rs17391694 | 1 | 78623626 | T | C | 1.00 | 0.94 | 1.07 | 0.99 | 1.05 | 0.97 | 1.14 | 0.24 | 1.01 | 0.91 | 1.12 | 0.90 | 1.07 | 0.90 | 1.28 | 0.42 | 0.93 | 0.86 | 1.00 | 0.06 |
| rs6699417 | 1 | 89123443 | T | C | 0.96 | 0.93 | 0.99 | 0.007 | 0.93 | 0.87 | 1.00 | 0.04 | 1.02 | 0.97 | 1.07 | 0.41 | 0.93 | 0.90 | 0.97 | 0.001 | 0.94 | 0.89 | 1.00 | 0.03 |
| rs10874746 | 1 | 93323971 | C | T | 1.01 | 0.97 | 1.04 | 0.77 | 1.05 | 0.98 | 1.12 | 0.15 | 0.94 | 0.90 | 0.98 | 0.005 | 1.06 | 1.02 | 1.11 | 0.005 | 0.98 | 0.95 | 1.01 | 0.25 |
| rs9428104 | 1 | 118855587 | G | A | 1.01 | 0.97 | 1.04 | 0.73 | 1.03 | 0.96 | 1.09 | 0.46 | 1.00 | 0.97 | 1.03 | 0.77 | 1.05 | 0.95 | 1.16 | 0.33 | 1.00 | 0.95 | 1.05 | 1.00 |
| rs11205277 | 1 | 149892872 | G | A | 1.01 | 0.98 | 1.04 | 0.47 | 1.02 | 0.96 | 1.09 | 0.52 | 1.02 | 0.97 | 1.07 | 0.36 | 1.05 | 0.94 | 1.17 | 0.36 | 0.94 | 0.89 | 1.00 | 0.05 |
| rs17346452 | 1 | 172053287 | C | T | 1.05 | 0.99 | 1.10 | 0.11 | 1.02 | 0.93 | 1.12 | 0.69 | 1.07 | 1.00 | 1.14 | 0.05 | 1.04 | 0.92 | 1.17 | 0.54 | 1.05 | 0.98 | 1.12 | 0.21 |
| rs1325598 | 1 | 176792249 | G | A | 1.00 | 0.98 | 1.02 | 0.99 | 1.02 | 0.96 | 1.09 | 0.51 | 1.03 | 0.98 | 1.08 | 0.24 | 1.03 | 0.98 | 1.08 | 0.29 | 0.96 | 0.91 | 1.01 | 0.11 |
| rs1046934 | 1 | 184023529 | C | A | 1.03 | 0.99 | 1.07 | 0.19 | 1.05 | 1.00 | 1.11 | 0.07 | 0.97 | 0.92 | 1.03 | 0.33 | 1.00 | 0.95 | 1.05 | 0.98 | 1.03 | 0.95 | 1.11 | 0.49 |
| rs10863936 | 1 | 212237798 | G | A | 1.02 | 0.98 | 1.05 | 0.29 | 1.00 | 0.96 | 1.05 | 0.86 | 1.02 | 0.98 | 1.06 | 0.40 | 1.03 | 0.99 | 1.08 | 0.14 | 1.01 | 0.96 | 1.06 | 0.80 |
| rs6684205 | 1 | 218609702 | G | A | 1.04 | 0.98 | 1.11 | 0.20 | 1.01 | 0.94 | 1.07 | 0.86 | 1.05 | 0.96 | 1.14 | 0.29 | 0.99 | 0.93 | 1.05 | 0.72 | 1.01 | 0.95 | 1.09 | 0.67 |
| rs11118346 | 1 | 219743719 | C | T | 0.99 | 0.96 | 1.03 | 0.67 | 0.98 | 0.93 | 1.04 | 0.51 | 1.02 | 0.97 | 1.08 | 0.46 | 0.98 | 0.95 | 1.01 | 0.28 | 0.98 | 0.93 | 1.04 | 0.56 |
| rs10799445 | 1 | 227911883 | A | C | 1.01 | 0.97 | 1.06 | 0.58 | 1.01 | 0.95 | 1.08 | 0.71 | 1.02 | 0.95 | 1.11 | 0.55 | 0.97 | 0.89 | 1.07 | 0.56 | 1.06 | 1.01 | 1.12 | 0.03 |
| rs4665736 | 2 | 25187599 | T | C | 0.96 | 0.91 | 1.00 | 0.07 | 0.92 | 0.84 | 1.01 | 0.07 | 0.97 | 0.91 | 1.04 | 0.44 | 0.94 | 0.83 | 1.06 | 0.31 | 0.96 | 0.92 | 1.01 | 0.14 |
| rs6714546 | 2 | 33361425 | G | A | 1.01 | 0.96 | 1.06 | 0.66 | 1.06 | 0.98 | 1.15 | 0.16 | 1.03 | 0.97 | 1.10 | 0.31 | 1.06 | 0.98 | 1.15 | 0.12 | 1.09 | 1.01 | 1.18 | 0.02 |
| rs17511102 | 2 | 37960613 | T | A | 1.01 | 0.94 | 1.07 | 0.86 | 0.98 | 0.84 | 1.13 | 0.74 | 1.06 | 0.99 | 1.13 | 0.10 | 0.90 | 0.74 | 1.09 | 0.29 | 1.17 | 1.00 | 1.38 | 0.06 |
| rs2341459 | 2 | 44768202 | T | C | 1.03 | 0.99 | 1.07 | 0.14 | 1.04 | 0.96 | 1.14 | 0.31 | 1.06 | 1.01 | 1.10 | 0.01 | 1.01 | 0.90 | 1.14 | 0.85 | 1.04 | 0.99 | 1.09 | 0.13 |
| rs12474201 | 2 | 46921285 | A | G | 0.95 | 0.91 | 0.99 | 0.009 | 0.95 | 0.89 | 1.01 | 0.10 | 0.95 | 0.90 | 1.01 | 0.13 | 0.95 | 0.86 | 1.04 | 0.27 | 0.92 | 0.87 | 0.96 | <0.001 |
| rs3791675 | 2 | 56111309 | C | T | 0.97 | 0.92 | 1.03 | 0.36 | 0.98 | 0.90 | 1.07 | 0.71 | 0.97 | 0.90 | 1.04 | 0.35 | 0.94 | 0.88 | 1.02 | 0.12 | 1.02 | 0.95 | 1.10 | 0.60 |
| rs11684404 | 2 | 88924622 | C | T | 1.03 | 0.97 | 1.10 | 0.28 | 1.03 | 0.96 | 1.12 | 0.41 | 0.99 | 0.92 | 1.07 | 0.79 | 1.11 | 1.01 | 1.21 | 0.03 | 1.02 | 0.95 | 1.08 | 0.63 |
| rs7567288 | 2 | 134434824 | C | T | 0.98 | 0.92 | 1.03 | 0.38 | 1.02 | 0.96 | 1.08 | 0.50 | 0.99 | 0.93 | 1.06 | 0.77 | 0.95 | 0.87 | 1.05 | 0.32 | 1.05 | 1.00 | 1.10 | 0.07 |
| rs7567851 | 2 | 178684720 | C | G | 1.00 | 0.95 | 1.06 | 0.98 | 0.96 | 0.89 | 1.04 | 0.30 | 1.10 | 1.05 | 1.15 | <0.001 | 0.99 | 0.91 | 1.08 | 0.80 | 0.97 | 0.90 | 1.05 | 0.50 |
| rs1351164 | 2 | 218271898 | T | C | 0.98 | 0.92 | 1.04 | 0.50 | 1.00 | 0.95 | 1.06 | 0.89 | 0.99 | 0.90 | 1.08 | 0.82 | 1.05 | 0.95 | 1.16 | 0.32 | 0.99 | 0.88 | 1.11 | 0.84 |
| rs12470505 | 2 | 219908369 | T | G | 1.06 | 1.00 | 1.13 | 0.06 | 1.00 | 0.89 | 1.12 | 0.97 | 1.21 | 1.13 | 1.30 | <0.001 | 1.03 | 0.92 | 1.16 | 0.59 | 1.08 | 0.96 | 1.22 | 0.21 |
| rs2629046 | 2 | 225047744 | T | C | 1.04 | 1.00 | 1.07 | 0.04 | 1.08 | 1.01 | 1.15 | 0.03 | 0.99 | 0.93 | 1.06 | 0.79 | 1.04 | 0.98 | 1.11 | 0.15 | 1.00 | 0.94 | 1.06 | 0.93 |
| rs2580816 | 2 | 232797966 | C | T | 1.02 | 0.98 | 1.06 | 0.43 | 1.04 | 0.98 | 1.10 | 0.17 | 0.95 | 0.89 | 1.03 | 0.21 | 1.01 | 0.89 | 1.14 | 0.88 | 0.98 | 0.92 | 1.04 | 0.50 |
| rs12694997 | 2 | 242262986 | G | A | 0.99 | 0.94 | 1.04 | 0.64 | 1.03 | 0.96 | 1.11 | 0.41 | 0.98 | 0.91 | 1.05 | 0.56 | 1.02 | 0.89 | 1.15 | 0.82 | 1.01 | 0.90 | 1.13 | 0.89 |
| rs2597513 | 3 | 13555836 | C | T | 0.91 | 0.85 | 0.98 | 0.008 | 0.97 | 0.84 | 1.12 | 0.68 | 0.91 | 0.82 | 1.01 | 0.07 | 0.87 | 0.78 | 0.98 | 0.02 | 0.96 | 0.90 | 1.02 | 0.21 |
| rs13088462 | 3 | 51071713 | C | T | 1.01 | 0.91 | 1.11 | 0.89 | 1.02 | 0.88 | 1.18 | 0.82 | 0.99 | 0.91 | 1.08 | 0.82 | 1.02 | 0.84 | 1.24 | 0.83 | 1.02 | 0.94 | 1.09 | 0.68 |
| rs2336725 | 3 | 53118739 | C | T | 1.04 | 1.00 | 1.08 | 0.03 | 1.06 | 1.02 | 1.10 | 0.002 | 1.05 | 1.00 | 1.10 | 0.04 | 1.07 | 0.99 | 1.16 | 0.11 | 1.05 | 1.01 | 1.10 | 0.02 |
| rs9835332 | 3 | 56667682 | G | C | 1.00 | 0.96 | 1.05 | 0.88 | 0.98 | 0.94 | 1.02 | 0.32 | 1.01 | 0.96 | 1.05 | 0.77 | 0.99 | 0.90 | 1.09 | 0.78 | 1.01 | 0.94 | 1.09 | 0.75 |
| rs17806888 | 3 | 67416322 | T | C | 1.03 | 0.93 | 1.15 | 0.51 | 1.11 | 0.94 | 1.31 | 0.21 | 1.03 | 0.95 | 1.11 | 0.51 | 1.08 | 0.97 | 1.20 | 0.17 | 1.04 | 0.91 | 1.19 | 0.56 |
| rs9863706 | 3 | 72437413 | C | T | 1.03 | 0.97 | 1.09 | 0.31 | 1.05 | 0.95 | 1.17 | 0.32 | 1.01 | 0.93 | 1.11 | 0.80 | 1.02 | 0.91 | 1.15 | 0.68 | 1.00 | 0.91 | 1.10 | 0.98 |
| rs6439167 | 3 | 129050756 | C | T | 0.91 | 0.86 | 0.96 | <0.001 | 0.92 | 0.86 | 0.98 | 0.008 | 0.92 | 0.86 | 0.99 | 0.02 | 0.90 | 0.80 | 1.02 | 0.09 | 0.93 | 0.85 | 1.02 | 0.13 |
| rs9844666 | 3 | 135974216 | G | A | 0.98 | 0.95 | 1.02 | 0.37 | 0.97 | 0.92 | 1.03 | 0.33 | 1.01 | 0.96 | 1.05 | 0.81 | 0.96 | 0.89 | 1.03 | 0.24 | 1.06 | 0.97 | 1.14 | 0.19 |
| rs724016 | 3 | 141105570 | G | A | 0.97 | 0.95 | 0.99 | 0.005 | 0.99 | 0.94 | 1.05 | 0.83 | 0.98 | 0.93 | 1.04 | 0.47 | 0.97 | 0.92 | 1.03 | 0.27 | 0.98 | 0.91 | 1.06 | 0.60 |
| rs572169 | 3 | 172165727 | T | C | 1.01 | 0.96 | 1.05 | 0.81 | 1.02 | 0.94 | 1.10 | 0.66 | 1.01 | 0.96 | 1.06 | 0.66 | 1.04 | 0.93 | 1.16 | 0.51 | 0.94 | 0.90 | 0.98 | 0.007 |
| rs720390 | 3 | 185548683 | A | G | 1.03 | 0.99 | 1.08 | 0.16 | 1.06 | 0.98 | 1.15 | 0.12 | 1.02 | 0.97 | 1.07 | 0.50 | 1.12 | 1.05 | 1.20 | 0.001 | 0.98 | 0.92 | 1.05 | 0.55 |
| rs2247341 | 4 | 1701317 | A | G | 1.03 | 0.99 | 1.08 | 0.11 | 1.00 | 0.93 | 1.07 | 0.96 | 1.06 | 1.00 | 1.13 | 0.05 | 0.97 | 0.89 | 1.06 | 0.54 | 1.09 | 1.05 | 1.14 | <0.001 |
| rs6449353 | 4 | 18033488 | T | C | 0.99 | 0.92 | 1.06 | 0.79 | 1.04 | 0.89 | 1.21 | 0.64 | 0.96 | 0.88 | 1.03 | 0.25 | 0.97 | 0.86 | 1.10 | 0.65 | 1.02 | 0.94 | 1.10 | 0.66 |
| rs17081935 | 4 | 57823476 | T | C | 1.01 | 0.97 | 1.06 | 0.60 | 1.02 | 0.95 | 1.10 | 0.63 | 1.03 | 0.96 | 1.11 | 0.37 | 1.00 | 0.88 | 1.15 | 0.94 | 0.99 | 0.91 | 1.08 | 0.85 |
| rs7697556 | 4 | 73515313 | T | C | 0.96 | 0.94 | 0.99 | 0.005 | 1.00 | 0.93 | 1.07 | 0.98 | 0.92 | 0.88 | 0.97 | 0.004 | 0.96 | 0.89 | 1.03 | 0.27 | 0.97 | 0.94 | 1.01 | 0.15 |
| rs788867 | 4 | 82150006 | G | T | 1.01 | 0.94 | 1.08 | 0.86 | 1.02 | 0.91 | 1.15 | 0.71 | 1.03 | 0.96 | 1.10 | 0.44 | 1.09 | 0.96 | 1.23 | 0.19 | 0.96 | 0.89 | 1.04 | 0.31 |
| rs10010325 | 4 | 106106353 | A | C | 1.00 | 0.93 | 1.07 | 0.93 | 1.01 | 0.91 | 1.11 | 0.89 | 1.00 | 0.94 | 1.07 | 0.91 | 1.12 | 1.04 | 1.21 | 0.002 | 0.96 | 0.86 | 1.06 | 0.40 |
| rs7689420 | 4 | 145568352 | C | T | 1.10 | 1.02 | 1.19 | 0.02 | 1.14 | 1.07 | 1.21 | <0.001 | 1.11 | 1.01 | 1.22 | 0.03 | 1.11 | 0.97 | 1.26 | 0.12 | 1.16 | 1.04 | 1.29 | 0.006 |
| rs955748 | 4 | 184215675 | G | A | 1.00 | 0.94 | 1.08 | 0.90 | 1.07 | 1.00 | 1.14 | 0.05 | 1.00 | 0.91 | 1.10 | 0.98 | 1.03 | 0.94 | 1.13 | 0.48 | 1.04 | 0.96 | 1.13 | 0.34 |
| rs1173727 | 5 | 32830521 | T | C | 0.97 | 0.95 | 1.00 | 0.06 | 0.97 | 0.93 | 1.01 | 0.10 | 1.05 | 0.98 | 1.13 | 0.17 | 0.99 | 0.87 | 1.12 | 0.88 | 0.98 | 0.93 | 1.03 | 0.37 |
| rs11958779 | 5 | 55001899 | G | A | 1.01 | 0.97 | 1.04 | 0.70 | 0.99 | 0.93 | 1.06 | 0.77 | 0.98 | 0.93 | 1.03 | 0.49 | 0.94 | 0.87 | 1.02 | 0.12 | 1.02 | 0.97 | 1.07 | 0.36 |
| rs10037512 | 5 | 88354675 | T | C | 0.98 | 0.94 | 1.03 | 0.43 | 0.96 | 0.88 | 1.04 | 0.28 | 1.02 | 0.97 | 1.08 | 0.46 | 0.95 | 0.88 | 1.04 | 0.28 | 0.99 | 0.92 | 1.06 | 0.69 |
| rs13177718 | 5 | 108113344 | C | T | 1.02 | 0.93 | 1.12 | 0.72 | 1.02 | 0.87 | 1.20 | 0.78 | 1.02 | 0.93 | 1.11 | 0.69 | 1.05 | 0.81 | 1.36 | 0.71 | 1.04 | 0.93 | 1.15 | 0.52 |
| rs1582931 | 5 | 122657199 | G | A | 0.97 | 0.93 | 1.02 | 0.21 | 0.98 | 0.89 | 1.08 | 0.63 | 1.04 | 1.00 | 1.09 | 0.07 | 0.92 | 0.87 | 0.97 | 0.003 | 1.00 | 0.93 | 1.08 | 0.93 |
| rs274546 | 5 | 131699867 | G | A | 0.96 | 0.92 | 1.00 | 0.06 | 0.94 | 0.86 | 1.03 | 0.19 | 0.99 | 0.90 | 1.10 | 0.89 | 0.99 | 0.95 | 1.04 | 0.73 | 0.95 | 0.92 | 0.98 | 0.004 |
| rs526896 | 5 | 134356705 | T | G | 1.00 | 0.96 | 1.04 | 0.95 | 1.02 | 0.95 | 1.09 | 0.66 | 0.99 | 0.95 | 1.04 | 0.68 | 1.06 | 1.00 | 1.11 | 0.05 | 0.95 | 0.89 | 1.00 | 0.05 |
| rs4282339 | 5 | 168256240 | G | A | 1.04 | 0.99 | 1.09 | 0.09 | 1.03 | 1.00 | 1.07 | 0.07 | 1.00 | 0.92 | 1.08 | 0.96 | 1.03 | 0.95 | 1.12 | 0.48 | 1.04 | 0.96 | 1.14 | 0.31 |
| rs12153391 | 5 | 171203438 | C | A | 0.93 | 0.89 | 0.96 | <0.001 | 0.96 | 0.87 | 1.06 | 0.42 | 0.89 | 0.80 | 0.99 | 0.03 | 0.94 | 0.88 | 1.00 | 0.06 | 0.91 | 0.83 | 0.99 | 0.02 |
| rs889014 | 5 | 172984114 | C | T | 1.00 | 0.97 | 1.03 | 0.97 | 0.97 | 0.91 | 1.03 | 0.32 | 1.05 | 1.01 | 1.10 | 0.01 | 0.95 | 0.90 | 1.01 | 0.10 | 0.99 | 0.94 | 1.03 | 0.55 |
| rs422421 | 5 | 176517326 | C | T | 1.01 | 0.96 | 1.05 | 0.72 | 1.03 | 0.94 | 1.13 | 0.52 | 1.01 | 0.96 | 1.06 | 0.64 | 0.99 | 0.91 | 1.08 | 0.84 | 1.08 | 1.00 | 1.16 | 0.05 |
| rs6879260 | 5 | 179731014 | C | T | 1.00 | 0.94 | 1.05 | 0.85 | 1.01 | 0.95 | 1.07 | 0.82 | 0.98 | 0.92 | 1.05 | 0.56 | 0.97 | 0.87 | 1.08 | 0.57 | 1.01 | 0.90 | 1.12 | 0.90 |
| rs3812163 | 6 | 7725760 | T | A | 0.96 | 0.91 | 1.00 | 0.08 | 0.94 | 0.87 | 1.01 | 0.09 | 0.99 | 0.95 | 1.04 | 0.81 | 0.96 | 0.88 | 1.06 | 0.45 | 0.98 | 0.92 | 1.03 | 0.42 |
| rs1047014 | 6 | 19841493 | C | T | 1.00 | 0.94 | 1.07 | 0.94 | 0.93 | 0.87 | 1.00 | 0.04 | 1.02 | 0.96 | 1.08 | 0.56 | 1.05 | 0.88 | 1.25 | 0.58 | 0.97 | 0.90 | 1.05 | 0.45 |
| rs806794 | 6 | 26200677 | A | G | 0.99 | 0.94 | 1.04 | 0.63 | 1.04 | 0.98 | 1.09 | 0.18 | 0.94 | 0.86 | 1.03 | 0.16 | 1.02 | 0.97 | 1.08 | 0.46 | 0.93 | 0.83 | 1.05 | 0.24 |
| rs3129109 | 6 | 29084232 | C | T | 1.03 | 0.98 | 1.09 | 0.26 | 1.05 | 0.96 | 1.14 | 0.30 | 1.01 | 0.96 | 1.08 | 0.63 | 1.04 | 0.99 | 1.08 | 0.12 | 1.03 | 0.93 | 1.13 | 0.61 |
| rs114684762 | 6 | 31380529 | A | G | 1.02 | 0.98 | 1.06 | 0.38 | 1.03 | 0.95 | 1.12 | 0.50 | 1.01 | 0.92 | 1.10 | 0.85 | 1.01 | 0.95 | 1.08 | 0.73 | 1.01 | 0.94 | 1.09 | 0.72 |
| rs115961701 | 6 | 32663999 | G | C | 0.93 | 0.89 | 0.98 | 0.01 | 0.90 | 0.84 | 0.97 | 0.005 | 0.93 | 0.86 | 1.01 | 0.07 | 0.88 | 0.84 | 0.92 | <0.001 | 0.92 | 0.86 | 0.99 | 0.03 |
| rs2780226 | 6 | 34199092 | C | T | 1.00 | 0.90 | 1.11 | 0.98 | 0.99 | 0.84 | 1.16 | 0.88 | 0.97 | 0.85 | 1.12 | 0.71 | 1.00 | 0.91 | 1.10 | 0.97 | 0.98 | 0.86 | 1.12 | 0.78 |
| rs6457821 | 6 | 35402805 | C | A | 1.19 | 1.02 | 1.39 | 0.03 | 1.21 | 0.93 | 1.57 | 0.16 | 1.24 | 0.90 | 1.71 | 0.20 | 1.62 | 0.97 | 2.71 | 0.07 | 1.06 | 0.72 | 1.55 | 0.77 |
| rs9472414 | 6 | 44946506 | T | A | 0.98 | 0.95 | 1.00 | 0.04 | 0.96 | 0.92 | 1.00 | 0.05 | 1.05 | 0.95 | 1.17 | 0.36 | 1.00 | 0.91 | 1.11 | 0.93 | 0.98 | 0.92 | 1.04 | 0.48 |
| rs9360921 | 6 | 76265642 | G | T | 1.11 | 1.05 | 1.16 | <0.001 | 1.15 | 1.07 | 1.23 | <0.001 | 1.05 | 0.90 | 1.21 | 0.57 | 1.12 | 1.04 | 1.20 | 0.003 | 1.04 | 0.94 | 1.14 | 0.43 |
| rs310405 | 6 | 81800362 | A | G | 1.02 | 0.99 | 1.05 | 0.19 | 1.07 | 1.03 | 1.11 | <0.001 | 0.99 | 0.93 | 1.07 | 0.86 | 1.04 | 0.97 | 1.12 | 0.26 | 1.05 | 0.99 | 1.10 | 0.10 |
| rs7759938 | 6 | 105378954 | T | C | 1.00 | 0.97 | 1.03 | 0.94 | 1.04 | 0.99 | 1.10 | 0.11 | 1.01 | 0.94 | 1.09 | 0.80 | 1.09 | 0.97 | 1.22 | 0.15 | 0.97 | 0.89 | 1.06 | 0.53 |
| rs1046943 | 6 | 109783941 | G | A | 1.03 | 1.00 | 1.06 | 0.03 | 1.05 | 0.99 | 1.11 | 0.09 | 1.02 | 0.96 | 1.08 | 0.56 | 0.98 | 0.91 | 1.04 | 0.45 | 1.04 | 0.95 | 1.13 | 0.43 |
| rs961764 | 6 | 117522156 | C | G | 1.00 | 0.96 | 1.04 | 0.87 | 0.96 | 0.88 | 1.05 | 0.41 | 1.02 | 0.96 | 1.08 | 0.63 | 0.98 | 0.88 | 1.09 | 0.73 | 1.01 | 0.94 | 1.09 | 0.78 |
| rs1490384 | 6 | 126851160 | C | T | 1.00 | 0.97 | 1.03 | 0.97 | 0.98 | 0.93 | 1.02 | 0.34 | 1.01 | 0.96 | 1.06 | 0.72 | 0.96 | 0.87 | 1.06 | 0.42 | 1.05 | 1.00 | 1.09 | 0.04 |
| rs6569648 | 6 | 130349119 | C | T | 1.00 | 0.96 | 1.05 | 0.93 | 1.01 | 0.94 | 1.08 | 0.86 | 1.02 | 0.96 | 1.08 | 0.59 | 0.97 | 0.89 | 1.07 | 0.58 | 1.02 | 0.88 | 1.17 | 0.83 |
| rs7763064 | 6 | 142797289 | G | A | 1.01 | 0.97 | 1.06 | 0.57 | 1.03 | 0.97 | 1.10 | 0.27 | 1.05 | 0.99 | 1.11 | 0.10 | 1.10 | 1.02 | 1.19 | 0.02 | 1.00 | 0.92 | 1.09 | 0.98 |
| rs543650 | 6 | 152110943 | G | T | 1.01 | 0.97 | 1.04 | 0.73 | 0.96 | 0.91 | 1.01 | 0.14 | 1.03 | 0.97 | 1.09 | 0.35 | 0.95 | 0.90 | 1.01 | 0.09 | 1.03 | 0.98 | 1.10 | 0.25 |
| rs9456307 | 6 | 158929442 | T | A | 1.05 | 0.89 | 1.23 | 0.58 | 0.94 | 0.75 | 1.18 | 0.59 | 1.15 | 0.98 | 1.34 | 0.09 | 1.13 | 0.88 | 1.44 | 0.34 | 1.22 | 0.99 | 1.50 | 0.06 |
| rs798489 | 7 | 2801803 | C | T | 1.03 | 0.97 | 1.10 | 0.30 | 1.12 | 1.01 | 1.24 | 0.03 | 0.93 | 0.81 | 1.08 | 0.36 | 1.15 | 1.02 | 1.29 | 0.02 | 1.01 | 0.92 | 1.12 | 0.77 |
| rs4470914 | 7 | 19616522 | T | C | 1.02 | 0.95 | 1.10 | 0.57 | 1.04 | 0.94 | 1.14 | 0.46 | 1.05 | 0.94 | 1.17 | 0.39 | 1.02 | 0.90 | 1.16 | 0.76 | 0.97 | 0.86 | 1.10 | 0.63 |
| rs12534093 | 7 | 23502974 | T | A | 1.00 | 0.95 | 1.04 | 0.90 | 1.01 | 0.94 | 1.07 | 0.86 | 0.99 | 0.91 | 1.07 | 0.76 | 1.07 | 1.02 | 1.12 | 0.008 | 1.02 | 0.94 | 1.11 | 0.66 |
| rs1708299 | 7 | 28189946 | A | G | 1.00 | 0.96 | 1.05 | 0.85 | 0.98 | 0.90 | 1.07 | 0.62 | 1.02 | 0.98 | 1.06 | 0.39 | 0.96 | 0.86 | 1.07 | 0.48 | 1.01 | 0.93 | 1.09 | 0.90 |
| rs6959212 | 7 | 38128326 | C | T | 1.02 | 0.97 | 1.07 | 0.41 | 1.04 | 0.97 | 1.11 | 0.29 | 1.00 | 0.90 | 1.11 | 0.98 | 1.04 | 0.94 | 1.15 | 0.48 | 0.99 | 0.92 | 1.06 | 0.70 |
| rs42235 | 7 | 92248076 | T | C | 0.98 | 0.93 | 1.02 | 0.32 | 0.95 | 0.91 | 0.99 | 0.03 | 0.99 | 0.94 | 1.05 | 0.79 | 0.95 | 0.89 | 1.00 | 0.04 | 0.99 | 0.94 | 1.05 | 0.75 |
| rs822552 | 7 | 148650634 | G | C | 0.98 | 0.96 | 1.01 | 0.19 | 1.00 | 0.97 | 1.03 | 0.90 | 1.04 | 0.99 | 1.09 | 0.13 | 1.03 | 0.96 | 1.09 | 0.40 | 0.99 | 0.94 | 1.05 | 0.78 |
| rs77505230 | 7 | 150517022 | G | C | 0.97 | 0.93 | 1.01 | 0.19 | 0.95 | 0.88 | 1.04 | 0.29 | 1.00 | 0.95 | 1.05 | 0.94 | 0.95 | 0.87 | 1.04 | 0.25 | 0.99 | 0.94 | 1.04 | 0.60 |
| rs1013209 | 8 | 24116304 | C | T | 1.02 | 0.95 | 1.09 | 0.62 | 0.97 | 0.87 | 1.08 | 0.56 | 1.13 | 1.05 | 1.21 | 0.001 | 1.05 | 0.99 | 1.11 | 0.10 | 1.05 | 0.93 | 1.17 | 0.43 |
| rs7460090 | 8 | 57194163 | T | C | 1.04 | 0.99 | 1.09 | 0.12 | 1.03 | 0.95 | 1.12 | 0.51 | 1.14 | 0.96 | 1.35 | 0.13 | 1.01 | 0.89 | 1.15 | 0.83 | 1.08 | 0.99 | 1.18 | 0.10 |
| rs6473015 | 8 | 78178485 | C | A | 1.01 | 0.96 | 1.06 | 0.82 | 1.06 | 0.96 | 1.16 | 0.27 | 0.93 | 0.77 | 1.11 | 0.42 | 0.98 | 0.89 | 1.08 | 0.65 | 1.02 | 0.92 | 1.14 | 0.68 |
| rs6470764 | 8 | 130725665 | C | T | 1.04 | 0.98 | 1.09 | 0.18 | 1.04 | 0.96 | 1.14 | 0.33 | 1.06 | 0.97 | 1.15 | 0.23 | 1.08 | 0.96 | 1.22 | 0.21 | 1.05 | 1.01 | 1.09 | 0.02 |
| rs12680655 | 8 | 135637337 | C | G | 1.00 | 0.96 | 1.04 | 0.90 | 1.00 | 0.92 | 1.10 | 0.95 | 1.06 | 1.01 | 1.12 | 0.01 | 1.01 | 0.95 | 1.08 | 0.71 | 1.02 | 0.98 | 1.06 | 0.44 |
| rs7864648 | 9 | 16368732 | T | G | 1.03 | 1.00 | 1.07 | 0.08 | 1.06 | 1.00 | 1.13 | 0.05 | 1.05 | 0.96 | 1.15 | 0.31 | 1.07 | 0.99 | 1.15 | 0.10 | 1.04 | 0.99 | 1.09 | 0.10 |
| rs11144688 | 9 | 78542286 | G | A | 0.98 | 0.92 | 1.05 | 0.57 | 0.91 | 0.83 | 1.00 | 0.06 | 1.00 | 0.93 | 1.08 | 0.97 | 1.12 | 0.99 | 1.26 | 0.06 | 0.91 | 0.83 | 1.01 | 0.07 |
| rs7853377 | 9 | 86552205 | G | A | 1.04 | 0.99 | 1.08 | 0.09 | 1.02 | 0.90 | 1.16 | 0.72 | 1.09 | 1.01 | 1.17 | 0.03 | 1.09 | 0.97 | 1.23 | 0.15 | 1.01 | 0.96 | 1.07 | 0.74 |
| rs8181166 | 9 | 89116628 | C | G | 0.98 | 0.92 | 1.04 | 0.47 | 0.99 | 0.94 | 1.05 | 0.85 | 0.97 | 0.89 | 1.05 | 0.41 | 1.00 | 0.88 | 1.13 | 0.96 | 0.97 | 0.91 | 1.03 | 0.34 |
| rs2778031 | 9 | 90835726 | T | C | 1.07 | 1.02 | 1.13 | 0.01 | 1.09 | 1.00 | 1.19 | 0.04 | 1.05 | 0.93 | 1.18 | 0.41 | 1.11 | 0.98 | 1.25 | 0.10 | 1.02 | 0.94 | 1.12 | 0.58 |
| rs9969804 | 9 | 95429120 | A | C | 1.00 | 0.96 | 1.03 | 0.85 | 0.97 | 0.92 | 1.02 | 0.21 | 1.00 | 0.93 | 1.08 | 0.93 | 1.01 | 0.92 | 1.11 | 0.81 | 1.02 | 0.95 | 1.09 | 0.55 |
| rs1257763 | 9 | 96893945 | A | G | 0.99 | 0.87 | 1.12 | 0.88 | 1.04 | 0.86 | 1.26 | 0.69 | 0.87 | 0.79 | 0.95 | 0.003 | 0.94 | 0.70 | 1.28 | 0.70 | 1.03 | 0.87 | 1.23 | 0.72 |
| rs473902 | 9 | 98256235 | T | G | 0.97 | 0.89 | 1.07 | 0.56 | 0.94 | 0.84 | 1.05 | 0.28 | 1.00 | 0.83 | 1.20 | 0.98 | 0.88 | 0.78 | 1.01 | 0.06 | 0.97 | 0.81 | 1.15 | 0.70 |
| rs7027110 | 9 | 109599046 | A | G | 0.98 | 0.94 | 1.02 | 0.25 | 0.97 | 0.89 | 1.06 | 0.49 | 0.98 | 0.92 | 1.05 | 0.55 | 0.99 | 0.92 | 1.07 | 0.81 | 0.98 | 0.93 | 1.03 | 0.37 |
| rs1468758 | 9 | 113807082 | C | T | 1.05 | 0.99 | 1.11 | 0.08 | 1.02 | 0.96 | 1.08 | 0.56 | 1.07 | 0.98 | 1.17 | 0.14 | 1.12 | 1.02 | 1.23 | 0.02 | 1.08 | 0.98 | 1.18 | 0.11 |
| rs751543 | 9 | 119122342 | T | C | 0.98 | 0.95 | 1.02 | 0.35 | 0.97 | 0.92 | 1.02 | 0.21 | 1.02 | 0.95 | 1.09 | 0.64 | 0.95 | 0.91 | 1.00 | 0.07 | 1.04 | 0.97 | 1.11 | 0.33 |
| rs7466269 | 9 | 133464084 | A | G | 0.97 | 0.91 | 1.03 | 0.33 | 0.96 | 0.90 | 1.04 | 0.31 | 0.95 | 0.87 | 1.05 | 0.30 | 0.95 | 0.83 | 1.09 | 0.46 | 0.97 | 0.88 | 1.06 | 0.47 |
| rs7849585 | 9 | 139111870 | T | G | 0.99 | 0.94 | 1.04 | 0.71 | 0.99 | 0.89 | 1.09 | 0.80 | 1.02 | 0.97 | 1.08 | 0.42 | 1.04 | 0.94 | 1.15 | 0.45 | 0.98 | 0.91 | 1.05 | 0.49 |
| rs7909670 | 10 | 12918764 | C | T | 0.96 | 0.91 | 1.02 | 0.19 | 0.96 | 0.88 | 1.05 | 0.39 | 0.97 | 0.92 | 1.02 | 0.24 | 0.96 | 0.85 | 1.08 | 0.46 | 0.94 | 0.86 | 1.02 | 0.13 |
| rs2145998 | 10 | 81121696 | T | A | 0.99 | 0.95 | 1.03 | 0.50 | 0.99 | 0.93 | 1.04 | 0.65 | 0.98 | 0.94 | 1.03 | 0.48 | 0.99 | 0.89 | 1.11 | 0.91 | 0.99 | 0.95 | 1.03 | 0.66 |
| rs11599750 | 10 | 101805442 | C | T | 0.96 | 0.91 | 1.01 | 0.10 | 0.99 | 0.92 | 1.06 | 0.76 | 0.94 | 0.90 | 0.97 | <0.001 | 1.04 | 0.94 | 1.16 | 0.46 | 0.95 | 0.90 | 1.00 | 0.05 |
| rs2237886 | 11 | 2810731 | T | C | 0.97 | 0.91 | 1.05 | 0.48 | 0.93 | 0.85 | 1.01 | 0.09 | 0.97 | 0.88 | 1.06 | 0.49 | 0.98 | 0.89 | 1.08 | 0.73 | 0.99 | 0.88 | 1.10 | 0.80 |
| rs7926971 | 11 | 12698040 | G | A | 0.99 | 0.94 | 1.04 | 0.59 | 1.01 | 0.95 | 1.07 | 0.82 | 0.94 | 0.88 | 1.00 | 0.04 | 0.96 | 0.90 | 1.02 | 0.21 | 1.01 | 0.96 | 1.07 | 0.67 |
| rs1330 | 11 | 17316029 | T | C | 0.98 | 0.96 | 1.01 | 0.18 | 0.99 | 0.93 | 1.05 | 0.75 | 0.99 | 0.91 | 1.07 | 0.80 | 0.99 | 0.93 | 1.07 | 0.89 | 0.97 | 0.91 | 1.02 | 0.24 |
| rs79890777 | 11 | 48098280 | G | A | 1.01 | 0.98 | 1.04 | 0.48 | 1.01 | 0.98 | 1.04 | 0.60 | 1.06 | 0.99 | 1.14 | 0.11 | 1.05 | 0.99 | 1.12 | 0.09 | 1.00 | 0.94 | 1.07 | 0.94 |
| rs1814175 | 11 | 49559172 | T | C | 1.00 | 0.95 | 1.05 | 0.90 | 0.96 | 0.91 | 1.02 | 0.17 | 1.03 | 0.98 | 1.08 | 0.18 | 0.97 | 0.87 | 1.09 | 0.61 | 1.02 | 0.92 | 1.14 | 0.64 |
| rs3782089 | 11 | 65336819 | C | T | 1.00 | 0.93 | 1.06 | 0.89 | 0.98 | 0.91 | 1.05 | 0.52 | 1.08 | 0.90 | 1.30 | 0.42 | 0.94 | 0.81 | 1.09 | 0.40 | 0.97 | 0.84 | 1.12 | 0.65 |
| rs7112925 | 11 | 66826160 | C | T | 1.00 | 0.93 | 1.08 | 0.98 | 1.01 | 0.94 | 1.09 | 0.76 | 0.94 | 0.87 | 1.00 | 0.07 | 0.97 | 0.87 | 1.09 | 0.63 | 0.97 | 0.87 | 1.08 | 0.58 |
| rs634552 | 11 | 75282052 | T | G | 1.09 | 1.03 | 1.16 | 0.006 | 1.08 | 1.00 | 1.17 | 0.04 | 1.11 | 1.03 | 1.20 | 0.009 | 1.12 | 1.00 | 1.25 | 0.05 | 1.08 | 1.02 | 1.15 | 0.008 |
| rs494459 | 11 | 118574675 | T | C | 1.00 | 0.96 | 1.05 | 0.85 | 0.99 | 0.91 | 1.07 | 0.73 | 1.04 | 1.01 | 1.08 | 0.01 | 1.01 | 0.96 | 1.07 | 0.66 | 1.01 | 0.95 | 1.08 | 0.76 |
| rs654723 | 11 | 128586155 | A | C | 0.99 | 0.97 | 1.02 | 0.67 | 1.00 | 0.95 | 1.05 | 0.94 | 1.04 | 1.01 | 1.08 | 0.02 | 0.91 | 0.86 | 0.97 | 0.002 | 1.08 | 1.04 | 1.13 | <0.001 |
| rs2856321 | 12 | 11855773 | G | A | 0.99 | 0.94 | 1.04 | 0.69 | 1.00 | 0.92 | 1.09 | 0.96 | 0.99 | 0.95 | 1.03 | 0.50 | 0.96 | 0.86 | 1.08 | 0.53 | 1.00 | 0.95 | 1.06 | 0.93 |
| rs10770705 | 12 | 20857467 | A | C | 1.02 | 0.97 | 1.07 | 0.52 | 0.93 | 0.87 | 0.99 | 0.03 | 1.05 | 0.98 | 1.13 | 0.13 | 1.02 | 0.92 | 1.12 | 0.74 | 1.00 | 0.91 | 1.11 | 0.95 |
| rs2638953 | 12 | 28534415 | C | G | 1.02 | 0.99 | 1.06 | 0.24 | 0.99 | 0.92 | 1.07 | 0.80 | 1.08 | 1.04 | 1.12 | <0.001 | 1.03 | 0.94 | 1.14 | 0.49 | 0.99 | 0.92 | 1.07 | 0.81 |
| rs2066807 | 12 | 56740682 | G | C | 0.98 | 0.91 | 1.05 | 0.54 | 0.90 | 0.78 | 1.04 | 0.15 | 0.97 | 0.84 | 1.12 | 0.65 | 0.92 | 0.74 | 1.15 | 0.47 | 0.95 | 0.86 | 1.05 | 0.32 |
| rs1351394 | 12 | 66351826 | T | C | 0.99 | 0.95 | 1.03 | 0.58 | 0.98 | 0.90 | 1.05 | 0.53 | 1.04 | 0.96 | 1.12 | 0.32 | 0.98 | 0.88 | 1.08 | 0.63 | 0.99 | 0.95 | 1.02 | 0.51 |
| rs10748128 | 12 | 69827658 | T | G | 1.00 | 0.96 | 1.05 | 0.85 | 0.99 | 0.93 | 1.05 | 0.81 | 1.06 | 1.00 | 1.13 | 0.06 | 1.06 | 0.99 | 1.12 | 0.08 | 0.96 | 0.89 | 1.05 | 0.37 |
| rs11107116 | 12 | 93978504 | T | G | 1.03 | 0.99 | 1.07 | 0.15 | 1.00 | 0.93 | 1.08 | 0.97 | 1.06 | 1.00 | 1.13 | 0.05 | 1.00 | 0.94 | 1.05 | 0.89 | 1.05 | 0.98 | 1.13 | 0.15 |
| rs7971536 | 12 | 102373788 | T | A | 0.98 | 0.94 | 1.03 | 0.42 | 1.01 | 0.95 | 1.07 | 0.83 | 0.97 | 0.91 | 1.02 | 0.23 | 0.96 | 0.91 | 1.01 | 0.15 | 0.98 | 0.92 | 1.04 | 0.49 |
| rs11830103 | 12 | 123823546 | G | A | 1.00 | 0.97 | 1.03 | 0.78 | 0.99 | 0.92 | 1.06 | 0.72 | 1.03 | 0.98 | 1.08 | 0.25 | 1.05 | 0.95 | 1.16 | 0.38 | 0.94 | 0.88 | 1.00 | 0.04 |
| rs7332115 | 13 | 33147548 | G | T | 1.01 | 0.97 | 1.05 | 0.73 | 0.97 | 0.92 | 1.03 | 0.34 | 0.97 | 0.90 | 1.04 | 0.34 | 0.98 | 0.92 | 1.04 | 0.48 | 1.02 | 0.99 | 1.06 | 0.19 |
| rs3118905 | 13 | 51105334 | G | A | 1.01 | 0.96 | 1.06 | 0.76 | 1.00 | 0.93 | 1.07 | 0.98 | 1.05 | 1.00 | 1.10 | 0.05 | 0.96 | 0.92 | 1.01 | 0.14 | 1.02 | 0.96 | 1.08 | 0.57 |
| rs7319045 | 13 | 92024574 | A | G | 0.99 | 0.92 | 1.06 | 0.78 | 1.03 | 0.94 | 1.12 | 0.56 | 0.97 | 0.89 | 1.05 | 0.41 | 0.94 | 0.83 | 1.06 | 0.32 | 1.02 | 0.94 | 1.12 | 0.60 |
| rs1950500 | 14 | 24830850 | T | C | 1.00 | 0.97 | 1.03 | 0.75 | 1.00 | 0.91 | 1.08 | 0.91 | 0.98 | 0.94 | 1.03 | 0.44 | 0.94 | 0.88 | 1.00 | 0.04 | 1.10 | 1.05 | 1.16 | <0.001 |
| rs2093210 | 14 | 60957279 | C | T | 1.01 | 0.97 | 1.05 | 0.56 | 1.07 | 1.00 | 1.15 | 0.04 | 0.99 | 0.94 | 1.05 | 0.85 | 1.06 | 0.97 | 1.15 | 0.18 | 0.95 | 0.86 | 1.04 | 0.24 |
| rs1570106 | 14 | 68813115 | C | T | 0.98 | 0.93 | 1.04 | 0.53 | 1.01 | 0.93 | 1.09 | 0.90 | 0.96 | 0.89 | 1.03 | 0.29 | 0.99 | 0.91 | 1.08 | 0.80 | 1.00 | 0.94 | 1.07 | 0.91 |
| rs862034 | 14 | 74990746 | G | A | 1.01 | 0.96 | 1.06 | 0.67 | 0.97 | 0.91 | 1.04 | 0.39 | 1.08 | 1.00 | 1.16 | 0.05 | 0.98 | 0.90 | 1.06 | 0.59 | 1.07 | 1.01 | 1.14 | 0.03 |
| rs7155279 | 14 | 92485881 | G | T | 0.97 | 0.95 | 0.99 | 0.01 | 0.95 | 0.90 | 0.99 | 0.02 | 1.00 | 0.96 | 1.05 | 0.87 | 0.96 | 0.91 | 1.01 | 0.15 | 0.97 | 0.92 | 1.02 | 0.21 |
| rs16964211 | 15 | 51530495 | G | A | 0.98 | 0.86 | 1.13 | 0.80 | 0.89 | 0.70 | 1.13 | 0.33 | 1.04 | 0.87 | 1.25 | 0.65 | 0.99 | 0.83 | 1.17 | 0.89 | 1.08 | 0.89 | 1.32 | 0.43 |
| rs7178424 | 15 | 62380259 | C | T | 1.00 | 0.96 | 1.04 | 0.96 | 0.95 | 0.89 | 1.02 | 0.16 | 1.08 | 1.04 | 1.11 | <0.001 | 0.96 | 0.87 | 1.05 | 0.34 | 0.98 | 0.92 | 1.05 | 0.62 |
| rs10152591 | 15 | 70048157 | A | C | 1.01 | 0.91 | 1.12 | 0.85 | 1.02 | 0.90 | 1.17 | 0.72 | 0.94 | 0.83 | 1.07 | 0.35 | 0.98 | 0.79 | 1.22 | 0.87 | 1.02 | 0.95 | 1.10 | 0.53 |
| rs12902421 | 15 | 72161403 | C | T | 1.07 | 0.84 | 1.35 | 0.60 | 1.17 | 0.82 | 1.66 | 0.38 | 0.72 | 0.34 | 1.52 | 0.39 | 0.93 | 0.56 | 1.55 | 0.79 | 1.06 | 0.75 | 1.50 | 0.75 |
| rs5742915 | 15 | 74336633 | C | T | 1.01 | 0.97 | 1.05 | 0.57 | 1.01 | 0.93 | 1.11 | 0.76 | 1.01 | 0.95 | 1.06 | 0.80 | 1.03 | 0.97 | 1.10 | 0.28 | 0.94 | 0.88 | 1.01 | 0.08 |
| rs11259936 | 15 | 84580582 | C | A | 1.02 | 0.96 | 1.07 | 0.55 | 0.97 | 0.88 | 1.08 | 0.59 | 1.08 | 1.01 | 1.17 | 0.03 | 1.02 | 0.92 | 1.14 | 0.69 | 0.99 | 0.92 | 1.06 | 0.72 |
| rs16942341 | 15 | 89388905 | C | T | 1.05 | 0.92 | 1.21 | 0.46 | 1.08 | 0.88 | 1.33 | 0.46 | 1.01 | 0.84 | 1.21 | 0.94 | 0.85 | 0.71 | 1.01 | 0.07 | 1.13 | 0.94 | 1.37 | 0.20 |
| rs2871865 | 15 | 99194896 | C | G | 0.94 | 0.90 | 0.99 | 0.01 | 0.89 | 0.80 | 0.99 | 0.04 | 0.95 | 0.86 | 1.05 | 0.33 | 0.92 | 0.80 | 1.07 | 0.29 | 0.94 | 0.83 | 1.06 | 0.28 |
| rs4965598 | 15 | 100759614 | C | T | 0.99 | 0.94 | 1.06 | 0.86 | 1.00 | 0.92 | 1.08 | 0.96 | 1.06 | 0.96 | 1.16 | 0.26 | 0.89 | 0.82 | 0.98 | 0.01 | 1.07 | 1.00 | 1.15 | 0.06 |
| rs11648796 | 16 | 792190 | G | A | 1.02 | 0.98 | 1.05 | 0.37 | 1.02 | 0.98 | 1.06 | 0.28 | 1.06 | 0.98 | 1.15 | 0.17 | 1.07 | 1.02 | 1.12 | 0.002 | 1.05 | 0.98 | 1.13 | 0.19 |
| rs26868 | 16 | 2249376 | A | T | 1.02 | 0.96 | 1.08 | 0.54 | 1.03 | 0.94 | 1.13 | 0.58 | 1.01 | 0.95 | 1.07 | 0.84 | 1.01 | 0.93 | 1.09 | 0.86 | 1.03 | 0.95 | 1.11 | 0.46 |
| rs1659127 | 16 | 14388305 | A | G | 1.00 | 0.96 | 1.05 | 0.83 | 1.02 | 0.98 | 1.07 | 0.29 | 0.96 | 0.86 | 1.08 | 0.53 | 1.04 | 0.90 | 1.20 | 0.60 | 0.98 | 0.92 | 1.03 | 0.36 |
| rs8052560 | 16 | 88777242 | A | C | 1.02 | 0.98 | 1.06 | 0.35 | 1.02 | 0.93 | 1.11 | 0.72 | 0.98 | 0.89 | 1.08 | 0.72 | 1.06 | 0.98 | 1.15 | 0.17 | 1.01 | 0.96 | 1.07 | 0.66 |
| rs4640244 | 17 | 21284223 | A | G | 0.98 | 0.93 | 1.03 | 0.42 | 0.92 | 0.84 | 1.02 | 0.10 | 1.05 | 0.97 | 1.14 | 0.21 | 0.91 | 0.85 | 0.98 | 0.01 | 1.01 | 0.94 | 1.08 | 0.87 |
| rs3110496 | 17 | 27917771 | G | A | 1.00 | 0.95 | 1.06 | 0.89 | 1.00 | 0.95 | 1.05 | 0.88 | 1.05 | 1.00 | 1.10 | 0.07 | 1.01 | 0.95 | 1.07 | 0.76 | 1.02 | 0.96 | 1.09 | 0.50 |
| rs3764419 | 17 | 29164023 | C | A | 0.98 | 0.94 | 1.03 | 0.52 | 1.01 | 0.92 | 1.11 | 0.84 | 0.96 | 0.89 | 1.03 | 0.26 | 1.02 | 0.94 | 1.11 | 0.57 | 0.97 | 0.89 | 1.04 | 0.36 |
| rs17780086 | 17 | 30343282 | A | G | 1.05 | 0.98 | 1.12 | 0.17 | 1.06 | 0.95 | 1.18 | 0.32 | 1.11 | 0.95 | 1.29 | 0.20 | 1.02 | 0.89 | 1.17 | 0.78 | 1.06 | 0.98 | 1.15 | 0.16 |
| rs1043515 | 17 | 36922196 | G | A | 1.01 | 0.97 | 1.05 | 0.52 | 0.99 | 0.94 | 1.05 | 0.71 | 1.03 | 0.98 | 1.09 | 0.24 | 0.99 | 0.92 | 1.06 | 0.67 | 1.04 | 1.00 | 1.08 | 0.07 |
| rs4986172 | 17 | 43216281 | C | T | 0.99 | 0.96 | 1.03 | 0.73 | 1.00 | 0.94 | 1.06 | 0.99 | 1.03 | 0.98 | 1.08 | 0.22 | 0.99 | 0.92 | 1.07 | 0.83 | 0.99 | 0.93 | 1.05 | 0.70 |
| rs2072153 | 17 | 47390014 | C | G | 0.97 | 0.92 | 1.03 | 0.33 | 0.93 | 0.84 | 1.02 | 0.14 | 1.02 | 0.96 | 1.08 | 0.54 | 0.87 | 0.78 | 0.98 | 0.02 | 0.94 | 0.88 | 1.01 | 0.09 |
| rs4605213 | 17 | 49244747 | C | G | 1.03 | 1.01 | 1.05 | 0.008 | 0.98 | 0.94 | 1.02 | 0.25 | 1.07 | 1.01 | 1.14 | 0.02 | 1.01 | 0.94 | 1.07 | 0.87 | 1.06 | 1.01 | 1.12 | 0.03 |
| rs227724 | 17 | 54778817 | T | A | 0.98 | 0.93 | 1.03 | 0.37 | 0.96 | 0.89 | 1.03 | 0.23 | 0.99 | 0.90 | 1.08 | 0.77 | 0.98 | 0.88 | 1.09 | 0.66 | 0.96 | 0.90 | 1.04 | 0.33 |
| rs2079795 | 17 | 59496649 | T | C | 0.96 | 0.90 | 1.02 | 0.17 | 0.93 | 0.84 | 1.04 | 0.23 | 0.98 | 0.92 | 1.05 | 0.61 | 0.98 | 0.86 | 1.11 | 0.71 | 0.93 | 0.84 | 1.03 | 0.16 |
| rs2665838 | 17 | 61966465 | G | C | 1.03 | 0.99 | 1.08 | 0.16 | 1.06 | 0.97 | 1.15 | 0.20 | 1.01 | 0.95 | 1.08 | 0.73 | 1.01 | 0.94 | 1.08 | 0.84 | 1.08 | 1.02 | 1.14 | 0.01 |
| rs11867479 | 17 | 68090207 | T | C | 0.97 | 0.94 | 1.01 | 0.16 | 1.01 | 0.96 | 1.06 | 0.75 | 0.97 | 0.93 | 1.00 | 0.06 | 1.01 | 0.96 | 1.07 | 0.65 | 0.96 | 0.90 | 1.02 | 0.21 |
| rs4800452 | 18 | 20727611 | T | C | 1.04 | 1.01 | 1.08 | 0.007 | 1.04 | 0.99 | 1.09 | 0.11 | 1.07 | 1.02 | 1.13 | 0.003 | 1.07 | 0.99 | 1.16 | 0.10 | 1.02 | 0.92 | 1.12 | 0.74 |
| rs9967417 | 18 | 46959500 | G | C | 0.98 | 0.94 | 1.03 | 0.47 | 0.98 | 0.92 | 1.04 | 0.43 | 0.99 | 0.94 | 1.04 | 0.70 | 0.98 | 0.87 | 1.11 | 0.76 | 1.02 | 0.95 | 1.09 | 0.68 |
| rs17782313 | 18 | 57851097 | C | T | 0.99 | 0.96 | 1.02 | 0.53 | 0.98 | 0.93 | 1.03 | 0.42 | 1.05 | 0.99 | 1.11 | 0.10 | 1.05 | 0.96 | 1.14 | 0.32 | 0.98 | 0.92 | 1.04 | 0.52 |
| rs12982744 | 19 | 2177193 | G | C | 1.02 | 0.98 | 1.07 | 0.31 | 1.01 | 0.95 | 1.06 | 0.85 | 1.02 | 0.94 | 1.12 | 0.61 | 1.01 | 0.94 | 1.09 | 0.77 | 1.00 | 0.90 | 1.10 | 0.97 |
| rs7507204 | 19 | 3428834 | C | G | 0.97 | 0.91 | 1.02 | 0.24 | 0.97 | 0.88 | 1.07 | 0.57 | 0.93 | 0.87 | 1.00 | 0.04 | 0.99 | 0.89 | 1.11 | 0.88 | 0.99 | 0.94 | 1.05 | 0.81 |
| rs891088 | 19 | 7184762 | G | A | 1.01 | 0.97 | 1.06 | 0.57 | 1.03 | 0.95 | 1.10 | 0.50 | 1.00 | 0.93 | 1.07 | 0.97 | 0.95 | 0.88 | 1.02 | 0.14 | 1.05 | 0.98 | 1.13 | 0.18 |
| rs4072910 | 19 | 8644031 | G | C | 0.98 | 0.94 | 1.02 | 0.34 | 1.06 | 0.98 | 1.14 | 0.13 | 0.96 | 0.90 | 1.02 | 0.20 | 1.11 | 1.03 | 1.18 | 0.004 | 0.96 | 0.86 | 1.07 | 0.42 |
| rs2279008 | 19 | 17283303 | T | C | 0.99 | 0.95 | 1.03 | 0.48 | 1.02 | 0.95 | 1.10 | 0.53 | 0.95 | 0.89 | 1.01 | 0.09 | 1.03 | 0.93 | 1.15 | 0.53 | 0.96 | 0.91 | 1.02 | 0.23 |
| rs17318596 | 19 | 41937095 | A | G | 1.00 | 0.96 | 1.03 | 0.82 | 1.02 | 0.97 | 1.08 | 0.44 | 0.97 | 0.91 | 1.03 | 0.33 | 1.01 | 0.95 | 1.07 | 0.81 | 1.02 | 0.96 | 1.08 | 0.49 |
| rs1741344 | 20 | 4101800 | C | T | 1.02 | 0.97 | 1.08 | 0.48 | 1.12 | 1.03 | 1.22 | 0.006 | 0.94 | 0.90 | 0.98 | 0.005 | 1.05 | 0.98 | 1.12 | 0.18 | 1.02 | 0.95 | 1.10 | 0.64 |
| rs2145272 | 20 | 6626218 | G | A | 1.00 | 0.94 | 1.07 | 0.91 | 1.03 | 0.95 | 1.11 | 0.49 | 1.05 | 0.96 | 1.14 | 0.27 | 1.08 | 1.02 | 1.16 | 0.02 | 1.05 | 0.95 | 1.15 | 0.34 |
| rs7274811 | 20 | 32333181 | G | T | 1.03 | 0.97 | 1.09 | 0.37 | 1.02 | 0.95 | 1.09 | 0.65 | 1.07 | 0.99 | 1.15 | 0.09 | 1.02 | 0.92 | 1.13 | 0.69 | 1.03 | 0.93 | 1.13 | 0.56 |
| rs143384 | 20 | 34025756 | G | A | 1.00 | 0.95 | 1.05 | 0.94 | 1.01 | 0.94 | 1.08 | 0.78 | 1.00 | 0.95 | 1.06 | 0.87 | 1.09 | 1.01 | 1.18 | 0.02 | 0.99 | 0.92 | 1.05 | 0.67 |
| rs237743 | 20 | 47903019 | A | G | 0.96 | 0.93 | 0.99 | 0.02 | 0.92 | 0.85 | 1.00 | 0.04 | 1.01 | 0.92 | 1.12 | 0.79 | 0.95 | 0.87 | 1.05 | 0.32 | 0.95 | 0.88 | 1.03 | 0.22 |
| rs2834442 | 21 | 35690786 | A | T | 0.99 | 0.95 | 1.04 | 0.79 | 0.96 | 0.88 | 1.05 | 0.38 | 1.01 | 0.94 | 1.07 | 0.87 | 0.94 | 0.85 | 1.04 | 0.21 | 1.01 | 0.94 | 1.08 | 0.83 |
| rs4821083 | 22 | 33056341 | G | A | 1.00 | 0.93 | 1.07 | 1.00 | 1.01 | 0.94 | 1.10 | 0.71 | 1.02 | 0.92 | 1.12 | 0.77 | 1.06 | 0.98 | 1.15 | 0.12 | 0.97 | 0.89 | 1.06 | 0.50 |

Notes: *Hazard ratios are adjusted for the first 8 principal components of population stratification.*

**Supplementary Table 7: Association of 32 individual BMI SNPs with all-cause mortality after prostate cancer diagnosis. Hazard ratios indicate the effect of one extra height increasing allele.**

|  |  |  | Allele | | Any prostate cancer (N=15,491) | | | | Localised prostate cancer (N=9,100) | | | | Advanced prostate cancer (N=3,685) | | | | Low grade prostate cancer (N=6,026) | | | | High grade prostate cancer (N=6,301) | | | |
| --- | --- | --- | --- | --- | --- | --- | --- | --- | --- | --- | --- | --- | --- | --- | --- | --- | --- | --- | --- | --- | --- | --- | --- | --- |
|  |  |  | BMI | Other | Hazard | Confidence interval | | p- | Hazard | Confidence interval | | p- | Hazard | Confidence interval | | p- | Hazard | Confidence interval | | p- | Hazard | Confidence interval | | p- |
| RS id | Chr. | Position | increasing |  | ratio | Lower | Upper | value | ratio | Lower | Upper | value | ratio | Lower | Upper | value | ratio | Lower | Upper | value | ratio | Lower | Upper | value |
| rs2815752 | 1 | 72812440 | A | G | 1.00 | 0.93 | 1.07 | 0.96 | 1.03 | 0.92 | 1.15 | 0.63 | 1.01 | 0.95 | 1.07 | 0.75 | 1.05 | 0.91 | 1.22 | 0.50 | 1.00 | 0.92 | 1.07 | 0.90 |
| rs1514175 | 1 | 74991644 | A | G | 1.03 | 0.98 | 1.08 | 0.28 | 1.03 | 0.96 | 1.10 | 0.47 | 1.06 | 1.02 | 1.10 | 0.001 | 1.01 | 0.92 | 1.12 | 0.80 | 1.04 | 0.99 | 1.09 | 0.15 |
| rs1555543 | 1 | 96944797 | C | A | 1.03 | 1.01 | 1.06 | 0.02 | 1.08 | 1.04 | 1.13 | <0.001 | 0.99 | 0.94 | 1.04 | 0.64 | 1.12 | 1.07 | 1.17 | <0.001 | 0.97 | 0.91 | 1.04 | 0.39 |
| rs543874 | 1 | 177889480 | G | A | 0.99 | 0.95 | 1.03 | 0.65 | 1.01 | 0.93 | 1.09 | 0.88 | 0.96 | 0.89 | 1.04 | 0.34 | 0.95 | 0.87 | 1.03 | 0.21 | 1.02 | 0.95 | 1.11 | 0.57 |
| rs2867125 | 2 | 622827 | C | T | 1.04 | 0.97 | 1.12 | 0.22 | 0.99 | 0.90 | 1.10 | 0.89 | 1.10 | 1.03 | 1.18 | 0.008 | 1.14 | 1.04 | 1.25 | 0.006 | 0.99 | 0.89 | 1.11 | 0.87 |
| rs713586 | 2 | 25158008 | C | T | 1.02 | 0.98 | 1.06 | 0.26 | 1.06 | 0.96 | 1.17 | 0.25 | 1.01 | 0.95 | 1.07 | 0.78 | 1.04 | 0.92 | 1.18 | 0.52 | 1.01 | 0.97 | 1.06 | 0.60 |
| rs887912 | 2 | 59302877 | T | C | 1.01 | 0.96 | 1.08 | 0.63 | 1.03 | 0.95 | 1.13 | 0.47 | 0.99 | 0.94 | 1.05 | 0.82 | 1.02 | 0.93 | 1.12 | 0.66 | 1.00 | 0.89 | 1.14 | 0.96 |
| rs2890652 | 2 | 142959931 | C | T | 1.02 | 0.95 | 1.09 | 0.60 | 1.00 | 0.92 | 1.09 | 0.97 | 1.02 | 0.96 | 1.09 | 0.52 | 1.02 | 0.89 | 1.17 | 0.77 | 1.01 | 0.96 | 1.06 | 0.80 |
| rs13078807 | 3 | 85884150 | G | A | 0.97 | 0.92 | 1.02 | 0.20 | 0.92 | 0.86 | 0.98 | 0.006 | 0.96 | 0.90 | 1.03 | 0.25 | 0.91 | 0.81 | 1.03 | 0.13 | 1.01 | 0.98 | 1.04 | 0.52 |
| rs9816226 | 3 | 185834499 | T | A | 1.01 | 0.96 | 1.06 | 0.75 | 1.06 | 0.96 | 1.16 | 0.26 | 0.98 | 0.90 | 1.08 | 0.71 | 1.05 | 0.92 | 1.19 | 0.50 | 0.98 | 0.93 | 1.03 | 0.43 |
| rs10938397 | 4 | 45182527 | G | A | 0.98 | 0.93 | 1.03 | 0.49 | 0.98 | 0.91 | 1.06 | 0.59 | 0.95 | 0.88 | 1.03 | 0.22 | 1.05 | 0.91 | 1.22 | 0.50 | 0.95 | 0.89 | 1.02 | 0.18 |
| rs13107325 | 4 | 103188709 | T | C | 1.04 | 0.91 | 1.19 | 0.58 | 1.03 | 0.82 | 1.28 | 0.80 | 1.05 | 0.95 | 1.15 | 0.34 | 0.93 | 0.80 | 1.09 | 0.37 | 1.11 | 0.96 | 1.27 | 0.16 |
| rs2112347 | 5 | 75015242 | T | G | 1.03 | 0.97 | 1.08 | 0.39 | 1.04 | 0.93 | 1.16 | 0.49 | 1.01 | 0.95 | 1.08 | 0.70 | 1.06 | 0.95 | 1.19 | 0.28 | 1.01 | 0.96 | 1.07 | 0.69 |
| rs4836133 | 5 | 124332103 | A | C | 1.02 | 0.97 | 1.07 | 0.46 | 0.99 | 0.91 | 1.09 | 0.88 | 0.99 | 0.87 | 1.13 | 0.90 | 1.04 | 0.94 | 1.15 | 0.42 | 0.98 | 0.90 | 1.07 | 0.68 |
| rs206936 | 6 | 34302869 | G | A | 1.00 | 0.93 | 1.06 | 0.90 | 0.99 | 0.90 | 1.09 | 0.84 | 0.98 | 0.90 | 1.06 | 0.60 | 1.03 | 0.94 | 1.13 | 0.55 | 0.94 | 0.84 | 1.06 | 0.30 |
| rs987237 | 6 | 50803050 | G | A | 1.00 | 0.94 | 1.07 | 0.90 | 1.01 | 0.92 | 1.10 | 0.88 | 1.03 | 0.96 | 1.11 | 0.36 | 1.03 | 0.98 | 1.08 | 0.31 | 0.94 | 0.84 | 1.05 | 0.25 |
| rs10968576 | 9 | 28414339 | G | A | 1.04 | 0.99 | 1.09 | 0.10 | 1.07 | 0.98 | 1.18 | 0.12 | 1.03 | 0.97 | 1.09 | 0.30 | 1.04 | 0.98 | 1.10 | 0.19 | 0.99 | 0.96 | 1.02 | 0.52 |
| rs4929949 | 11 | 8604593 | C | T | 1.02 | 0.97 | 1.07 | 0.46 | 1.05 | 0.97 | 1.13 | 0.20 | 1.01 | 0.96 | 1.07 | 0.73 | 1.09 | 1.00 | 1.18 | 0.05 | 0.99 | 0.93 | 1.06 | 0.79 |
| rs10767664 | 11 | 27725986 | A | T | 0.97 | 0.92 | 1.02 | 0.28 | 0.97 | 0.89 | 1.05 | 0.40 | 0.93 | 0.87 | 1.00 | 0.04 | 0.98 | 0.84 | 1.15 | 0.84 | 0.95 | 0.90 | 1.00 | 0.06 |
| rs3817334 | 11 | 47650993 | T | C | 1.02 | 0.98 | 1.06 | 0.35 | 0.95 | 0.89 | 1.02 | 0.20 | 1.00 | 0.93 | 1.07 | 0.96 | 1.03 | 0.93 | 1.15 | 0.53 | 0.98 | 0.89 | 1.08 | 0.73 |
| rs7138803 | 12 | 50247468 | A | G | 1.00 | 0.98 | 1.03 | 0.81 | 1.03 | 0.97 | 1.08 | 0.31 | 0.96 | 0.91 | 1.02 | 0.16 | 1.02 | 0.92 | 1.13 | 0.72 | 1.01 | 0.96 | 1.07 | 0.71 |
| rs4771122 | 13 | 28020180 | G | A | 0.98 | 0.94 | 1.02 | 0.29 | 0.94 | 0.91 | 0.97 | <0.001 | 1.04 | 0.96 | 1.12 | 0.33 | 0.92 | 0.85 | 1.00 | 0.04 | 0.98 | 0.90 | 1.07 | 0.67 |
| rs11847697 | 14 | 30515112 | T | C | 0.97 | 0.89 | 1.06 | 0.56 | 1.07 | 0.89 | 1.28 | 0.47 | 0.95 | 0.80 | 1.13 | 0.57 | 1.00 | 0.87 | 1.13 | 0.94 | 0.99 | 0.85 | 1.16 | 0.90 |
| rs10150332 | 14 | 79936964 | C | T | 0.98 | 0.91 | 1.05 | 0.53 | 0.94 | 0.89 | 1.00 | 0.03 | 0.97 | 0.89 | 1.06 | 0.52 | 0.93 | 0.86 | 1.01 | 0.07 | 1.02 | 0.93 | 1.13 | 0.66 |
| rs2241423 | 15 | 68086838 | G | A | 1.04 | 0.97 | 1.10 | 0.27 | 1.04 | 0.96 | 1.13 | 0.29 | 0.99 | 0.88 | 1.11 | 0.81 | 1.10 | 1.04 | 1.16 | <0.001 | 1.01 | 0.87 | 1.16 | 0.93 |
| rs12444979 | 16 | 19933600 | C | T | 0.99 | 0.93 | 1.05 | 0.73 | 0.95 | 0.87 | 1.03 | 0.20 | 1.06 | 0.96 | 1.17 | 0.25 | 0.99 | 0.87 | 1.12 | 0.86 | 1.04 | 0.94 | 1.15 | 0.43 |
| rs7359397 | 16 | 28885659 | T | C | 1.02 | 0.98 | 1.05 | 0.39 | 1.07 | 1.01 | 1.13 | 0.02 | 1.01 | 0.94 | 1.08 | 0.77 | 1.02 | 0.98 | 1.06 | 0.27 | 1.05 | 0.99 | 1.11 | 0.13 |
| rs1558902 | 16 | 53803574 | A | T | 1.01 | 0.96 | 1.06 | 0.69 | 1.05 | 0.98 | 1.13 | 0.17 | 1.00 | 0.95 | 1.06 | 1.00 | 1.03 | 0.93 | 1.14 | 0.55 | 1.03 | 0.96 | 1.12 | 0.41 |
| rs571312 | 18 | 57839769 | A | C | 0.99 | 0.96 | 1.03 | 0.59 | 0.98 | 0.92 | 1.04 | 0.42 | 1.05 | 0.99 | 1.11 | 0.10 | 1.04 | 0.95 | 1.14 | 0.35 | 0.98 | 0.92 | 1.05 | 0.56 |
| rs29941 | 19 | 34309532 | G | A | 0.98 | 0.93 | 1.02 | 0.32 | 0.98 | 0.93 | 1.04 | 0.49 | 1.01 | 0.96 | 1.07 | 0.74 | 1.06 | 0.99 | 1.14 | 0.11 | 0.97 | 0.91 | 1.05 | 0.45 |
| rs2287019 | 19 | 46202172 | C | T | 0.99 | 0.93 | 1.05 | 0.78 | 1.02 | 0.92 | 1.12 | 0.73 | 0.98 | 0.89 | 1.09 | 0.76 | 1.10 | 0.98 | 1.24 | 0.09 | 0.95 | 0.87 | 1.04 | 0.25 |
| rs3810291 | 19 | 47569003 | A | G | 1.03 | 0.99 | 1.08 | 0.19 | 1.05 | 0.99 | 1.12 | 0.12 | 1.03 | 0.96 | 1.11 | 0.35 | 1.04 | 0.96 | 1.14 | 0.33 | 1.03 | 0.97 | 1.08 | 0.34 |

Notes: *Hazard ratios are adjusted for the first 8 principal components of population stratification.*

| **Supplementary Table 8: Association of 179 individual height SNPs with prostate cancer specific mortality after prostate cancer diagnosis. Hazard ratios indicate the effect of one extra height increasing allele.** | | | | | | | | | | | | | | | | | | | | | | | | |  |
| --- | --- | --- | --- | --- | --- | --- | --- | --- | --- | --- | --- | --- | --- | --- | --- | --- | --- | --- | --- | --- | --- | --- | --- | --- | --- |
|  |  |  | Alleles | | Any prostate cancer (N=15,491) | | | | Localised prostate cancer (N=9,100) | | | | Advanced prostate cancer (N=3,685) | | | | Low grade prostate cancer (N=6,026) | | | | High grade prostate cancer (N=6,301) | | | |  |
|  |  |  | Height |  | Hazard | Confidence interval | | p- | Hazard | Confidence interval | | p- | Hazard | Confidence interval | | p- | Hazard | Confidence interval | | p- | Hazard | Confidence interval | | p- |  |
| Rs ID | Chr. | Position | increasing | Other | ratio | Lower | Upper | value | ratio | Lower | Upper | value | ratio | Lower | Upper | value | ratio | Lower | Upper | value | ratio | Lower | Upper | value |  |
| rs425277 | 1 | 2069172 | T | C | 0.96 | 0.90 | 1.03 | 0.29 | 1.01 | 0.91 | 1.12 | 0.81 | 0.94 | 0.89 | 1.01 | 0.08 | 0.97 | 0.90 | 1.06 | 0.53 | 1.00 | 0.93 | 1.08 | 0.99 |  |
| rs2284746 | 1 | 17306675 | G | C | 0.98 | 0.92 | 1.05 | 0.60 | 1.01 | 0.91 | 1.12 | 0.85 | 1.01 | 0.94 | 1.08 | 0.88 | 1.06 | 0.95 | 1.19 | 0.27 | 1.02 | 0.93 | 1.11 | 0.74 |  |
| rs1738475 | 1 | 23536891 | C | G | 1.04 | 1.00 | 1.09 | 0.06 | 1.12 | 0.99 | 1.25 | 0.06 | 0.98 | 0.92 | 1.05 | 0.65 | 1.14 | 1.03 | 1.27 | 0.01 | 1.04 | 0.95 | 1.13 | 0.38 |  |
| rs4601530 | 1 | 25044111 | C | T | 1.01 | 0.92 | 1.10 | 0.86 | 1.02 | 0.87 | 1.19 | 0.81 | 1.01 | 0.93 | 1.10 | 0.80 | 1.10 | 1.01 | 1.20 | 0.03 | 0.96 | 0.82 | 1.11 | 0.56 |  |
| rs7532866 | 1 | 26741544 | A | G | 1.02 | 0.98 | 1.06 | 0.42 | 1.07 | 0.96 | 1.19 | 0.21 | 0.99 | 0.93 | 1.06 | 0.78 | 1.11 | 1.01 | 1.22 | 0.03 | 1.00 | 0.91 | 1.09 | 0.98 |  |
| rs2154319 | 1 | 41745770 | C | T | 0.98 | 0.93 | 1.04 | 0.55 | 1.03 | 0.91 | 1.16 | 0.66 | 0.96 | 0.90 | 1.01 | 0.14 | 0.98 | 0.89 | 1.08 | 0.64 | 0.94 | 0.87 | 1.02 | 0.13 |  |
| rs17391694 | 1 | 78623626 | T | C | 0.99 | 0.89 | 1.10 | 0.84 | 1.10 | 0.90 | 1.35 | 0.35 | 0.96 | 0.82 | 1.12 | 0.61 | 1.00 | 0.76 | 1.31 | 0.99 | 0.88 | 0.80 | 0.97 | 0.01 |  |
| rs6699417 | 1 | 89123443 | T | C | 0.93 | 0.89 | 0.98 | 0.006 | 0.86 | 0.74 | 0.99 | 0.04 | 0.98 | 0.92 | 1.05 | 0.63 | 0.91 | 0.86 | 0.97 | 0.003 | 0.94 | 0.91 | 0.97 | <0.001 |  |
| rs10874746 | 1 | 93323971 | C | T | 0.95 | 0.90 | 1.01 | 0.12 | 0.98 | 0.86 | 1.12 | 0.79 | 0.90 | 0.86 | 0.95 | <0.001 | 1.00 | 0.90 | 1.11 | 0.98 | 0.92 | 0.88 | 0.95 | <0.001 |  |
| rs9428104 | 1 | 118855587 | G | A | 0.99 | 0.96 | 1.01 | 0.34 | 1.03 | 0.94 | 1.14 | 0.51 | 0.95 | 0.89 | 1.01 | 0.08 | 1.00 | 0.84 | 1.19 | 0.99 | 0.96 | 0.92 | 1.01 | 0.12 |  |
| rs11205277 | 1 | 149892872 | G | A | 1.01 | 0.96 | 1.07 | 0.65 | 1.01 | 0.92 | 1.11 | 0.86 | 1.04 | 0.93 | 1.16 | 0.45 | 1.20 | 1.11 | 1.31 | <0.001 | 0.94 | 0.84 | 1.05 | 0.29 |  |
| rs17346452 | 1 | 172053287 | C | T | 1.04 | 1.00 | 1.08 | 0.04 | 0.99 | 0.89 | 1.09 | 0.80 | 1.03 | 0.98 | 1.09 | 0.23 | 0.96 | 0.78 | 1.17 | 0.66 | 1.02 | 0.98 | 1.06 | 0.25 |  |
| rs1325598 | 1 | 176792249 | G | A | 1.01 | 0.98 | 1.04 | 0.45 | 1.05 | 0.99 | 1.12 | 0.13 | 1.05 | 0.99 | 1.11 | 0.10 | 1.03 | 0.93 | 1.15 | 0.54 | 0.98 | 0.97 | 1.00 | 0.10 |  |
| rs1046934 | 1 | 184023529 | C | A | 1.05 | 0.98 | 1.13 | 0.16 | 1.07 | 0.90 | 1.26 | 0.43 | 1.02 | 0.90 | 1.16 | 0.73 | 1.02 | 0.87 | 1.20 | 0.81 | 1.11 | 1.01 | 1.22 | 0.02 |  |
| rs10863936 | 1 | 212237798 | G | A | 1.04 | 0.98 | 1.09 | 0.17 | 1.09 | 0.97 | 1.22 | 0.13 | 0.98 | 0.93 | 1.04 | 0.56 | 1.04 | 0.95 | 1.14 | 0.38 | 1.04 | 0.98 | 1.10 | 0.25 |  |
| rs6684205 | 1 | 218609702 | G | A | 1.10 | 1.07 | 1.13 | <0.001 | 1.09 | 0.96 | 1.23 | 0.19 | 1.06 | 1.01 | 1.13 | 0.03 | 0.99 | 0.79 | 1.25 | 0.96 | 1.07 | 1.00 | 1.15 | 0.04 |  |
| rs11118346 | 1 | 219743719 | C | T | 0.96 | 0.91 | 1.02 | 0.20 | 0.97 | 0.88 | 1.07 | 0.54 | 0.97 | 0.90 | 1.06 | 0.52 | 0.94 | 0.88 | 1.02 | 0.14 | 0.98 | 0.89 | 1.08 | 0.69 |  |
| rs10799445 | 1 | 227911883 | A | C | 0.99 | 0.95 | 1.04 | 0.68 | 1.00 | 0.89 | 1.12 | 0.97 | 0.98 | 0.88 | 1.10 | 0.72 | 0.85 | 0.76 | 0.96 | 0.008 | 0.96 | 0.91 | 1.01 | 0.14 |  |
| rs4665736 | 2 | 25187599 | T | C | 0.93 | 0.90 | 0.97 | <0.001 | 0.90 | 0.86 | 0.94 | <0.001 | 0.98 | 0.92 | 1.04 | 0.48 | 0.98 | 0.89 | 1.08 | 0.66 | 0.94 | 0.89 | 1.00 | 0.04 |  |
| rs6714546 | 2 | 33361425 | G | A | 1.07 | 0.98 | 1.16 | 0.12 | 1.19 | 1.04 | 1.36 | 0.009 | 1.11 | 1.03 | 1.18 | 0.003 | 1.14 | 0.89 | 1.45 | 0.31 | 1.24 | 1.09 | 1.40 | 0.001 |  |
| rs17511102 | 2 | 37960613 | T | A | 1.00 | 0.92 | 1.08 | 0.93 | 0.90 | 0.69 | 1.17 | 0.45 | 1.07 | 0.96 | 1.18 | 0.21 | 0.87 | 0.69 | 1.11 | 0.28 | 1.17 | 0.98 | 1.38 | 0.08 |  |
| rs2341459 | 2 | 44768202 | T | C | 1.00 | 0.95 | 1.06 | 0.88 | 1.04 | 0.91 | 1.18 | 0.58 | 1.02 | 0.96 | 1.08 | 0.48 | 0.90 | 0.81 | 1.01 | 0.07 | 1.02 | 0.92 | 1.12 | 0.72 |  |
| rs12474201 | 2 | 46921285 | A | G | 0.99 | 0.94 | 1.04 | 0.58 | 1.06 | 0.91 | 1.23 | 0.45 | 0.96 | 0.91 | 1.01 | 0.09 | 1.04 | 0.90 | 1.22 | 0.57 | 0.94 | 0.88 | 1.01 | 0.10 |  |
| rs3791675 | 2 | 56111309 | C | T | 0.94 | 0.87 | 1.01 | 0.11 | 0.95 | 0.84 | 1.07 | 0.40 | 0.91 | 0.83 | 1.01 | 0.07 | 0.87 | 0.77 | 0.98 | 0.02 | 0.99 | 0.86 | 1.13 | 0.84 |  |
| rs11684404 | 2 | 88924622 | C | T | 1.00 | 0.93 | 1.08 | 0.98 | 1.03 | 0.99 | 1.08 | 0.15 | 0.91 | 0.84 | 0.98 | 0.010 | 1.01 | 0.92 | 1.11 | 0.83 | 1.00 | 0.95 | 1.06 | 0.90 |  |
| rs7567288 | 2 | 134434824 | C | T | 0.94 | 0.82 | 1.08 | 0.38 | 1.01 | 0.87 | 1.16 | 0.94 | 0.97 | 0.87 | 1.07 | 0.50 | 0.81 | 0.65 | 1.02 | 0.07 | 1.02 | 0.91 | 1.13 | 0.74 |  |
| rs7567851 | 2 | 178684720 | C | G | 1.12 | 1.06 | 1.19 | <0.001 | 1.22 | 1.07 | 1.39 | 0.002 | 1.09 | 0.95 | 1.25 | 0.24 | 1.39 | 1.16 | 1.66 | <0.001 | 1.04 | 0.93 | 1.16 | 0.52 |  |
| rs1351164 | 2 | 218271898 | T | C | 0.94 | 0.87 | 1.02 | 0.16 | 1.00 | 0.89 | 1.11 | 0.94 | 0.99 | 0.88 | 1.13 | 0.92 | 0.98 | 0.88 | 1.09 | 0.66 | 1.01 | 0.89 | 1.15 | 0.88 |  |
| rs12470505 | 2 | 219908369 | T | G | 1.03 | 0.95 | 1.11 | 0.49 | 0.93 | 0.75 | 1.15 | 0.50 | 1.19 | 1.06 | 1.33 | 0.003 | 1.05 | 0.87 | 1.28 | 0.60 | 1.02 | 0.94 | 1.11 | 0.58 |  |
| rs2629046 | 2 | 225047744 | T | C | 1.03 | 0.99 | 1.08 | 0.11 | 1.05 | 0.99 | 1.12 | 0.11 | 0.98 | 0.90 | 1.06 | 0.62 | 1.20 | 1.05 | 1.36 | 0.007 | 0.96 | 0.89 | 1.04 | 0.34 |  |
| rs2580816 | 2 | 232797966 | C | T | 1.09 | 1.03 | 1.16 | 0.006 | 1.06 | 0.94 | 1.20 | 0.34 | 1.01 | 0.93 | 1.10 | 0.86 | 1.04 | 0.91 | 1.20 | 0.57 | 1.05 | 0.95 | 1.15 | 0.38 |  |
| rs12694997 | 2 | 242262986 | G | A | 0.96 | 0.90 | 1.03 | 0.28 | 0.96 | 0.86 | 1.07 | 0.46 | 0.99 | 0.94 | 1.03 | 0.54 | 0.84 | 0.70 | 1.01 | 0.07 | 1.03 | 0.96 | 1.09 | 0.44 |  |
| rs2597513 | 3 | 13555836 | C | T | 0.90 | 0.81 | 1.00 | 0.04 | 1.16 | 0.95 | 1.41 | 0.15 | 0.83 | 0.76 | 0.90 | <0.001 | 0.83 | 0.62 | 1.11 | 0.20 | 0.94 | 0.86 | 1.04 | 0.24 |  |
| rs13088462 | 3 | 51071713 | C | T | 1.01 | 0.90 | 1.15 | 0.83 | 0.99 | 0.67 | 1.45 | 0.95 | 1.01 | 0.86 | 1.17 | 0.94 | 1.41 | 1.23 | 1.60 | <0.001 | 0.92 | 0.82 | 1.02 | 0.10 |  |
| rs2336725 | 3 | 53118739 | C | T | 1.02 | 0.98 | 1.07 | 0.33 | 1.02 | 0.93 | 1.13 | 0.64 | 1.04 | 0.96 | 1.11 | 0.36 | 1.03 | 0.93 | 1.14 | 0.57 | 1.08 | 1.00 | 1.15 | 0.05 |  |
| rs9835332 | 3 | 56667682 | G | C | 1.00 | 0.92 | 1.09 | 0.99 | 0.95 | 0.87 | 1.03 | 0.23 | 0.98 | 0.89 | 1.08 | 0.70 | 1.02 | 0.91 | 1.16 | 0.71 | 0.98 | 0.89 | 1.09 | 0.75 |  |
| rs17806888 | 3 | 67416322 | T | C | 1.02 | 0.93 | 1.13 | 0.62 | 1.03 | 0.83 | 1.26 | 0.80 | 1.00 | 0.93 | 1.08 | 0.98 | 1.04 | 0.92 | 1.17 | 0.55 | 1.02 | 0.92 | 1.13 | 0.65 |  |
| rs9863706 | 3 | 72437413 | C | T | 1.01 | 0.94 | 1.09 | 0.79 | 0.97 | 0.75 | 1.28 | 0.85 | 1.05 | 0.96 | 1.14 | 0.30 | 1.03 | 0.88 | 1.20 | 0.70 | 0.98 | 0.93 | 1.03 | 0.36 |  |
| rs6439167 | 3 | 129050756 | C | T | 0.87 | 0.79 | 0.96 | 0.004 | 0.92 | 0.79 | 1.07 | 0.26 | 0.88 | 0.81 | 0.96 | 0.003 | 0.84 | 0.64 | 1.10 | 0.20 | 0.86 | 0.79 | 0.93 | <0.001 |  |
| rs9844666 | 3 | 135974216 | G | A | 1.00 | 0.95 | 1.05 | 0.97 | 0.88 | 0.76 | 1.01 | 0.07 | 1.06 | 1.00 | 1.14 | 0.06 | 0.92 | 0.80 | 1.05 | 0.21 | 1.08 | 1.01 | 1.15 | 0.03 |  |
| rs724016 | 3 | 141105570 | G | A | 0.93 | 0.90 | 0.96 | <0.001 | 0.97 | 0.90 | 1.04 | 0.42 | 0.95 | 0.90 | 1.00 | 0.04 | 0.90 | 0.83 | 0.98 | 0.01 | 0.94 | 0.89 | 0.99 | 0.03 |  |
| rs572169 | 3 | 172165727 | T | C | 1.04 | 0.97 | 1.10 | 0.26 | 1.06 | 0.96 | 1.18 | 0.23 | 1.03 | 0.91 | 1.17 | 0.60 | 1.14 | 0.93 | 1.42 | 0.21 | 0.99 | 0.89 | 1.09 | 0.76 |  |
| rs720390 | 3 | 185548683 | A | G | 1.03 | 0.98 | 1.07 | 0.27 | 1.00 | 0.90 | 1.10 | 0.94 | 1.02 | 0.95 | 1.10 | 0.55 | 1.12 | 1.02 | 1.22 | 0.01 | 0.96 | 0.87 | 1.07 | 0.47 |  |
| rs2247341 | 4 | 1701317 | A | G | 0.99 | 0.93 | 1.05 | 0.73 | 0.90 | 0.78 | 1.05 | 0.19 | 1.01 | 0.94 | 1.09 | 0.73 | 1.01 | 0.83 | 1.23 | 0.92 | 1.01 | 0.91 | 1.12 | 0.80 |  |
| rs6449353 | 4 | 18033488 | T | C | 0.98 | 0.88 | 1.10 | 0.78 | 1.05 | 0.81 | 1.36 | 0.73 | 0.94 | 0.86 | 1.02 | 0.14 | 1.11 | 0.96 | 1.29 | 0.17 | 0.98 | 0.82 | 1.18 | 0.87 |  |
| rs17081935 | 4 | 57823476 | T | C | 1.03 | 0.96 | 1.10 | 0.43 | 1.10 | 1.01 | 1.20 | 0.03 | 1.00 | 0.93 | 1.08 | 0.98 | 1.18 | 1.09 | 1.28 | <0.001 | 1.02 | 0.96 | 1.08 | 0.49 |  |
| rs7697556 | 4 | 73515313 | T | C | 0.96 | 0.90 | 1.03 | 0.26 | 0.96 | 0.90 | 1.03 | 0.28 | 0.94 | 0.90 | 0.99 | 0.02 | 0.96 | 0.87 | 1.06 | 0.46 | 0.95 | 0.90 | 1.00 | 0.06 |  |
| rs788867 | 4 | 82150006 | G | T | 1.05 | 1.02 | 1.09 | <0.001 | 1.11 | 1.02 | 1.22 | 0.02 | 1.05 | 0.98 | 1.13 | 0.18 | 1.11 | 1.02 | 1.22 | 0.02 | 1.00 | 0.95 | 1.04 | 0.89 |  |
| rs10010325 | 4 | 106106353 | A | C | 1.01 | 0.95 | 1.07 | 0.80 | 1.03 | 0.97 | 1.10 | 0.29 | 1.02 | 0.96 | 1.08 | 0.56 | 1.12 | 0.99 | 1.26 | 0.08 | 0.95 | 0.83 | 1.09 | 0.48 |  |
| rs7689420 | 4 | 145568352 | C | T | 1.05 | 0.97 | 1.13 | 0.21 | 1.13 | 1.07 | 1.19 | <0.001 | 1.11 | 1.03 | 1.19 | 0.007 | 1.17 | 0.97 | 1.40 | 0.10 | 1.13 | 1.01 | 1.25 | 0.03 |  |
| rs955748 | 4 | 184215675 | G | A | 0.96 | 0.90 | 1.02 | 0.19 | 0.92 | 0.82 | 1.04 | 0.19 | 1.04 | 0.97 | 1.11 | 0.32 | 1.15 | 0.99 | 1.34 | 0.08 | 0.99 | 0.92 | 1.06 | 0.78 |  |
| rs1173727 | 5 | 32830521 | T | C | 0.96 | 0.93 | 1.00 | 0.05 | 0.96 | 0.85 | 1.09 | 0.53 | 1.04 | 0.96 | 1.14 | 0.34 | 1.02 | 0.94 | 1.11 | 0.60 | 0.96 | 0.90 | 1.02 | 0.20 |  |
| rs11958779 | 5 | 55001899 | G | A | 0.99 | 0.94 | 1.05 | 0.71 | 1.08 | 0.97 | 1.20 | 0.16 | 0.93 | 0.88 | 0.99 | 0.02 | 0.99 | 0.82 | 1.21 | 0.96 | 1.02 | 0.94 | 1.10 | 0.66 |  |
| rs10037512 | 5 | 88354675 | T | C | 1.00 | 0.95 | 1.06 | 1.00 | 0.88 | 0.82 | 0.95 | <0.001 | 1.04 | 0.98 | 1.10 | 0.22 | 0.98 | 0.88 | 1.10 | 0.76 | 1.00 | 0.90 | 1.10 | 0.92 |  |
| rs13177718 | 5 | 108113344 | C | T | 0.93 | 0.84 | 1.02 | 0.12 | 0.89 | 0.67 | 1.20 | 0.45 | 0.96 | 0.88 | 1.05 | 0.37 | 0.92 | 0.69 | 1.23 | 0.57 | 0.96 | 0.87 | 1.06 | 0.40 |  |
| rs1582931 | 5 | 122657199 | G | A | 0.97 | 0.94 | 1.01 | 0.14 | 0.94 | 0.84 | 1.06 | 0.33 | 1.05 | 0.99 | 1.12 | 0.10 | 0.96 | 0.81 | 1.13 | 0.64 | 0.98 | 0.92 | 1.04 | 0.49 |  |
| rs274546 | 5 | 131699867 | G | A | 0.96 | 0.92 | 1.01 | 0.09 | 1.02 | 0.92 | 1.14 | 0.69 | 0.93 | 0.86 | 1.00 | 0.04 | 0.97 | 0.88 | 1.06 | 0.49 | 0.94 | 0.90 | 0.98 | 0.008 |  |
| rs526896 | 5 | 134356705 | T | G | 0.96 | 0.91 | 1.02 | 0.22 | 0.96 | 0.86 | 1.07 | 0.45 | 0.96 | 0.92 | 1.01 | 0.13 | 1.03 | 0.88 | 1.20 | 0.72 | 0.94 | 0.89 | 0.98 | 0.007 |  |
| rs4282339 | 5 | 168256240 | G | A | 1.07 | 1.02 | 1.12 | 0.007 | 1.17 | 1.06 | 1.29 | 0.002 | 1.02 | 0.94 | 1.10 | 0.67 | 1.01 | 0.87 | 1.16 | 0.94 | 1.08 | 1.00 | 1.17 | 0.06 |  |
| rs12153391 | 5 | 171203438 | C | A | 0.88 | 0.84 | 0.93 | <0.001 | 0.92 | 0.84 | 0.99 | 0.04 | 0.88 | 0.82 | 0.94 | <0.001 | 0.90 | 0.79 | 1.03 | 0.12 | 0.86 | 0.78 | 0.95 | 0.003 |  |
| rs889014 | 5 | 172984114 | C | T | 1.00 | 0.94 | 1.06 | 0.98 | 0.94 | 0.78 | 1.13 | 0.51 | 1.03 | 0.99 | 1.07 | 0.15 | 0.90 | 0.75 | 1.08 | 0.27 | 1.02 | 0.98 | 1.06 | 0.35 |  |
| rs422421 | 5 | 176517326 | C | T | 1.05 | 0.95 | 1.16 | 0.34 | 1.03 | 0.86 | 1.23 | 0.78 | 1.05 | 1.03 | 1.08 | <0.001 | 1.12 | 0.86 | 1.46 | 0.41 | 1.04 | 0.99 | 1.10 | 0.14 |  |
| rs6879260 | 5 | 179731014 | C | T | 0.98 | 0.91 | 1.05 | 0.60 | 0.92 | 0.86 | 0.97 | 0.006 | 0.99 | 0.93 | 1.06 | 0.86 | 1.02 | 0.89 | 1.16 | 0.83 | 0.98 | 0.83 | 1.15 | 0.76 |  |
| rs3812163 | 6 | 7725760 | T | A | 0.98 | 0.94 | 1.02 | 0.30 | 1.01 | 0.94 | 1.08 | 0.80 | 1.00 | 0.96 | 1.04 | 0.92 | 0.93 | 0.82 | 1.05 | 0.23 | 1.00 | 0.94 | 1.05 | 0.86 |  |
| rs1047014 | 6 | 19841493 | C | T | 1.06 | 1.01 | 1.11 | 0.01 | 1.09 | 0.97 | 1.22 | 0.16 | 1.03 | 0.95 | 1.13 | 0.46 | 1.19 | 1.01 | 1.40 | 0.03 | 0.98 | 0.92 | 1.05 | 0.57 |  |
| rs806794 | 6 | 26200677 | A | G | 0.97 | 0.93 | 1.01 | 0.17 | 0.99 | 0.87 | 1.13 | 0.88 | 0.92 | 0.86 | 0.98 | 0.008 | 0.94 | 0.75 | 1.18 | 0.59 | 0.88 | 0.78 | 1.00 | 0.04 |  |
| rs3129109 | 6 | 29084232 | C | T | 1.08 | 1.00 | 1.16 | 0.05 | 1.06 | 0.91 | 1.24 | 0.43 | 1.09 | 1.02 | 1.18 | 0.02 | 1.04 | 0.91 | 1.19 | 0.52 | 1.08 | 0.94 | 1.24 | 0.30 |  |
| rs114684762 | 6 | 31380529 | A | G | 1.03 | 0.97 | 1.09 | 0.40 | 1.00 | 0.84 | 1.19 | 0.98 | 1.05 | 0.92 | 1.20 | 0.47 | 0.87 | 0.78 | 0.98 | 0.02 | 1.05 | 0.91 | 1.21 | 0.48 |  |
| rs115961701 | 6 | 32663999 | G | C | 0.98 | 0.91 | 1.05 | 0.52 | 0.98 | 0.85 | 1.13 | 0.81 | 0.93 | 0.86 | 1.01 | 0.10 | 0.85 | 0.76 | 0.95 | 0.003 | 0.94 | 0.86 | 1.03 | 0.22 |  |
| rs2780226 | 6 | 34199092 | C | T | 0.96 | 0.87 | 1.05 | 0.37 | 0.91 | 0.73 | 1.14 | 0.41 | 0.95 | 0.81 | 1.11 | 0.51 | 1.10 | 0.91 | 1.34 | 0.33 | 0.91 | 0.81 | 1.02 | 0.10 |  |
| rs6457821 | 6 | 35402805 | C | A | 1.07 | 0.90 | 1.26 | 0.45 | 1.20 | 0.67 | 2.13 | 0.54 | 1.12 | 0.84 | 1.48 | 0.45 | 2.08 | 1.09 | 3.95 | 0.03 | 1.01 | 0.71 | 1.43 | 0.96 |  |
| rs9472414 | 6 | 44946506 | T | A | 0.94 | 0.85 | 1.05 | 0.28 | 0.94 | 0.75 | 1.19 | 0.62 | 1.06 | 0.97 | 1.17 | 0.20 | 0.87 | 0.68 | 1.10 | 0.24 | 0.99 | 0.89 | 1.10 | 0.88 |  |
| rs9360921 | 6 | 76265642 | G | T | 1.13 | 1.00 | 1.27 | 0.04 | 1.16 | 1.03 | 1.32 | 0.02 | 1.09 | 0.89 | 1.33 | 0.42 | 1.04 | 0.71 | 1.51 | 0.84 | 1.07 | 0.92 | 1.23 | 0.38 |  |
| rs310405 | 6 | 81800362 | A | G | 1.00 | 0.96 | 1.03 | 0.78 | 1.08 | 1.00 | 1.18 | 0.05 | 0.99 | 0.88 | 1.12 | 0.89 | 0.91 | 0.84 | 1.00 | 0.04 | 1.07 | 0.99 | 1.15 | 0.07 |  |
| rs7759938 | 6 | 105378954 | T | C | 0.94 | 0.90 | 0.98 | 0.003 | 0.96 | 0.86 | 1.06 | 0.38 | 1.01 | 0.94 | 1.08 | 0.87 | 1.07 | 0.97 | 1.18 | 0.16 | 0.93 | 0.84 | 1.03 | 0.19 |  |
| rs1046943 | 6 | 109783941 | G | A | 1.04 | 0.95 | 1.13 | 0.37 | 1.06 | 0.90 | 1.24 | 0.49 | 1.00 | 0.91 | 1.11 | 0.93 | 0.95 | 0.90 | 1.01 | 0.10 | 1.01 | 0.87 | 1.18 | 0.86 |  |
| rs961764 | 6 | 117522156 | C | G | 1.02 | 0.94 | 1.11 | 0.67 | 0.94 | 0.78 | 1.14 | 0.55 | 1.01 | 0.93 | 1.11 | 0.75 | 0.88 | 0.75 | 1.03 | 0.11 | 0.96 | 0.87 | 1.07 | 0.51 |  |
| rs1490384 | 6 | 126851160 | C | T | 1.00 | 0.96 | 1.04 | 0.92 | 0.94 | 0.83 | 1.06 | 0.32 | 1.00 | 0.93 | 1.07 | 0.98 | 0.99 | 0.84 | 1.17 | 0.90 | 1.06 | 1.00 | 1.11 | 0.04 |  |
| rs6569648 | 6 | 130349119 | C | T | 1.01 | 0.92 | 1.11 | 0.82 | 0.91 | 0.79 | 1.06 | 0.24 | 1.07 | 0.92 | 1.23 | 0.40 | 1.11 | 0.91 | 1.35 | 0.32 | 1.04 | 0.89 | 1.21 | 0.65 |  |
| rs7763064 | 6 | 142797289 | G | A | 1.00 | 0.92 | 1.09 | 0.97 | 0.94 | 0.86 | 1.01 | 0.10 | 1.09 | 1.03 | 1.15 | 0.003 | 1.23 | 0.96 | 1.56 | 0.10 | 0.99 | 0.92 | 1.08 | 0.90 |  |
| rs543650 | 6 | 152110943 | G | T | 1.01 | 0.96 | 1.06 | 0.64 | 0.93 | 0.84 | 1.03 | 0.18 | 1.01 | 0.94 | 1.09 | 0.75 | 1.02 | 0.84 | 1.24 | 0.81 | 1.01 | 0.91 | 1.12 | 0.86 |  |
| rs9456307 | 6 | 158929442 | T | A | 0.99 | 0.86 | 1.14 | 0.89 | 0.92 | 0.65 | 1.30 | 0.63 | 1.06 | 0.92 | 1.24 | 0.41 | 0.87 | 0.59 | 1.29 | 0.50 | 1.09 | 0.90 | 1.32 | 0.39 |  |
| rs798489 | 7 | 2801803 | C | T | 0.96 | 0.89 | 1.04 | 0.29 | 0.96 | 0.79 | 1.16 | 0.66 | 0.93 | 0.79 | 1.09 | 0.36 | 1.17 | 0.92 | 1.48 | 0.20 | 0.94 | 0.85 | 1.04 | 0.24 |  |
| rs4470914 | 7 | 19616522 | T | C | 1.01 | 0.86 | 1.18 | 0.89 | 1.02 | 0.91 | 1.13 | 0.76 | 1.03 | 0.84 | 1.26 | 0.76 | 1.07 | 0.93 | 1.23 | 0.34 | 0.99 | 0.75 | 1.30 | 0.93 |  |
| rs12534093 | 7 | 23502974 | T | A | 0.98 | 0.88 | 1.08 | 0.62 | 0.91 | 0.80 | 1.03 | 0.14 | 0.97 | 0.82 | 1.16 | 0.78 | 1.14 | 0.99 | 1.32 | 0.07 | 0.98 | 0.81 | 1.19 | 0.87 |  |
| rs1708299 | 7 | 28189946 | A | G | 1.02 | 0.96 | 1.08 | 0.50 | 0.93 | 0.79 | 1.10 | 0.41 | 1.02 | 0.98 | 1.07 | 0.31 | 1.07 | 0.91 | 1.27 | 0.41 | 0.95 | 0.85 | 1.05 | 0.32 |  |
| rs6959212 | 7 | 38128326 | C | T | 1.04 | 0.98 | 1.10 | 0.19 | 1.02 | 0.87 | 1.19 | 0.83 | 1.04 | 0.92 | 1.18 | 0.53 | 1.19 | 1.07 | 1.31 | <0.001 | 0.99 | 0.91 | 1.09 | 0.86 |  |
| rs42235 | 7 | 92248076 | T | C | 1.01 | 0.95 | 1.07 | 0.83 | 1.04 | 0.94 | 1.15 | 0.43 | 0.96 | 0.92 | 1.02 | 0.17 | 1.05 | 0.87 | 1.27 | 0.60 | 0.96 | 0.89 | 1.04 | 0.32 |  |
| rs822552 | 7 | 148650634 | G | C | 0.94 | 0.84 | 1.05 | 0.25 | 1.01 | 0.91 | 1.13 | 0.80 | 1.04 | 0.98 | 1.09 | 0.21 | 0.98 | 0.86 | 1.12 | 0.82 | 1.00 | 0.95 | 1.04 | 0.87 |  |
| rs77505230 | 7 | 150517022 | G | C | 1.02 | 0.97 | 1.07 | 0.55 | 1.00 | 0.81 | 1.23 | 0.97 | 1.02 | 0.93 | 1.12 | 0.70 | 1.08 | 0.95 | 1.23 | 0.23 | 0.99 | 0.91 | 1.07 | 0.73 |  |
| rs1013209 | 8 | 24116304 | C | T | 1.04 | 0.96 | 1.13 | 0.31 | 1.01 | 0.88 | 1.17 | 0.87 | 1.11 | 1.04 | 1.19 | 0.003 | 0.97 | 0.75 | 1.25 | 0.81 | 1.15 | 1.01 | 1.30 | 0.03 |  |
| rs7460090 | 8 | 57194163 | T | C | 1.03 | 0.96 | 1.11 | 0.42 | 1.11 | 0.95 | 1.29 | 0.20 | 1.10 | 0.90 | 1.34 | 0.36 | 1.38 | 1.03 | 1.84 | 0.03 | 0.99 | 0.89 | 1.11 | 0.88 |  |
| rs6473015 | 8 | 78178485 | C | A | 0.96 | 0.82 | 1.12 | 0.57 | 0.97 | 0.85 | 1.12 | 0.71 | 0.94 | 0.76 | 1.15 | 0.55 | 0.91 | 0.66 | 1.27 | 0.59 | 1.01 | 0.84 | 1.21 | 0.95 |  |
| rs6470764 | 8 | 130725665 | C | T | 1.07 | 0.99 | 1.15 | 0.07 | 1.13 | 0.99 | 1.29 | 0.08 | 1.03 | 0.93 | 1.14 | 0.58 | 1.16 | 0.90 | 1.48 | 0.25 | 1.03 | 0.95 | 1.12 | 0.46 |  |
| rs12680655 | 8 | 135637337 | C | G | 0.96 | 0.91 | 1.01 | 0.12 | 1.01 | 0.88 | 1.16 | 0.85 | 1.02 | 0.95 | 1.11 | 0.54 | 1.05 | 0.89 | 1.24 | 0.58 | 1.00 | 0.95 | 1.04 | 0.88 |  |
| rs7864648 | 9 | 16368732 | T | G | 1.03 | 0.98 | 1.08 | 0.29 | 1.12 | 0.95 | 1.33 | 0.18 | 1.02 | 0.92 | 1.13 | 0.69 | 1.05 | 0.93 | 1.18 | 0.42 | 1.06 | 0.98 | 1.14 | 0.12 |  |
| rs11144688 | 9 | 78542286 | G | A | 0.99 | 0.88 | 1.11 | 0.85 | 0.97 | 0.83 | 1.13 | 0.67 | 0.97 | 0.85 | 1.11 | 0.68 | 0.85 | 0.54 | 1.34 | 0.48 | 0.97 | 0.82 | 1.15 | 0.74 |  |
| rs7853377 | 9 | 86552205 | G | A | 0.99 | 0.90 | 1.09 | 0.86 | 0.99 | 0.80 | 1.21 | 0.91 | 1.05 | 0.90 | 1.21 | 0.56 | 1.09 | 0.79 | 1.50 | 0.60 | 0.95 | 0.85 | 1.07 | 0.44 |  |
| rs8181166 | 9 | 89116628 | C | G | 0.96 | 0.90 | 1.02 | 0.18 | 0.95 | 0.87 | 1.04 | 0.30 | 0.98 | 0.91 | 1.06 | 0.64 | 1.00 | 0.85 | 1.17 | 0.95 | 0.98 | 0.90 | 1.06 | 0.58 |  |
| rs2778031 | 9 | 90835726 | T | C | 1.14 | 1.06 | 1.23 | <0.001 | 1.16 | 1.06 | 1.28 | 0.002 | 1.14 | 0.99 | 1.30 | 0.06 | 1.13 | 0.94 | 1.36 | 0.19 | 1.12 | 1.02 | 1.23 | 0.02 |  |
| rs9969804 | 9 | 95429120 | A | C | 1.05 | 1.00 | 1.10 | 0.04 | 1.07 | 0.96 | 1.20 | 0.22 | 1.03 | 0.98 | 1.09 | 0.25 | 1.04 | 0.94 | 1.16 | 0.40 | 1.06 | 0.97 | 1.17 | 0.21 |  |
| rs1257763 | 9 | 96893945 | A | G | 0.92 | 0.77 | 1.08 | 0.31 | 1.05 | 0.75 | 1.46 | 0.78 | 0.91 | 0.76 | 1.10 | 0.32 | 0.97 | 0.63 | 1.48 | 0.87 | 1.09 | 0.87 | 1.36 | 0.46 |  |
| rs473902 | 9 | 98256235 | T | G | 1.07 | 0.94 | 1.20 | 0.30 | 1.09 | 0.84 | 1.42 | 0.53 | 1.05 | 0.84 | 1.30 | 0.67 | 1.00 | 0.70 | 1.41 | 0.98 | 0.98 | 0.78 | 1.24 | 0.88 |  |
| rs7027110 | 9 | 109599046 | A | G | 1.03 | 0.98 | 1.08 | 0.26 | 1.18 | 1.06 | 1.30 | 0.002 | 0.99 | 0.92 | 1.06 | 0.73 | 1.09 | 0.89 | 1.33 | 0.42 | 1.05 | 0.96 | 1.14 | 0.30 |  |
| rs1468758 | 9 | 113807082 | C | T | 1.04 | 0.99 | 1.10 | 0.13 | 0.91 | 0.86 | 0.97 | 0.002 | 1.05 | 0.96 | 1.15 | 0.31 | 1.27 | 1.08 | 1.50 | 0.005 | 0.98 | 0.89 | 1.07 | 0.65 |  |
| rs751543 | 9 | 119122342 | T | C | 0.98 | 0.91 | 1.05 | 0.53 | 1.03 | 0.90 | 1.17 | 0.71 | 1.01 | 0.93 | 1.10 | 0.78 | 1.09 | 0.97 | 1.22 | 0.14 | 0.99 | 0.90 | 1.07 | 0.73 |  |
| rs7466269 | 9 | 133464084 | A | G | 0.94 | 0.91 | 0.97 | <0.001 | 0.97 | 0.83 | 1.13 | 0.69 | 0.87 | 0.82 | 0.93 | <0.001 | 0.87 | 0.77 | 0.99 | 0.04 | 0.96 | 0.88 | 1.04 | 0.34 |  |
| rs7849585 | 9 | 139111870 | T | G | 0.96 | 0.90 | 1.03 | 0.30 | 0.90 | 0.65 | 1.25 | 0.54 | 0.99 | 0.94 | 1.05 | 0.78 | 1.16 | 1.04 | 1.29 | 0.006 | 0.96 | 0.86 | 1.07 | 0.46 |  |
| rs7909670 | 10 | 12918764 | C | T | 0.97 | 0.93 | 1.02 | 0.21 | 1.00 | 0.90 | 1.10 | 0.95 | 0.93 | 0.82 | 1.05 | 0.23 | 1.00 | 0.88 | 1.14 | 0.99 | 0.95 | 0.84 | 1.06 | 0.34 |  |
| rs2145998 | 10 | 81121696 | T | A | 0.97 | 0.93 | 1.02 | 0.24 | 0.94 | 0.78 | 1.12 | 0.48 | 0.99 | 0.89 | 1.09 | 0.82 | 0.98 | 0.87 | 1.12 | 0.81 | 0.97 | 0.91 | 1.03 | 0.29 |  |
| rs11599750 | 10 | 101805442 | C | T | 0.94 | 0.90 | 0.99 | 0.02 | 0.97 | 0.88 | 1.07 | 0.55 | 0.94 | 0.89 | 0.99 | 0.02 | 1.10 | 1.01 | 1.18 | 0.02 | 0.93 | 0.88 | 0.98 | 0.009 |  |
| rs2237886 | 11 | 2810731 | T | C | 0.95 | 0.89 | 1.01 | 0.13 | 1.00 | 0.86 | 1.16 | 0.98 | 0.89 | 0.82 | 0.98 | 0.01 | 1.10 | 0.89 | 1.37 | 0.37 | 0.95 | 0.86 | 1.05 | 0.34 |  |
| rs7926971 | 11 | 12698040 | G | A | 0.97 | 0.92 | 1.02 | 0.19 | 1.07 | 0.94 | 1.22 | 0.33 | 0.90 | 0.83 | 0.97 | 0.008 | 0.93 | 0.79 | 1.10 | 0.41 | 0.99 | 0.91 | 1.09 | 0.91 |  |
| rs1330 | 11 | 17316029 | T | C | 0.99 | 0.95 | 1.03 | 0.64 | 1.06 | 0.98 | 1.13 | 0.13 | 0.98 | 0.94 | 1.03 | 0.44 | 0.93 | 0.79 | 1.10 | 0.39 | 0.93 | 0.87 | 0.98 | 0.01 |  |
| rs79890777 | 11 | 48098280 | G | A | 1.00 | 0.95 | 1.06 | 0.86 | 1.06 | 0.92 | 1.23 | 0.43 | 1.04 | 0.97 | 1.11 | 0.30 | 1.17 | 0.94 | 1.46 | 0.16 | 1.02 | 0.92 | 1.14 | 0.69 |  |
| rs1814175 | 11 | 49559172 | T | C | 1.04 | 1.00 | 1.08 | 0.06 | 1.04 | 0.96 | 1.12 | 0.33 | 1.03 | 0.98 | 1.09 | 0.21 | 1.16 | 1.00 | 1.34 | 0.05 | 1.05 | 0.92 | 1.19 | 0.48 |  |
| rs3782089 | 11 | 65336819 | C | T | 1.02 | 0.95 | 1.10 | 0.57 | 1.03 | 0.84 | 1.25 | 0.79 | 1.03 | 0.87 | 1.21 | 0.76 | 0.86 | 0.72 | 1.04 | 0.11 | 1.00 | 0.84 | 1.20 | 0.99 |  |
| rs7112925 | 11 | 66826160 | C | T | 1.00 | 0.93 | 1.08 | 0.94 | 1.13 | 1.02 | 1.24 | 0.01 | 0.92 | 0.86 | 0.99 | 0.02 | 1.08 | 0.99 | 1.18 | 0.07 | 0.94 | 0.87 | 1.02 | 0.12 |  |
| rs634552 | 11 | 75282052 | T | G | 1.07 | 0.99 | 1.15 | 0.09 | 1.21 | 1.07 | 1.38 | 0.003 | 1.03 | 0.91 | 1.15 | 0.66 | 1.26 | 1.09 | 1.46 | 0.002 | 1.06 | 0.99 | 1.14 | 0.11 |  |
| rs494459 | 11 | 118574675 | T | C | 1.01 | 0.95 | 1.07 | 0.87 | 1.04 | 0.86 | 1.27 | 0.68 | 1.04 | 0.99 | 1.09 | 0.14 | 1.01 | 0.92 | 1.11 | 0.79 | 0.99 | 0.92 | 1.06 | 0.74 |  |
| rs654723 | 11 | 128586155 | A | C | 1.03 | 0.99 | 1.07 | 0.10 | 1.01 | 0.92 | 1.11 | 0.87 | 1.14 | 1.08 | 1.21 | <0.001 | 0.93 | 0.86 | 1.00 | 0.05 | 1.12 | 1.08 | 1.17 | <0.001 |  |
| rs2856321 | 12 | 11855773 | G | A | 1.02 | 0.98 | 1.07 | 0.35 | 1.06 | 0.97 | 1.17 | 0.20 | 1.00 | 0.95 | 1.05 | 0.87 | 1.06 | 0.93 | 1.22 | 0.39 | 0.99 | 0.95 | 1.04 | 0.72 |  |
| rs10770705 | 12 | 20857467 | A | C | 1.03 | 0.98 | 1.09 | 0.23 | 0.91 | 0.76 | 1.10 | 0.33 | 1.08 | 1.00 | 1.15 | 0.04 | 1.17 | 0.92 | 1.49 | 0.19 | 0.95 | 0.87 | 1.03 | 0.21 |  |
| rs2638953 | 12 | 28534415 | C | G | 0.98 | 0.94 | 1.02 | 0.40 | 0.95 | 0.86 | 1.05 | 0.29 | 1.04 | 0.95 | 1.14 | 0.36 | 1.09 | 0.90 | 1.32 | 0.36 | 0.97 | 0.88 | 1.07 | 0.57 |  |
| rs2066807 | 12 | 56740682 | G | C | 0.96 | 0.88 | 1.05 | 0.35 | 0.84 | 0.65 | 1.08 | 0.17 | 1.06 | 0.91 | 1.24 | 0.45 | 0.91 | 0.67 | 1.23 | 0.53 | 0.92 | 0.78 | 1.08 | 0.31 |  |
| rs1351394 | 12 | 66351826 | T | C | 0.98 | 0.90 | 1.06 | 0.65 | 0.95 | 0.82 | 1.11 | 0.54 | 1.03 | 0.97 | 1.09 | 0.33 | 0.97 | 0.83 | 1.13 | 0.66 | 0.99 | 0.94 | 1.04 | 0.73 |  |
| rs10748128 | 12 | 69827658 | T | G | 1.06 | 1.00 | 1.11 | 0.04 | 0.98 | 0.93 | 1.03 | 0.42 | 1.12 | 1.07 | 1.18 | <0.001 | 1.11 | 0.95 | 1.29 | 0.18 | 1.04 | 0.96 | 1.14 | 0.31 |  |
| rs11107116 | 12 | 93978504 | T | G | 1.08 | 1.02 | 1.14 | 0.008 | 1.12 | 0.97 | 1.29 | 0.13 | 1.04 | 0.98 | 1.10 | 0.20 | 1.03 | 0.91 | 1.15 | 0.65 | 1.11 | 1.04 | 1.18 | 0.002 |  |
| rs7971536 | 12 | 102373788 | T | A | 0.98 | 0.90 | 1.07 | 0.69 | 1.02 | 0.87 | 1.20 | 0.82 | 0.96 | 0.88 | 1.04 | 0.32 | 0.90 | 0.69 | 1.18 | 0.45 | 1.02 | 0.91 | 1.14 | 0.77 |  |
| rs11830103 | 12 | 123823546 | G | A | 1.05 | 0.95 | 1.17 | 0.32 | 1.07 | 0.90 | 1.28 | 0.46 | 1.09 | 0.95 | 1.26 | 0.23 | 0.98 | 0.84 | 1.13 | 0.77 | 1.02 | 0.95 | 1.11 | 0.55 |  |
| rs7332115 | 13 | 33147548 | G | T | 1.03 | 0.94 | 1.12 | 0.59 | 1.05 | 0.90 | 1.22 | 0.52 | 0.94 | 0.86 | 1.03 | 0.19 | 1.06 | 0.90 | 1.24 | 0.50 | 1.04 | 0.97 | 1.11 | 0.29 |  |
| rs3118905 | 13 | 51105334 | G | A | 1.07 | 0.97 | 1.17 | 0.19 | 0.99 | 0.83 | 1.16 | 0.87 | 1.07 | 1.00 | 1.13 | 0.04 | 1.13 | 0.93 | 1.36 | 0.21 | 1.00 | 0.92 | 1.09 | 0.96 |  |
| rs7319045 | 13 | 92024574 | A | G | 0.99 | 0.89 | 1.10 | 0.89 | 1.10 | 0.94 | 1.28 | 0.22 | 0.98 | 0.89 | 1.08 | 0.69 | 0.90 | 0.67 | 1.22 | 0.51 | 1.02 | 0.96 | 1.09 | 0.53 |  |
| rs1950500 | 14 | 24830850 | T | C | 0.98 | 0.95 | 1.01 | 0.30 | 1.04 | 0.95 | 1.13 | 0.43 | 0.96 | 0.90 | 1.03 | 0.27 | 1.06 | 0.80 | 1.41 | 0.68 | 1.09 | 1.03 | 1.16 | 0.003 |  |
| rs2093210 | 14 | 60957279 | C | T | 1.02 | 0.94 | 1.10 | 0.62 | 1.00 | 0.85 | 1.17 | 0.99 | 1.04 | 0.96 | 1.13 | 0.38 | 1.05 | 0.86 | 1.27 | 0.65 | 0.91 | 0.84 | 0.99 | 0.02 |  |
| rs1570106 | 14 | 68813115 | C | T | 1.00 | 0.93 | 1.09 | 0.91 | 0.94 | 0.87 | 1.01 | 0.08 | 1.09 | 0.99 | 1.19 | 0.08 | 0.95 | 0.85 | 1.06 | 0.37 | 1.08 | 0.93 | 1.24 | 0.31 |  |
| rs862034 | 14 | 74990746 | G | A | 1.01 | 0.94 | 1.09 | 0.74 | 1.05 | 0.89 | 1.24 | 0.57 | 1.03 | 0.94 | 1.13 | 0.59 | 1.18 | 1.00 | 1.39 | 0.05 | 1.03 | 0.93 | 1.14 | 0.54 |  |
| rs7155279 | 14 | 92485881 | G | T | 1.03 | 0.98 | 1.08 | 0.28 | 0.98 | 0.89 | 1.08 | 0.69 | 1.09 | 1.01 | 1.18 | 0.03 | 1.05 | 0.81 | 1.37 | 0.71 | 1.01 | 0.96 | 1.07 | 0.61 |  |
| rs16964211 | 15 | 51530495 | G | A | 1.04 | 0.90 | 1.19 | 0.62 | 0.92 | 0.54 | 1.54 | 0.75 | 1.05 | 0.86 | 1.27 | 0.65 | 1.14 | 0.72 | 1.80 | 0.59 | 1.07 | 0.95 | 1.21 | 0.25 |  |
| rs7178424 | 15 | 62380259 | C | T | 0.99 | 0.90 | 1.08 | 0.81 | 0.96 | 0.89 | 1.02 | 0.21 | 1.05 | 0.97 | 1.14 | 0.22 | 0.93 | 0.86 | 1.00 | 0.06 | 1.03 | 0.93 | 1.15 | 0.52 |  |
| rs10152591 | 15 | 70048157 | A | C | 0.93 | 0.86 | 1.02 | 0.12 | 0.96 | 0.82 | 1.11 | 0.56 | 0.88 | 0.81 | 0.95 | 0.001 | 0.95 | 0.76 | 1.20 | 0.69 | 0.98 | 0.89 | 1.09 | 0.74 |  |
| rs12902421 | 15 | 72161403 | C | T | 1.10 | 0.73 | 1.67 | 0.64 | 1.05 | 0.42 | 2.66 | 0.91 | 0.97 | 0.43 | 2.21 | 0.95 | 1.46 | 0.74 | 2.89 | 0.27 | 1.02 | 0.63 | 1.65 | 0.95 |  |
| rs5742915 | 15 | 74336633 | C | T | 1.06 | 1.01 | 1.12 | 0.02 | 1.09 | 0.96 | 1.24 | 0.19 | 1.00 | 0.91 | 1.09 | 0.97 | 1.11 | 0.98 | 1.25 | 0.09 | 0.97 | 0.88 | 1.06 | 0.53 |  |
| rs11259936 | 15 | 84580582 | C | A | 0.99 | 0.92 | 1.08 | 0.89 | 0.88 | 0.78 | 1.00 | 0.05 | 1.09 | 1.03 | 1.16 | 0.002 | 1.09 | 0.94 | 1.28 | 0.25 | 0.94 | 0.89 | 1.00 | 0.06 |  |
| rs16942341 | 15 | 89388905 | C | T | 1.19 | 1.04 | 1.35 | 0.010 | 1.13 | 0.86 | 1.49 | 0.38 | 1.22 | 0.99 | 1.51 | 0.06 | 1.18 | 0.58 | 2.39 | 0.65 | 1.21 | 1.00 | 1.45 | 0.05 |  |
| rs2871865 | 15 | 99194896 | C | G | 0.96 | 0.88 | 1.04 | 0.31 | 0.86 | 0.73 | 1.01 | 0.07 | 0.95 | 0.84 | 1.08 | 0.46 | 1.02 | 0.75 | 1.38 | 0.89 | 0.97 | 0.84 | 1.13 | 0.72 |  |
| rs4965598 | 15 | 100759614 | C | T | 1.03 | 0.97 | 1.09 | 0.30 | 0.99 | 0.86 | 1.13 | 0.85 | 1.06 | 0.95 | 1.18 | 0.31 | 0.93 | 0.74 | 1.16 | 0.52 | 1.05 | 1.00 | 1.10 | 0.03 |  |
| rs11648796 | 16 | 792190 | G | A | 1.00 | 0.95 | 1.06 | 0.88 | 1.06 | 0.98 | 1.15 | 0.17 | 1.01 | 0.94 | 1.08 | 0.78 | 0.94 | 0.77 | 1.14 | 0.52 | 1.06 | 0.99 | 1.13 | 0.11 |  |
| rs26868 | 16 | 2249376 | A | T | 0.97 | 0.93 | 1.02 | 0.26 | 0.91 | 0.82 | 1.00 | 0.05 | 1.00 | 0.93 | 1.06 | 0.89 | 0.94 | 0.81 | 1.08 | 0.38 | 0.99 | 0.91 | 1.07 | 0.75 |  |
| rs1659127 | 16 | 14388305 | A | G | 0.92 | 0.87 | 0.98 | 0.01 | 0.86 | 0.81 | 0.91 | <0.001 | 0.93 | 0.88 | 0.99 | 0.03 | 0.82 | 0.67 | 1.01 | 0.07 | 0.91 | 0.86 | 0.95 | <0.001 |  |
| rs8052560 | 16 | 88777242 | A | C | 0.99 | 0.91 | 1.07 | 0.76 | 0.98 | 0.74 | 1.31 | 0.91 | 0.96 | 0.92 | 1.01 | 0.14 | 1.05 | 0.91 | 1.21 | 0.51 | 0.99 | 0.89 | 1.10 | 0.88 |  |
| rs4640244 | 17 | 21284223 | A | G | 0.98 | 0.91 | 1.07 | 0.71 | 0.94 | 0.78 | 1.14 | 0.53 | 1.07 | 0.99 | 1.16 | 0.09 | 0.82 | 0.74 | 0.90 | <0.001 | 1.05 | 0.94 | 1.17 | 0.39 |  |
| rs3110496 | 17 | 27917771 | G | A | 0.98 | 0.89 | 1.08 | 0.70 | 0.93 | 0.80 | 1.08 | 0.36 | 1.03 | 0.97 | 1.10 | 0.35 | 0.96 | 0.74 | 1.25 | 0.77 | 1.01 | 0.93 | 1.10 | 0.84 |  |
| rs3764419 | 17 | 29164023 | C | A | 0.96 | 0.90 | 1.03 | 0.28 | 0.99 | 0.87 | 1.13 | 0.93 | 0.94 | 0.87 | 1.02 | 0.15 | 1.04 | 0.90 | 1.21 | 0.57 | 0.94 | 0.84 | 1.05 | 0.28 |  |
| rs17780086 | 17 | 30343282 | A | G | 1.09 | 1.03 | 1.14 | 0.002 | 1.21 | 0.99 | 1.48 | 0.06 | 1.08 | 0.91 | 1.28 | 0.38 | 1.13 | 0.85 | 1.49 | 0.41 | 1.13 | 0.96 | 1.33 | 0.15 |  |
| rs1043515 | 17 | 36922196 | G | A | 1.06 | 0.99 | 1.13 | 0.11 | 1.09 | 0.95 | 1.25 | 0.21 | 1.08 | 1.04 | 1.13 | <0.001 | 0.98 | 0.80 | 1.21 | 0.88 | 1.10 | 0.97 | 1.26 | 0.15 |  |
| rs4986172 | 17 | 43216281 | C | T | 1.01 | 0.95 | 1.07 | 0.76 | 1.09 | 0.95 | 1.25 | 0.22 | 1.04 | 0.99 | 1.09 | 0.09 | 1.07 | 0.99 | 1.15 | 0.08 | 1.02 | 0.92 | 1.12 | 0.75 |  |
| rs2072153 | 17 | 47390014 | C | G | 1.01 | 0.94 | 1.08 | 0.81 | 0.98 | 0.86 | 1.11 | 0.74 | 1.04 | 0.98 | 1.11 | 0.17 | 1.07 | 0.99 | 1.16 | 0.11 | 0.93 | 0.85 | 1.03 | 0.18 |  |
| rs4605213 | 17 | 49244747 | C | G | 1.02 | 0.98 | 1.06 | 0.35 | 0.99 | 0.93 | 1.05 | 0.76 | 1.05 | 0.99 | 1.11 | 0.08 | 1.00 | 0.87 | 1.16 | 0.99 | 1.05 | 1.00 | 1.09 | 0.04 |  |
| rs227724 | 17 | 54778817 | T | A | 0.98 | 0.91 | 1.06 | 0.57 | 1.05 | 0.95 | 1.17 | 0.33 | 0.92 | 0.84 | 1.00 | 0.06 | 0.86 | 0.72 | 1.03 | 0.11 | 1.02 | 0.89 | 1.18 | 0.76 |  |
| rs2079795 | 17 | 59496649 | T | C | 0.95 | 0.89 | 1.02 | 0.13 | 0.88 | 0.73 | 1.06 | 0.17 | 0.98 | 0.89 | 1.08 | 0.67 | 0.95 | 0.88 | 1.03 | 0.18 | 0.92 | 0.78 | 1.10 | 0.36 |  |
| rs2665838 | 17 | 61966465 | G | C | 1.00 | 0.94 | 1.06 | 0.91 | 1.01 | 0.88 | 1.16 | 0.90 | 1.01 | 0.96 | 1.06 | 0.76 | 1.05 | 0.94 | 1.18 | 0.36 | 1.01 | 0.91 | 1.11 | 0.90 |  |
| rs11867479 | 17 | 68090207 | T | C | 0.94 | 0.90 | 0.99 | 0.02 | 1.01 | 0.92 | 1.10 | 0.91 | 0.93 | 0.86 | 1.00 | 0.05 | 1.01 | 0.82 | 1.25 | 0.93 | 0.94 | 0.88 | 1.00 | 0.05 |  |
| rs4800452 | 18 | 20727611 | T | C | 1.08 | 1.05 | 1.12 | <0.001 | 1.10 | 0.98 | 1.23 | 0.11 | 1.13 | 1.02 | 1.24 | 0.01 | 1.16 | 1.04 | 1.30 | 0.007 | 1.06 | 0.99 | 1.12 | 0.09 |  |
| rs9967417 | 18 | 46959500 | G | C | 0.99 | 0.94 | 1.05 | 0.74 | 0.95 | 0.88 | 1.03 | 0.23 | 1.03 | 0.96 | 1.10 | 0.48 | 1.06 | 0.91 | 1.24 | 0.45 | 1.03 | 0.93 | 1.13 | 0.56 |  |
| rs17782313 | 18 | 57851097 | C | T | 0.98 | 0.92 | 1.05 | 0.58 | 1.00 | 0.92 | 1.09 | 0.99 | 1.02 | 0.90 | 1.17 | 0.75 | 1.01 | 0.82 | 1.24 | 0.95 | 1.01 | 0.89 | 1.16 | 0.84 |  |
| rs12982744 | 19 | 2177193 | G | C | 1.00 | 0.95 | 1.05 | 0.91 | 0.92 | 0.84 | 1.00 | 0.05 | 1.01 | 0.94 | 1.10 | 0.72 | 0.92 | 0.85 | 0.99 | 0.02 | 0.95 | 0.86 | 1.05 | 0.34 |  |
| rs7507204 | 19 | 3428834 | C | G | 0.95 | 0.89 | 1.01 | 0.08 | 0.85 | 0.72 | 1.01 | 0.07 | 0.98 | 0.89 | 1.07 | 0.60 | 1.09 | 0.96 | 1.25 | 0.19 | 0.97 | 0.90 | 1.04 | 0.43 |  |
| rs891088 | 19 | 7184762 | G | A | 1.01 | 0.95 | 1.08 | 0.69 | 1.11 | 0.97 | 1.27 | 0.14 | 0.94 | 0.84 | 1.05 | 0.29 | 0.83 | 0.73 | 0.95 | 0.006 | 1.04 | 0.92 | 1.19 | 0.51 |  |
| rs4072910 | 19 | 8644031 | G | C | 0.94 | 0.89 | 1.00 | 0.06 | 1.00 | 0.89 | 1.13 | 0.95 | 0.90 | 0.86 | 0.94 | <0.001 | 1.18 | 0.97 | 1.44 | 0.10 | 0.89 | 0.82 | 0.96 | 0.003 |  |
| rs2279008 | 19 | 17283303 | T | C | 1.00 | 0.97 | 1.04 | 0.88 | 1.00 | 0.87 | 1.14 | 0.95 | 1.00 | 0.94 | 1.05 | 0.92 | 1.24 | 0.92 | 1.67 | 0.16 | 0.99 | 0.87 | 1.13 | 0.85 |  |
| rs17318596 | 19 | 41937095 | A | G | 0.97 | 0.94 | 1.00 | 0.07 | 0.96 | 0.83 | 1.10 | 0.55 | 0.96 | 0.90 | 1.03 | 0.26 | 0.94 | 0.72 | 1.21 | 0.61 | 0.96 | 0.90 | 1.03 | 0.30 |  |
| rs1741344 | 20 | 4101800 | C | T | 1.01 | 0.92 | 1.11 | 0.90 | 1.18 | 0.93 | 1.49 | 0.17 | 0.92 | 0.87 | 0.96 | <0.001 | 1.00 | 0.80 | 1.24 | 0.97 | 1.06 | 1.00 | 1.13 | 0.05 |  |
| rs2145272 | 20 | 6626218 | G | A | 0.98 | 0.89 | 1.07 | 0.63 | 0.94 | 0.87 | 1.03 | 0.17 | 1.05 | 0.95 | 1.16 | 0.30 | 1.08 | 0.98 | 1.19 | 0.11 | 1.04 | 0.94 | 1.16 | 0.43 |  |
| rs7274811 | 20 | 32333181 | G | T | 1.02 | 0.93 | 1.13 | 0.64 | 0.99 | 0.84 | 1.18 | 0.95 | 1.07 | 0.97 | 1.18 | 0.15 | 0.95 | 0.77 | 1.17 | 0.62 | 1.01 | 0.92 | 1.10 | 0.81 |  |
| rs143384 | 20 | 34025756 | G | A | 1.00 | 0.95 | 1.06 | 0.98 | 0.95 | 0.85 | 1.06 | 0.39 | 1.01 | 0.95 | 1.07 | 0.68 | 1.03 | 0.95 | 1.13 | 0.47 | 1.02 | 0.98 | 1.07 | 0.35 |  |
| rs237743 | 20 | 47903019 | A | G | 0.98 | 0.93 | 1.04 | 0.54 | 0.96 | 0.90 | 1.03 | 0.26 | 1.03 | 0.96 | 1.10 | 0.45 | 0.97 | 0.89 | 1.07 | 0.56 | 0.92 | 0.87 | 0.98 | 0.009 |  |
| rs2834442 | 21 | 35690786 | A | T | 1.01 | 0.93 | 1.10 | 0.79 | 1.03 | 0.80 | 1.33 | 0.81 | 1.00 | 0.93 | 1.09 | 0.92 | 0.93 | 0.84 | 1.03 | 0.17 | 1.02 | 0.93 | 1.11 | 0.73 |  |
| rs4821083 | 22 | 33056341 | G | A | 0.95 | 0.87 | 1.02 | 0.17 | 1.00 | 0.91 | 1.10 | 1.00 | 0.97 | 0.85 | 1.09 | 0.58 | 0.79 | 0.60 | 1.06 | 0.11 | 0.94 | 0.81 | 1.09 | 0.43 |  |

**Supplementary Table 9: Association of 32 individual BMI SNPs with prostate cancer specific mortality after prostate cancer diagnosis. Hazard ratios indicate the effect of one extra height increasing allele.**

|  |  |  | Allele | |  |  | |  |  |  | |  |  |  | |  |  |  | |  |  |  | |  |
| --- | --- | --- | --- | --- | --- | --- | --- | --- | --- | --- | --- | --- | --- | --- | --- | --- | --- | --- | --- | --- | --- | --- | --- | --- |
|  |  |  | BMI | Other | Hazard | Confidence interval | | p- | Hazard | Confidence interval | | p- | Hazard | Confidence interval | | p- | Hazard | Confidence interval | | p- | Hazard | Confidence interval | | p- |
| RS id | Chr. | Position | increasing |  | ratio | Lower | Upper | value | ratio | Lower | Upper | value | ratio | Lower | Upper | value | ratio | Lower | Upper | value | ratio | Lower | Upper | value |
| rs2815752 | 1 | 72812440 | A | G | 0.96 | 0.90 | 1.01 | 0.13 | 0.97 | 0.89 | 1.05 | 0.46 | 0.98 | 0.93 | 1.03 | 0.39 | 1.00 | 0.88 | 1.12 | 0.94 | 1.01 | 0.93 | 1.10 | 0.79 |
| rs1514175 | 1 | 74991644 | A | G | 1.00 | 0.91 | 1.11 | 0.92 | 0.91 | 0.81 | 1.03 | 0.12 | 1.06 | 0.98 | 1.14 | 0.13 | 0.97 | 0.83 | 1.13 | 0.69 | 1.00 | 0.93 | 1.08 | 0.96 |
| rs1555543 | 1 | 96944797 | C | A | 1.00 | 0.97 | 1.02 | 0.95 | 1.00 | 0.87 | 1.14 | 0.96 | 1.02 | 0.95 | 1.09 | 0.57 | 1.18 | 1.06 | 1.32 | 0.003 | 0.96 | 0.92 | 1.00 | 0.04 |
| rs543874 | 1 | 177889480 | G | A | 0.96 | 0.89 | 1.03 | 0.25 | 0.96 | 0.77 | 1.19 | 0.69 | 0.97 | 0.86 | 1.10 | 0.66 | 0.85 | 0.65 | 1.12 | 0.26 | 1.03 | 0.95 | 1.12 | 0.43 |
| rs2867125 | 2 | 622827 | C | T | 1.04 | 0.94 | 1.15 | 0.49 | 0.90 | 0.74 | 1.10 | 0.29 | 1.09 | 0.96 | 1.25 | 0.17 | 0.94 | 0.78 | 1.12 | 0.47 | 1.02 | 0.90 | 1.15 | 0.78 |
| rs713586 | 2 | 25158008 | C | T | 1.04 | 1.01 | 1.08 | 0.02 | 1.03 | 0.96 | 1.11 | 0.44 | 1.01 | 0.93 | 1.08 | 0.89 | 0.94 | 0.84 | 1.04 | 0.24 | 1.03 | 0.97 | 1.10 | 0.32 |
| rs887912 | 2 | 59302877 | T | C | 1.01 | 0.95 | 1.07 | 0.81 | 0.91 | 0.79 | 1.05 | 0.22 | 1.01 | 0.94 | 1.10 | 0.76 | 1.01 | 0.88 | 1.15 | 0.94 | 0.97 | 0.88 | 1.06 | 0.51 |
| rs2890652 | 2 | 142959931 | C | T | 1.02 | 0.92 | 1.15 | 0.67 | 0.96 | 0.76 | 1.22 | 0.74 | 1.01 | 0.94 | 1.09 | 0.72 | 1.31 | 0.90 | 1.91 | 0.15 | 1.00 | 0.93 | 1.07 | 0.99 |
| rs13078807 | 3 | 85884150 | G | A | 0.99 | 0.90 | 1.10 | 0.89 | 0.91 | 0.79 | 1.05 | 0.18 | 1.01 | 0.89 | 1.15 | 0.86 | 0.85 | 0.78 | 0.94 | 0.001 | 1.00 | 0.90 | 1.10 | 0.95 |
| rs9816226 | 3 | 185834499 | T | A | 1.01 | 0.96 | 1.07 | 0.66 | 1.05 | 0.88 | 1.24 | 0.60 | 1.05 | 0.93 | 1.17 | 0.45 | 1.05 | 0.96 | 1.16 | 0.30 | 0.98 | 0.93 | 1.04 | 0.50 |
| rs10938397 | 4 | 45182527 | G | A | 0.97 | 0.93 | 1.01 | 0.14 | 0.93 | 0.78 | 1.11 | 0.41 | 0.96 | 0.89 | 1.03 | 0.22 | 1.04 | 0.89 | 1.22 | 0.62 | 0.97 | 0.92 | 1.03 | 0.31 |
| rs13107325 | 4 | 103188709 | T | C | 1.08 | 0.96 | 1.22 | 0.18 | 1.23 | 0.97 | 1.54 | 0.08 | 1.05 | 0.96 | 1.15 | 0.29 | 0.99 | 0.76 | 1.29 | 0.95 | 1.16 | 1.03 | 1.31 | 0.01 |
| rs2112347 | 5 | 75015242 | T | G | 1.00 | 0.96 | 1.04 | 0.85 | 0.99 | 0.90 | 1.09 | 0.82 | 0.98 | 0.92 | 1.05 | 0.54 | 1.02 | 0.89 | 1.17 | 0.75 | 0.97 | 0.86 | 1.08 | 0.56 |
| rs4836133 | 5 | 124332103 | A | C | 1.02 | 0.95 | 1.09 | 0.59 | 0.93 | 0.82 | 1.05 | 0.23 | 0.98 | 0.84 | 1.14 | 0.78 | 1.03 | 0.91 | 1.16 | 0.64 | 0.98 | 0.89 | 1.07 | 0.63 |
| rs206936 | 6 | 34302869 | G | A | 0.97 | 0.90 | 1.05 | 0.44 | 0.89 | 0.78 | 1.01 | 0.08 | 0.96 | 0.90 | 1.02 | 0.19 | 0.94 | 0.82 | 1.07 | 0.34 | 0.88 | 0.81 | 0.97 | 0.006 |
| rs987237 | 6 | 50803050 | G | A | 0.99 | 0.92 | 1.06 | 0.77 | 1.05 | 0.85 | 1.30 | 0.64 | 0.97 | 0.89 | 1.07 | 0.61 | 0.91 | 0.83 | 1.00 | 0.06 | 0.90 | 0.82 | 0.99 | 0.03 |
| rs10968576 | 9 | 28414339 | G | A | 1.03 | 0.98 | 1.08 | 0.19 | 0.95 | 0.88 | 1.02 | 0.13 | 1.07 | 1.01 | 1.14 | 0.03 | 1.13 | 0.95 | 1.33 | 0.17 | 0.99 | 0.92 | 1.07 | 0.86 |
| rs4929949 | 11 | 8604593 | C | T | 0.99 | 0.90 | 1.09 | 0.83 | 0.96 | 0.81 | 1.13 | 0.63 | 1.01 | 0.94 | 1.09 | 0.83 | 1.01 | 0.85 | 1.21 | 0.88 | 0.97 | 0.90 | 1.04 | 0.36 |
| rs10767664 | 11 | 27725986 | A | T | 0.96 | 0.89 | 1.02 | 0.17 | 0.91 | 0.80 | 1.04 | 0.17 | 0.91 | 0.82 | 1.00 | 0.04 | 0.81 | 0.58 | 1.12 | 0.20 | 0.95 | 0.85 | 1.06 | 0.34 |
| rs3817334 | 11 | 47650993 | T | C | 1.05 | 0.97 | 1.14 | 0.26 | 1.04 | 0.95 | 1.14 | 0.40 | 1.00 | 0.84 | 1.19 | 0.99 | 1.10 | 0.92 | 1.32 | 0.28 | 1.00 | 0.84 | 1.18 | 0.98 |
| rs7138803 | 12 | 50247468 | A | G | 0.99 | 0.94 | 1.05 | 0.78 | 0.98 | 0.88 | 1.11 | 0.79 | 0.97 | 0.93 | 1.02 | 0.31 | 1.19 | 1.12 | 1.27 | <0.001 | 0.99 | 0.89 | 1.09 | 0.79 |
| rs4771122 | 13 | 28020180 | G | A | 0.99 | 0.92 | 1.06 | 0.78 | 0.87 | 0.81 | 0.94 | <0.001 | 1.12 | 1.05 | 1.19 | <0.001 | 0.85 | 0.68 | 1.07 | 0.18 | 1.00 | 0.80 | 1.24 | 0.97 |
| rs11847697 | 14 | 30515112 | T | C | 0.90 | 0.81 | 0.99 | 0.03 | 1.17 | 0.81 | 1.68 | 0.39 | 0.82 | 0.63 | 1.07 | 0.15 | 0.70 | 0.45 | 1.09 | 0.12 | 0.91 | 0.72 | 1.15 | 0.44 |
| rs10150332 | 14 | 79936964 | C | T | 1.03 | 0.97 | 1.10 | 0.33 | 1.09 | 0.97 | 1.23 | 0.15 | 0.98 | 0.89 | 1.09 | 0.74 | 1.15 | 1.04 | 1.26 | 0.007 | 1.08 | 1.00 | 1.15 | 0.04 |
| rs2241423 | 15 | 68086838 | G | A | 1.03 | 0.95 | 1.11 | 0.51 | 1.13 | 1.00 | 1.28 | 0.05 | 0.98 | 0.85 | 1.13 | 0.79 | 1.23 | 1.03 | 1.47 | 0.02 | 0.99 | 0.85 | 1.14 | 0.84 |
| rs12444979 | 16 | 19933600 | C | T | 1.05 | 0.96 | 1.14 | 0.26 | 1.23 | 1.03 | 1.47 | 0.02 | 1.06 | 0.96 | 1.16 | 0.26 | 1.03 | 0.87 | 1.22 | 0.74 | 1.12 | 0.97 | 1.31 | 0.13 |
| rs7359397 | 16 | 28885659 | T | C | 0.97 | 0.93 | 1.01 | 0.17 | 1.02 | 0.92 | 1.12 | 0.75 | 0.96 | 0.87 | 1.07 | 0.49 | 0.90 | 0.75 | 1.08 | 0.27 | 1.03 | 0.96 | 1.10 | 0.39 |
| rs1558902 | 16 | 53803574 | A | T | 1.03 | 0.96 | 1.10 | 0.40 | 1.02 | 0.84 | 1.25 | 0.83 | 1.12 | 1.06 | 1.19 | <0.001 | 0.95 | 0.86 | 1.05 | 0.28 | 1.12 | 0.95 | 1.32 | 0.17 |
| rs571312 | 18 | 57839769 | A | C | 0.99 | 0.92 | 1.05 | 0.69 | 1.00 | 0.92 | 1.08 | 0.95 | 1.02 | 0.89 | 1.17 | 0.74 | 1.00 | 0.82 | 1.24 | 0.96 | 1.02 | 0.89 | 1.16 | 0.82 |
| rs29941 | 19 | 34309532 | G | A | 0.99 | 0.94 | 1.05 | 0.79 | 1.01 | 0.90 | 1.13 | 0.90 | 1.02 | 0.97 | 1.06 | 0.46 | 1.16 | 1.07 | 1.24 | <0.001 | 1.03 | 0.96 | 1.10 | 0.46 |
| rs2287019 | 19 | 46202172 | C | T | 0.98 | 0.91 | 1.06 | 0.64 | 1.02 | 0.91 | 1.15 | 0.71 | 0.97 | 0.91 | 1.04 | 0.37 | 1.14 | 0.98 | 1.33 | 0.10 | 0.97 | 0.87 | 1.08 | 0.58 |
| rs3810291 | 19 | 47569003 | A | G | 0.98 | 0.94 | 1.01 | 0.21 | 0.94 | 0.86 | 1.02 | 0.15 | 0.97 | 0.92 | 1.03 | 0.38 | 0.88 | 0.82 | 0.94 | <0.001 | 1.02 | 0.96 | 1.09 | 0.55 |

**Supplementary Table 10: Hazard ratio of all-cause and prostate cancer specific mortality of prostate cancer patients per one standard change in height or BMI genetic score. Additionally adjusted for PSA level, stage and grade.**

|  |  | Number |  | Adjusted^a^ | | | |
| --- | --- | --- | --- | --- | --- | --- | --- |
|  | Number of | of | Years at | Hazard | Confidence interval*^b^* | | P- |
|  | participants | failures | risk (1000s) | ratio*^c^* | Lower | Upper | value |
| **All-cause mortality** | | | |  |  |  |  |
| ***Height*** |  |  |  |  |  |  |  |
| All cases | 9,560 | 1,916 | 67.8 | 1.03 | 0.98 | 1.08 | 0.20 |
| ***BMI*** |  |  |  |  |  |  |  |
| All cases | 9,560 | 1,916 | 67.8 | 1.03 | 0.99 | 1.09 | 0.17 |
|  |  |  |  |  |  |  |  |
| **Prostate cancer specific mortality** | | | |  | | | |
| ***Height*** |  |  |  |  |  |  |  |
| All cases | 9,560 | 753 | 67.8 | 1.01 | 0.98 | 1.05 | 0.35 |
| ***BMI*** |  |  |  |  |  |  |  |
| All cases | 9,560 | 753 | 67.8 | 1.02 | 0.96 | 1.09 | 0.54 |

*^a^Adjusted for the first 8 principal components of population stratification, PSA level, stage and grade. ^b^Based in robust standard errors to account for within-study clustering. ^c^Change in hazard ratio per standard deviation change in height and BMI genetic risk score (standardised to mean zero standard deviation one).*

**Supplementary Figure 1: Association of a 1 standard deviation change in the weighted height genetic risk score with prostate cancer by study.**

*ES= Effect size on odds-ratio scale.* *Adjusted for the first 8 principal components of population stratification. Estimated using fixed effects by the metan command in Stata.*

**Supplementary Figure 2: Association of 1 standard deviation change in the weighted height genetic risk score with localised prostate cancer by study.**

*ES= Effect size on odds-ratio scale.* *Adjusted for the first 8 principal components of population stratification. Estimated using fixed effects by the metan command in Stata.*

**Supplementary Figure 3: Association of a 1 standard deviation change in the weighted height genetic risk score with advanced prostate cancer by study.**

*ES= Effect size on odds-ratio scale.* *Adjusted for the first 8 principal components of population stratification. Estimated using fixed effects by the metan command in Stata.*

**Supplementary Figure 4: Association of a 1 standard deviation change in the weighted height genetic risk score with low grade prostate cancer by study.**

*ES= Effect size on odds-ratio scale.* *Adjusted for the first 8 principal components of population stratification. Estimated using fixed effects by the metan command in Stata.*

**Supplementary Figure 5: Association of a 1 standard deviation change in the weighted height genetic risk score with high grade prostate cancer by study.**

*ES= Effect size on odds-ratio scale.* *Adjusted for the first 8 principal components of population stratification. Estimated using fixed effects by the metan command in Stata.*

**Supplementary Figure 6: Association of a 1 standard deviation change in the weighted BMI genetic risk score with prostate cancer by study.**

*ES= Effect size on odds-ratio scale.* *Adjusted for the first 8 principal components of population stratification. Estimated using fixed effects by the metan command in Stata.*

**Supplementary Figure 7: Association of a 1 standard deviation change in the weighted BMI genetic risk score with localised prostate cancer by study.**

*ES= Effect size on odds-ratio scale.* *Adjusted for the first 8 principal components of population stratification. Estimated using fixed effects by the metan command in Stata.*

**Supplementary Figure 8: Association of a 1 standard deviation change in the weighted BMI genetic risk score with advanced prostate cancer by study.**

*ES= Effect size on odds-ratio scale.* *Adjusted for the first 8 principal components of population stratification. Estimated using fixed effects by the metan command in Stata.*

**Supplementary Figure 9: Association of a 1 standard deviation change in the weighted BMI genetic risk score with low grade prostate cancer by study.**

*ES= Effect size on odds-ratio scale.* *Adjusted for the first 8 principal components of population stratification. Estimated using fixed effects by the metan command in Stata.*

**Supplementary Figure 10: Association of a 1 standard deviation change in the weighted BMI genetic risk score with high grade prostate cancer by study.**

*ES= Effect size on odds-ratio scale.* *Adjusted for the first 8 principal components of population stratification. Estimated using fixed effects by the metan command in Stata.*
